# Activation of ULK Kinase and Autophagy by GABARAP Trafficking from the Centrosome Is Regulated by WAC and GM130

**DOI:** 10.1016/j.molcel.2015.11.018

**Published:** 2015-12-17

**Authors:** Justin Joachim, Harold B.J. Jefferies, Minoo Razi, David Frith, Ambrosius P. Snijders, Probir Chakravarty, Delphine Judith, Sharon A. Tooze

**Affiliations:** 1 Molecular Cell Biology of Autophagy, The Francis Crick Institute, Lincoln’s Inn Fields Laboratories, 44 Lincoln’s Inn Fields, London WC2A 3LY, UK; 2 Mass Spectrometry, The Francis Crick Institute, Lincoln’s Inn Fields Laboratories, 44 Lincoln’s Inn Fields, London WC2A 3LY, UK; 3 Bioinformatics Core, The Francis Crick Institute, Lincoln’s Inn Fields Laboratories, 44 Lincoln’s Inn Fields, London WC2A 3LY, UK

## Abstract

Starvation-induced autophagy requires activation of the ULK complex at the phagophore. Two Golgi proteins, WAC and GM130, regulate autophagy, however their mechanism of regulation is unknown. In search of novel interaction partners of WAC, we found that GM130 directly interacts with WAC, and this interaction is required for autophagy. WAC is bound to the Golgi by GM130. WAC and GM130 interact with the Atg8 homolog GABARAP and regulate its subcellular localization. GABARAP is on the pericentriolar matrix, and this dynamic pool contributes to autophagosome formation. Tethering of GABARAP to the Golgi by GM130 inhibits autophagy, demonstrating an unexpected role for a golgin. WAC suppresses GM130 binding to GABARAP, regulating starvation-induced centrosomal GABARAP delivery to the phagophore. GABARAP, unlipidated and lipidated, but not LC3B, GABARAPL1, and GATE-16, specifically promotes ULK kinase activation dependent on the ULK1 LIR motif, elucidating a unique non-hierarchical role for GABARAP in starvation-induced activation of autophagy.

## Highlights

• WAC is required for ULK activation and autophagy and interacts with GM130
• WAC suppresses GM130 binding to GABARAP and activates autophagy
• GABARAP traffics from the pericentriolar matrix to forming autophagosomes
• WAC controls the non-hierarchical activation of ULK complex by centrosomal GABARAP

## Graphical Abstract

**Figure:**
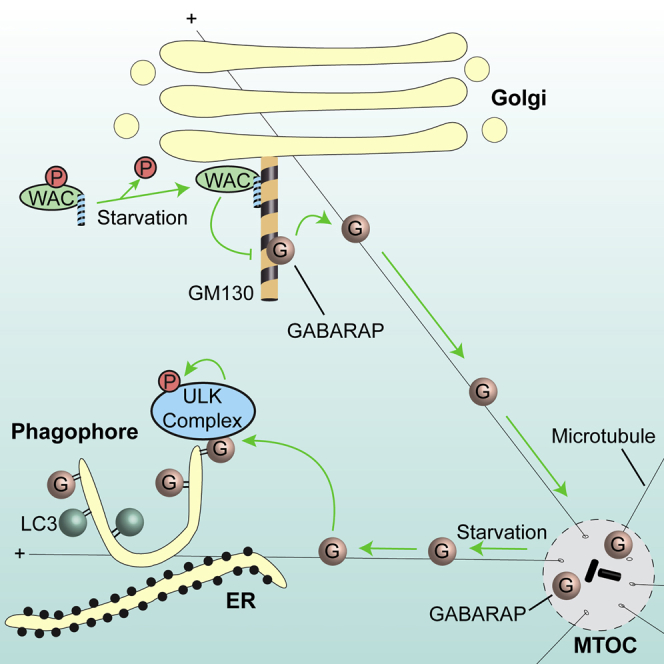

## Teaser

WAC and GM130 are two Golgi proteins that regulate autophagy. Joachim et al. show that WAC inhibits binding of the autophagy protein GABARAP to the vesicle tethering golgin GM130. This allows trafficking of the centrosomal pool of GABARAP to forming autophagosomes and GABARAP-mediated activation of the autophagy-initiating ULK1 kinase.

## Introduction

Cellular homeostasis requires apposition of anabolic and catabolic pathways acting in coordination to regulate protein synthesis, trafficking, and secretion. Autophagy, a conserved catabolic pathway, delivers cytosolic cargo, sequestered by autophagosomes, to the lysosome for degradation. Degraded cargo is recycled for protein synthesis and metabolism. Autophagosomes also sequester damaged organelles, misfolded proteins, or pathogens to maintain cell health and prevent infection.

The formation of autophagosomes occurs from subdomains of the ER, mediated by ATG9-positive vesicles, followed by activation and recruitment of the ULK kinase complex and the class III PtdIns(3) (phosphatidylinositol(3)) kinase complex containing BECLIN 1 and ATG14. Association of ATG14 to the ER facilitates production of PtdIns(3)P and establishes a DFCP1-rich omegasome from which the phagophore, the nascent autophagosome, forms (Lamb et al., 2013). Further expansion of the phagophore requires recruitment of lipidated Atg8 proteins via attachment of phosphatidylethanolamine (PE) on their C-terminal glycine by a ubiquitination-like pathway. The lipidation machinery is recruited to the phagophore membrane by the PtdIns(3)P-effector WIPI2B (Dooley et al., 2014).

The mammalian Atg8 family includes LC3A, LC3B, and LC3C, GABARAP, GABARAPL1, and GABARAPL2 (or GATE-16), which perform three functions in autophagy: first, to mediate expansion and closure of the phagophore membrane; second, as cargo receptors to recruit cytoplasmic cargo through the LIR (LC3-interacting region) motif (Slobodkin and Elazar, 2013); and third, as adaptors to recruit signaling and trafficking proteins and the autophagy machinery to the autophagosome (Birgisdottir et al., 2013, Stolz et al., 2014).

To uncover novel regulators of starvation-induced autophagy, we performed a siGenome screen (McKnight et al., 2012) and identified WAC (WW domain-containing adaptor with coiled coil), which is required for starvation-induced autophagy. WAC is in the nucleus and cytoplasm and associates with the Golgi complex. Nuclear WAC binds to RNA Polymerase II and the E3 ligase complex RNF20/40 to facilitate transcription-coupled H2B monoubiquitination at lysine 120 (Zhang and Yu, 2011), which is associated with sites of active transcription (Karpiuk et al., 2012). Golgi WAC interacts with the deubiquitinase VCIP135, which binds VCP/p97 (Totsukawa et al., 2011). Here, starting from the identification of GM130 (GOLGA2) as a novel interactor of WAC, we show that WAC is tethered to the Golgi by GM130, and while WAC positively regulates autophagy, GM130 is a negative regulator. GM130 is a coiled-coil domain *cis*-Golgi matrix tethering protein required for vesicle trafficking and Golgi maintenance through mitosis (Nakamura, 2010).

Furthermore, we show that WAC and GM130 interact with GABARAP and control its subcellular localization. GABARAP is on the Golgi and the pericentriolar matrix (PCM): tethering of GABARAP to the Golgi by GM130 inhibits autophagy, while the PCM pool of GABARAP contributes to autophagosome formation. WAC suppresses GM130 binding to GABARAP, regulating the PCM reservoir of GABARAP. GABARAP association with the PCM does not require lipidation. A photoconvertible GABARAP shows that PCM-associated GABARAP traffics to forming autophagosomes and GABARAP trafficking to the autophagosome requires WAC. Lastly, our data show that like WAC, GABARAP is required for ULK kinase activation and has a non-hierarchical function in formation of autophagosomes.

## Results

### WAC Drives Starvation-Induced Autophagy and ULK1 Kinase Activation

**Figure 1:**
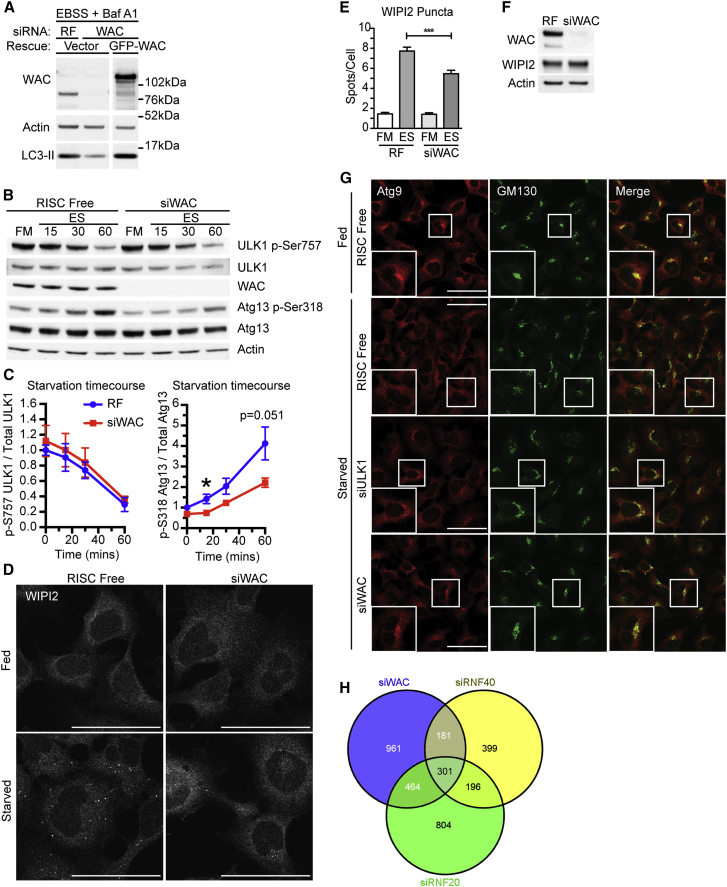
WAC Promotes Starvation-Induced Autophagy and ULK1 Kinase Activation (A) siRNA-resistant EGFP-WAC or vector was expressed in HEK293A cells treated for 72 hr with either RISC-free (RF) or WAC siRNA. After 2 hr starvation with EBSS and BafA1, cells were analyzed by immunoblot. (B) HEK293A cells treated with RF or WAC siRNA for 72 hr were incubated in full medium (FM) or EBSS (ES) for 15, 30, and 60 min. (C) Quantification of (B); statistical analysis using unpaired Student’s t test, mean ± SEM, n = 3 experiments, ^∗^p ≤ 0.05. (D) HEK293A cells were treated with RF or WAC siRNA for 72 hr then starved 2 hr, fixed, and labeled with WIPI2. Scale bars, 50 μm. (E) WIPI2 puncta in (D) were counted. Mean ± SEM from n = 2 experiments, >150 cells counted per condition, unpaired Student’s t test, ^∗∗∗^p ≤ 0.001. (F) HEK293A cells treated with RF or WAC siRNA for 72 hr before immunoblotting. (G) HEK293A cells were treated with RF, ULK1, or WAC siRNA for 72 hr, incubated in fed or 2 hr EBSS (Starved), and labeled for Atg9 and GM130. Scale bars, 50 μm. (H) Venn diagram of number of genes significantly (p < 0.05) up- or downregulated in HEK293A cells treated with WAC, RNF20, or RNF40 siRNA for 72 hr versus RF control. See also Figure S1 and Table S1. Excised lanes are indicated by a gap, and remaining lanes are from the same gel.

In confirmation of our previous results (McKnight et al., 2012), WAC depletion using siRNA reduced LC3 lipidation during starvation and caused an increase in SQSTM1 (better known as p62) levels under basal conditions (Figures S1A–S1C). Decreased LC3-II accumulation after WAC depletion was rescued with siRNA-resistant GFP-WAC (Figure 1A).

To understand the role of WAC during autophagy, we determined the stage WAC acted to promote autophagy. In nutrient-rich conditions, mTORC1 phosphorylates and represses ULK1 kinase activity (Chan, 2009). Starvation inhibits mTORC1, allowing ULK1 activation and phosphorylation of the ULK complex member ATG13 on Ser318 (Alers et al., 2012). A time course of starvation in WAC-depleted cells revealed that mTORC1 inactivation, measured by loss of phosphorylation of ULK1 at Ser757 and S6 at Ser240/244, was unaffected (Figures 1B–1C and Figure S1D). In contrast, ATG13 Ser318 phosphorylation was reduced after WAC depletion, suggesting that ULK1 activation, not mTORC1 deactivation, is impaired by loss of WAC.

We next assessed WIPI2 puncta formation, which correlates with the amount of PtdIns(3)P produced by the BECLIN 1, ATG14-containing class III PtdIns(3) kinase complex (Dooley et al., 2014) and is a measure of the activation of the autophagy-specific PtdIns(3) kinase. WIPI2 puncta formation, but not protein levels, was reduced by WAC depletion in starved or Torin-treated cells (Figures 1D–1F, and Figures S1E–S1H). Moreover, siRNA-resistant Myc-WAC could rescue WIPI2 puncta formation, and thus PtdIns(3) kinase activation, during starvation (Figure S1I).

Finally, starvation-induced ATG9 trafficking to forming autophagosomes, which is required for autophagy (Orsi et al., 2012, Young et al., 2006), was inhibited by WAC depletion. ATG9 remained localized in the Golgi region (Figure 1G) to an extent similar to that of ULK1 depletion. In short, during amino acid starvation WAC promotes ULK1 activation and the initiation of autophagosome formation.

### WAC Regulates Autophagy Independently of Its Nuclear Function

WAC localizes to the nucleus and the Golgi (Totsukawa et al., 2011, Zhang and Yu, 2011). To determine if WAC affects autophagy through its nuclear or cytoplasmic function, we performed a microarray analysis after knockdown of the nuclear complex of WAC/RNF20/RNF40. This generated an overlapping set of 301 potential gene targets that were significantly (p < 0.05) down- or upregulated by depletion of the nuclear complex (Figure 1H and Table S1). Analysis of the 301 hits (Table S1) revealed that p53 and p53 target genes (p = 0.00279) were significantly downregulated. This agrees with data that WAC regulates p53 targets and that RNF20 regulates p53 expression (Shema et al., 2008, Zhang and Yu, 2011). Autophagy, membrane trafficking, or lysosomal biogenesis pathways were not significantly up- or downregulated. Thus, unlike TFEB or ZKSCAN3 (Füllgrabe et al., 2014), the WAC/RNF20/40 complex does not regulate mRNA expression of the autophagy/lysosome pathway. Finally, knockdown of RNF40 did not affect LC3 lipidation or ULK1 signaling to ATG13 (Figures S1J and S1K).

### WAC Directly Interacts with GM130 through Its Conserved Coiled-Coil Domain

WAC interacts with Flag-HA-BECLIN 1 (Behrends et al., 2010) and GFP-BECLIN 1 (McKnight et al., 2012), but not endogenous BECLIN 1 (data not shown). To identify novel binding partners of WAC that regulate autophagy, we used pull-down experiments followed by mass spectrometry. A human *WAC* gene in a bacterial artificial chromosome (BAC) was modified with a C-terminal FLAP tag (Poser et al., 2008) (see Figure S2A) and used to generate a HeLa cell line expressing WAC-FLAP; all four isoforms of WAC were tagged and expressed at levels similar to those of endogenous WAC (Figures S2B and S2D). WAC-FLAP localizes to the nucleus and Golgi and binds RNF40 (Figures S2C and S2D). WAC immunoprecipitations from HEK293 cells, GFP immunoprecipitations from WAC-FLAP HeLa cells and mass spectrometry identified the *cis*-Golgi coiled-coil tether GM130 as a WAC interactor (Figures S2E–S2G), as well as the WAC interactors RNF20, RNF40, and UBE2A.

**Figure 2:**
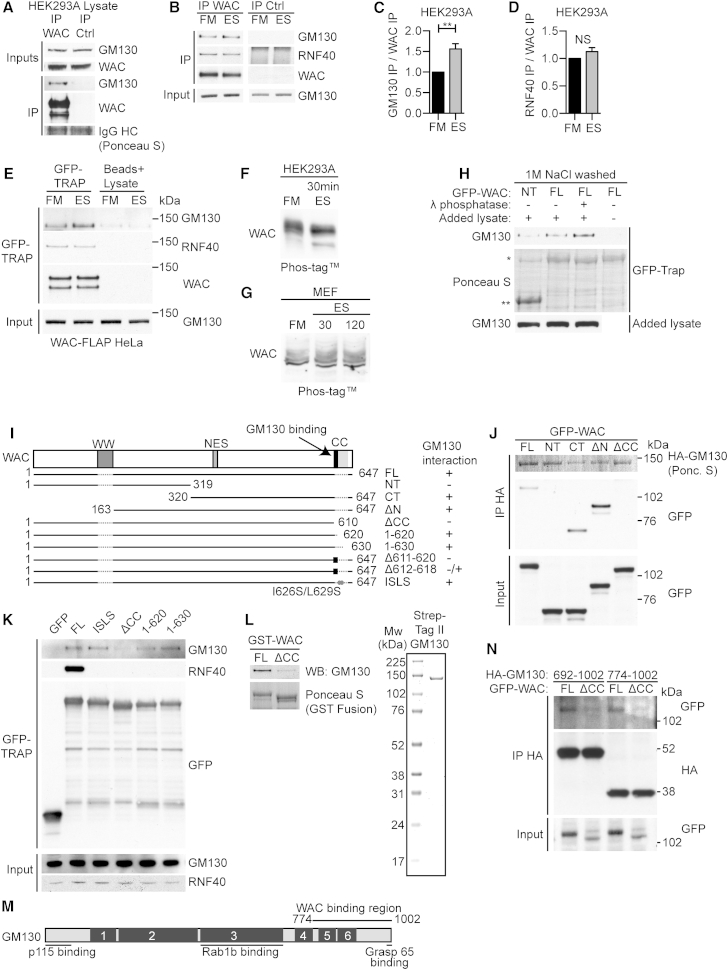
The WAC-GM130 Interaction Is Direct and Independent of RNF40 (A) Anti-WAC and anti-GFP immunoprecipitates analyzed by immunoblotting. HC, IgG heavy chain. (B) HEK293A cells in full medium (FM) or EBSS (ES) for 2 hr prior to lysis, followed by treatment as in (A). (C) Quantification of GM130 as in (B) after normalization to immunoprecipitated WAC. Statistical analysis using unpaired Student’s t test, mean ± SEM, n = 4, ^∗∗^p ≤ 0.01. (D) Quantification of RNF40 as in (B) after normalization to immunoprecipitated WAC. Mean ± SEM, n = 2. (E) GFP-TRAP pull down of HeLa WAC-FLAP cell followed by immunoblot. Cells in FM or EBSS (ES) for 2 hr prior to lysis. (F and G) WAC in FM or EBSS for 30 min in HEK293 (F) or 30 and 120 min in MEFS (G) were analyzed by Phos-tag SDS-PAGE. (H) GFP-TRAPs of GFP-WAC full length (FL) or aa1–319 (NT), salt washed, treated or not with lambda phosphatase, incubated with HEK293 lysate, and immunoblotted with anti-GM130. (I) Human WAC isoform 1 and deletion mutants used here: WW domain, putative nuclear export signal (NES), and C-terminal coiled-coil (CC) domain, including the GM130 binding region. GM130 binding ability is indicated. Black square, 10aa or 7aa heptad deletions. Grey circles, ISLS mutations. (J) HEK293A cells co-transfected with 3xHA-GM130 and EGFP-WAC FL, NT, aa320–647 (CT), aa163–647 (ΔN), or aa1–610 (ΔCC) immunoprecipitated with anti-HA and immunoblotted. (K) GFP-TRAP of cells expressing EGFP or EGFP-WAC FL, ISLS, ΔCC, 1–620, or 1–630 and immunoblot of GM130 and RNF40. (L) GST-WAC FL or GST-WAC ΔCC incubated with purified Strep-Tag II-GM130. GST-WAC monitored by Ponceau S staining. Right, colloidal Coomassie staining of Strep-Tag II-GM130. (M) Human GM130 isoform 1, boxes 1–6, coiled-coil domains. USO1 (p115), Rab1b, Grasp65, and WAC binding regions are indicated. (N) HEK293A cell lysates containing EGFP-WAC FL or ΔCC were mixed with HA-GM130 aa692–1,002 or HA-GM130 aa774–1,002 lysates, immunoprecipitated with anti-HA, and analyzed by immunoblotting. See also Figure S2. Excised lanes are indicated by a gap, and remaining lanes are from the same gel.

We confirmed binding of endogenous WAC to GM130 by co-immunoprecipitation (Figure 2A). Upon starvation of HEK293 or HeLa WAC-FLAP cells, the amount of GM130, but not RNF40, interacting with WAC or WAC-FLAP increased (Figures 2B–2E). As WAC is phosphorylated (Xu and Arnaout, 2002) we asked if phosphorylation regulates the WAC-GM130 interaction. Using Phos-tag SDS-PAGE gels after 30 min starvation, WAC migrated faster, suggesting that WAC is dephosphorylated upon starvation (Figures 2F and 2G). To test if dephosphorylation enhanced the WAC-GM130 interaction, we salt washed GFP-WAC to remove bound proteins, including GM130, and treated with lambda phosphatase (Figure 2H). Dephosphorylated salt washed GFP-WAC had enhanced binding to endogenous GM130 compared to untreated salt washed GFP-WAC or N-terminal WAC (see Figure 2J). These results suggest that WAC is dephosphorylated during starvation and this enhances its ability to bind GM130.

The highly conserved WAC C-terminal coiled-coil (CC) domain was required for interaction with GM130 (Figures 2I–2J and Figure S2H). Truncation analyses of WAC revealed that a 10 amino acid sequence in the WAC CC domain (aa610–620) containing one heptad (aa612–618) was required for GM130 binding (Figure 2K and Figure S2I). RNF20/40 bind the C terminus of WAC including the CC domain (Zhang and Yu, 2011). Isoleucine 626 and leucine 629, at the “a” and “d” positions of a CC heptad respectively (I626S/L629S mutant), are essential for binding to RNF40 but not GM130 (Figure 2K). Finally, recombinant GST-WAC bound directly to purified GM130 and required the WAC CC domain (Figure 2L).

GM130 interacts with proteins involved in Golgi trafficking including USO1 (p115), RAB1B, RAB33B, and GRASP65 (Nakamura, 2010). Truncations of GM130 showed that full-length WAC, but not WAC ΔCC, binds to the C-terminal of GM130 at aa774–1002. This includes the fifth and sixth CC domains of GM130 as well as the GRASP65 binding site (Figures 2M and 2N).

### GM130 Tethers WAC to the Golgi, and the Interaction Promotes Autophagosome Formation

**Figure 3:**
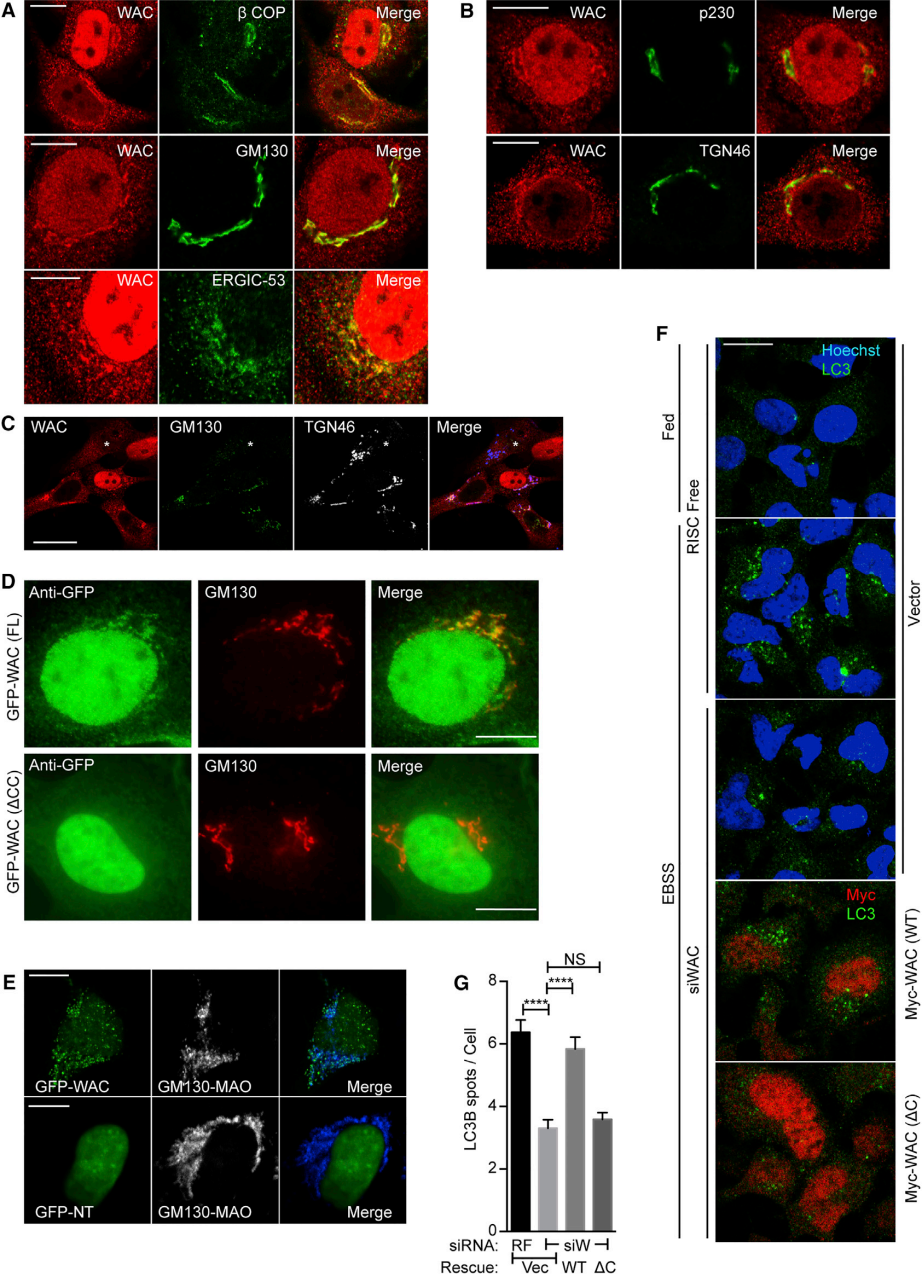
GM130 Tethers WAC to the Golgi, and the Interaction Is Required for Autophagosome Formation (A and B) WAC localized with βCOP, GM130, or ERGIC-53 (A) or p230 and TGN46 (B). Scale bars, 10 μm. (C) WAC, TGN46, and GM130 in GM130-depleted cells. (^∗^) cell depleted of GM130. Scale bars, 25 μm. (D) EGFP-WAC FL or ΔCC expressed after WAC depletion, labeled with indicated antibodies for epifluorescence microscopy. (E) EGFP-WAC (FL) or aa1–319 (NT) co-expressed with GM130-ΔCterm-HA-MAO labeled with anti-HA. Scale bars, 10 μm. (F) RF or WAC (siW) siRNA-treated cells for 72 hr transfected with vector (Vec), Myc-WAC (WT), or Myc-WAC aa1–610 (ΔC), starved for 2 hr with EBSS. LC3B puncta were analyzed by confocal microscopy. (G) LC3B puncta from (F), Mean ± SEM of n = 3, >400 cells counted per condition, unpaired Student’s t test, ^∗∗∗∗^p ≤ 0.0001. See also Figure S3.

WAC colocalizes with GM130 (Totsukawa et al., 2011), and in addition with the COPI coat protein βCOP and the ERGIC marker ERGIC-53 (Figure 3A), but less with the TGN markers p230 (GOLGA4) and TGN46 (Figure 3B). To test if GM130 is required for Golgi localization of WAC, we knocked down GM130. In cells with no detectable GM130, we never saw juxtanuclear WAC localization, even though the TGN remained intact (Figure 3C). GFP-WAC but not ΔCC WAC, localized to the Golgi region (Figure 3D). In addition, we performed a knockdown rescue experiment. In WAC knockdown cells, siRNA-resistant GFP-tagged full-length WAC but not ΔCC WAC, was detected on the Golgi (Figure S3A). We conclude that GM130 tethers WAC to the Golgi.

Using the recently developed mitochondrially targeted golgins that can tether vesicles to mitochondria (Wong and Munro, 2014) (see Figure S3B), we show that GM130-MAO can target GFP-WAC to mitochondria but GFP-WAC NT remained exclusively nuclear (Figure 3E). We next asked if the Golgi and nuclear pools of WAC are exchangeable. WAC has a putative nuclear export signal (NES) (Figure 2I), so we inhibited CRM-1-dependent nuclear export using leptomycin B (LMB) (Figure S3C). In contrast to control and 2 hr LMB, 24 hr LMB inhibited GFP-WAC targeting to the mitochondria. p62 was retained in the nucleus after LMB treatment, as expected (Pankiv et al., 2010). This suggests that in contrast to the rapid nuclear shuttling of p62, WAC is retained in the cytoplasm by GM130 and export of nuclear WAC is required to maintain the cytoplasmic WAC population.

Finally, knockdown and rescue experiments showed that the GM130-interacting CC domain of WAC was required to rescue LC3B spot formation under starvation conditions (Figures 3F and 3G), implying that the WAC-GM130 interaction promotes autophagosome formation.

### GM130 Is a Negative Regulator of Autophagy and Interacts with GABARAP

**Figure 4:**
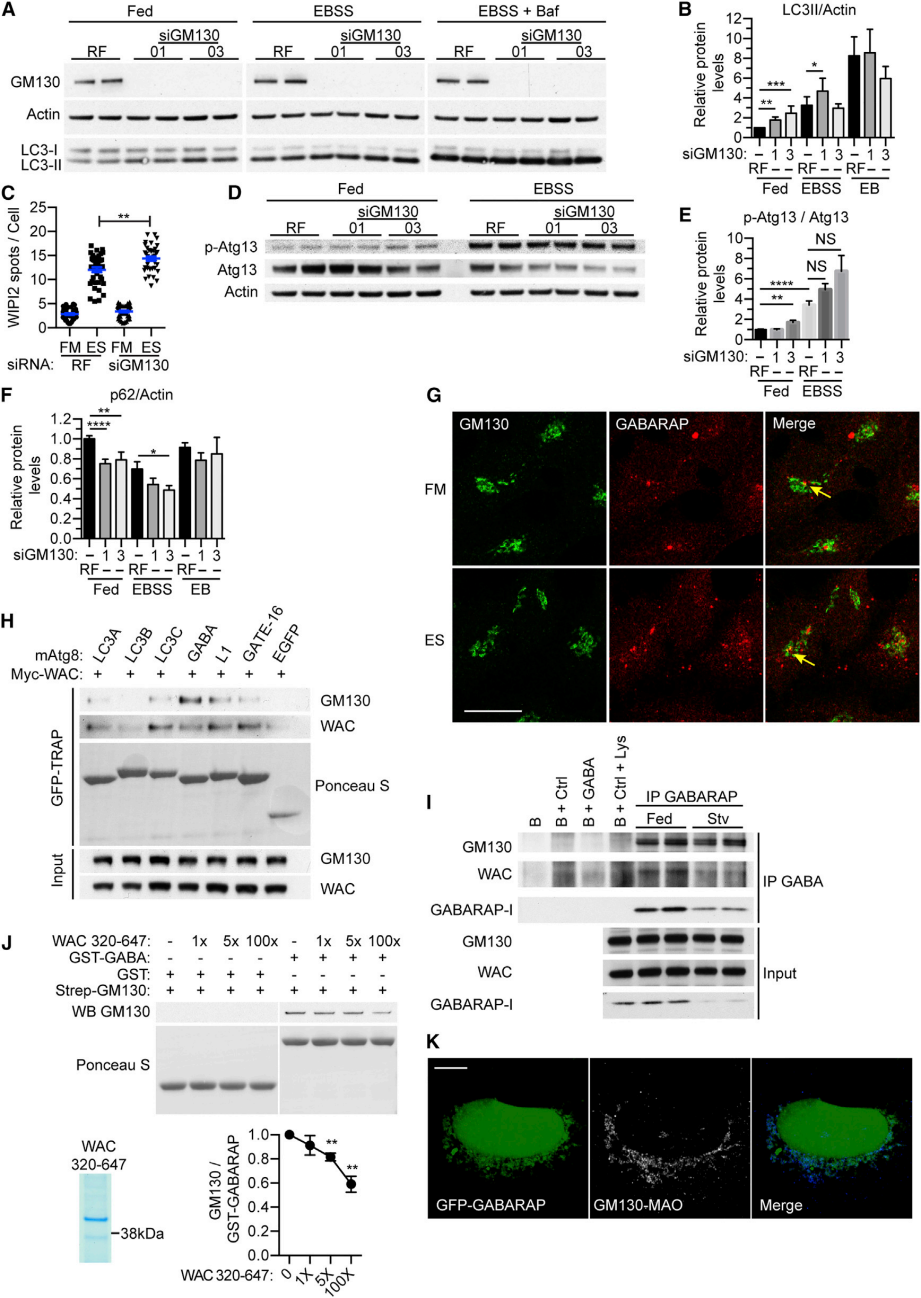
GM130 Is a Negative Regulator of the Early Stages of Autophagy and Interacts with GABARAP (A) HEK293A cells treated with RF or GM130 siRNA-01 or -03 incubated in full medium (FM), or EBSS (ES) with or without BafA for 2 hr. (B) LC3-II levels from (A). Mean ± SEM of n = 4, Mann-Whitney test, ^∗^p ≤ 0.05. (C) HEK293A cells treated with RF or GM130 siRNA incubated in full medium (FM) or EBSS (ES) for 2 hr. Statistical analysis using unpaired Student’s t test, mean ± SEM, n = 3, ^∗∗^p ≤ 0.01. 30 fields of cells were analyzed per condition. (D) HEK293A cells treated with RF or GM130 siRNA-01 or -03 incubated in full medium (FM) or EBSS (ES) for 2 hr and immunoblot. (E) Quantification of (D). Statistical analysis using unpaired Student’s t test, mean ± SEM, n = 5, ^∗∗^p ≤ 0.01. (F) p62 degradation from Figure S4A, mean ± SEM of n = 5, Mann-Whitney test, ^∗^p ≤ 0.05. (G) HEK293A cells in FM or EBSS for 2 hr. Arrows indicate colocalization. Scale bars, 20 μm. (H) Myc-WAC and EGFP, EGFP-LC3A, LC3B, LC3C, GABARAP, GABARAPL1, or GATE-16 co-expressed, followed by GFP-TRAP and immunoblot. (I) Immunoprecipitation of GABARAP from fed or starved cells and immunoblot analysis. B, beads; Ctrl, Anti-GFP; GABA, Anti-GABARAP. (J) Purified Strep II-GM130 was incubated with recombinant WAC 320–647 before binding to recombinant GST or GST-GABARAP beads and immunoblot. Statistical analysis using unpaired Student’s t test, mean ± SEM, n = 3. ^∗∗^p ≤ 0.01. (K) Co-expressed EGFP-GABARAP and GM130-ΔCterm-HA-MAO labeled with anti-HA. Scale bars, 10 μm. See also Figure S4. Excised lanes are indicated by a gap, and remaining lanes are from the same gel.

Loss of GM130 affects cell growth and increases autophagy in tumor cells and in a lung cancer mouse model (Chang et al., 2012). Similarly, in HEK293 cells depletion of GM130 increased LC3-II lipidation in both fed and starved cells, increased WIPI2 spots and basal ULK1 activation, and caused a significant decrease of p62 levels (Figures 4A–4F and Figure S4A). Overexpression of HA-GM130 reduced LC3-II levels but had no effect on p62 levels or WIPI2 spots (Figures S4B–S4E). These data imply that GM130 functions as a negative regulator of autophagy.

To probe the link between GM130 and autophagy, we investigated the localization of GM130 relative to autophagosome markers. We examined GABARAP, which localizes to the Golgi complex (Shpilka et al., 2011), and saw that GM130 in fed cells was distributed around an enlarged GABARAP structure, as well as occasionally colocalized with starvation-induced GABARAP puncta (Figure 4G).

Do GM130 and GABARAP, or other Atg8 homologs interact? Immunoprecipitation of a panel of mammalian Atg8 homologs revealed that GM130 preferentially bound GFP-GABARAP, whereas less binding was seen with GFP-LC3 family members (Figure 4H). WAC also interacted with the Atg8s including GFP-GABARAP. Importantly, WAC and GM130 interact with endogenous GABARAP (Figure 4I). No direct GABARAP-WAC interaction could be detected (Figures S4F–S4H), however, GM130 and GABARAP interact directly, and increasing amounts of WAC disrupt this interaction (Figure 4J). Additionally, mitochondrially targeted GM130 recruits GFP-GABARAP (Figure 4K) and endogenous GABARAP to the mitochondria (Figure S4I).

### GABARAP Localizes to the Centrosome and This Is Controlled by Nutrient Status and Microtubules

**Figure 5:**
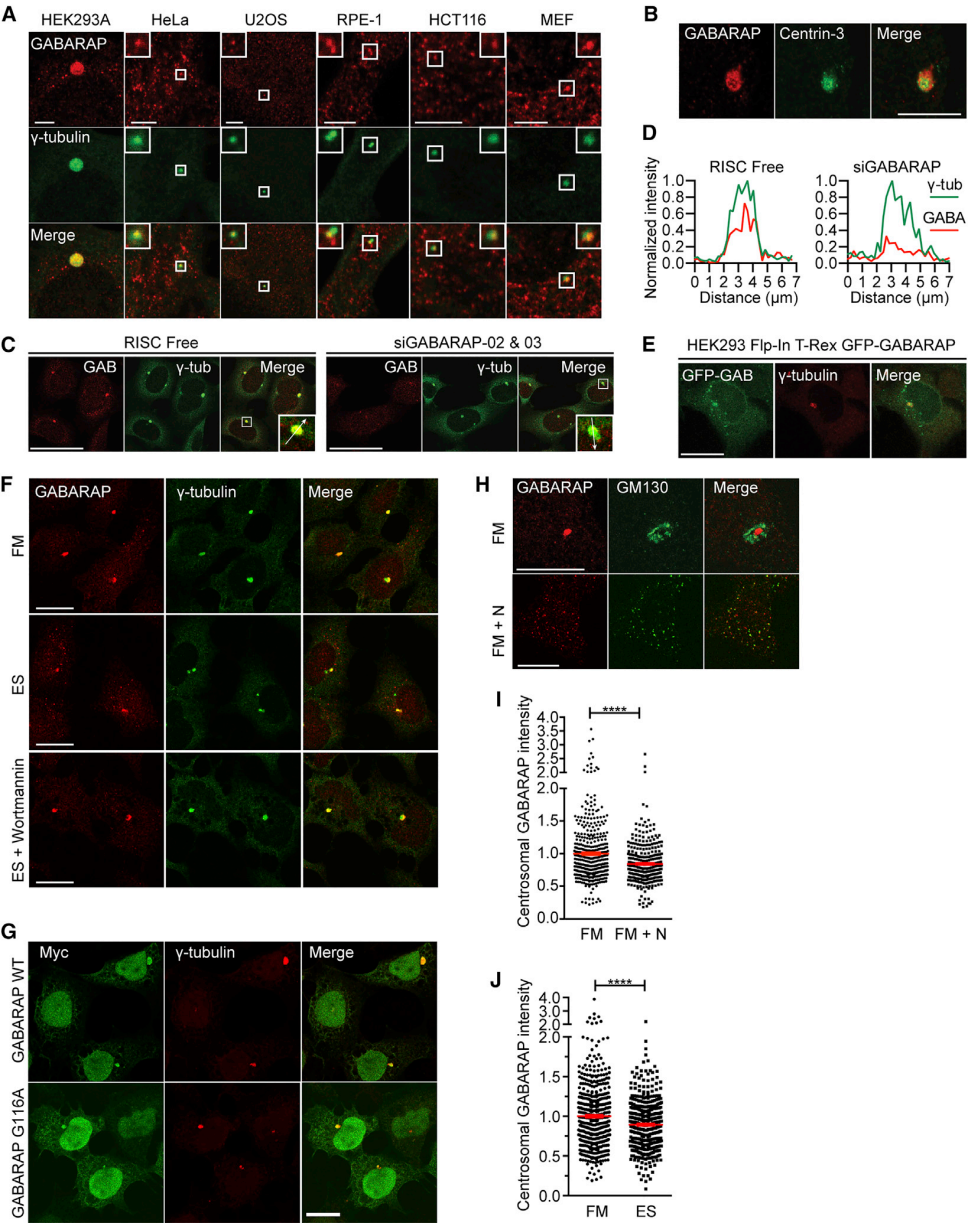
Non-lipidated GABARAP Localization at the Centrosome Is Regulated by Starvation and Microtubules (A) HEK293A, HeLa, U2OS, RPE-1, HCT116, and MEF cells were labeled as indicated. Scale bars, 5 μm. (B) HEK293A cells labeled as indicated. Scale bars, 10 μm. (C) HEK293A cells were treated for 72 hr with RF or GABARAP siRNAs and labeled as in (A). Scale bars, 50 μm. Inset, line scan. (D) Line scans of (C). (E) GFP-GABARAP expression in HEK293 Flp-In T-Rex cells after tetracycline for 24 hr. Scale bars, 20 μm. (F) HEK293A cells incubated in FM, EBSS (ES), or EBSS with wortmannin for 2 hr. Scale bars, 20 μm. (G) HEK293A cells expressing Myc-GABARAP or G116A mutant labeled as indicated. Scale bars, 20 μm. (H) HEK293A cells in FM or FM plus nocodazole (FM + N) for 5 hr. Scale bars, 20 μm. (I) Quantification of centrosomal GABARAP as in (H). Mean ± SEM of n = 3, >300 cells counted per condition, unpaired Student’s t test, ^∗∗∗∗^p ≤ 0.0001. (J) Quantification of centrosomal GABARAP after 2 hr with FM or EBSS. Mean ± SEM of n = 3, >420 cells counted per condition, unpaired Student’s t test, ^∗∗∗∗^p ≤ 0.0001. See also Figure S5.

**Figure 6:**
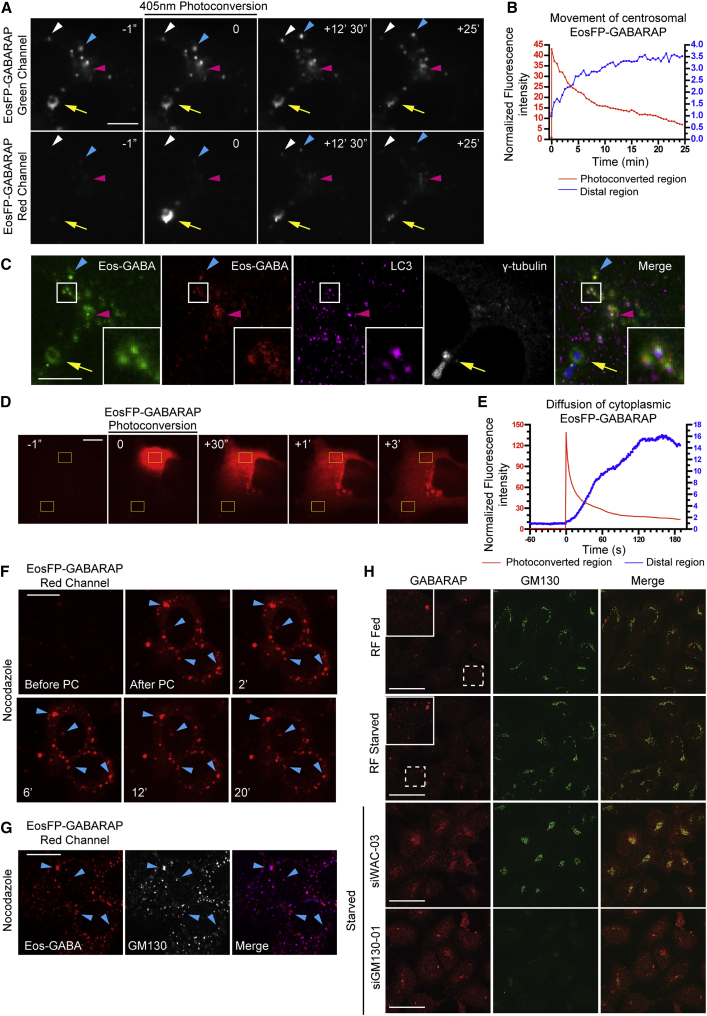
Centrosomal GABARAP Contributes to Autophagosome Formation (A) EosFP-GABARAP in starved cells was imaged every 30 s using a swept field confocal microscope. 5 × 1 s images were captured prior to photoconversion (PC). PC moment is set to 0 s. Yellow arrow, photoconverted region. Magenta arrowhead, distal region. White and blue arrows track defined puncta. Scale bars, 10 μm. (B) Quantification of fluorescence intensity from video in (A). PC region marked by yellow arrow and distal region marked by magenta arrowhead in (A). Intensity 5 s prior to PC moment is set to 1 for normalization. (C) Confocal microscopy performed on cell from video in (A). After imaging, cells were fixed and stained for LC3 and γ-tubulin. Arrows and arrowheads correspond to structures shown in (A). Scale bars, 10 μm. (D) EosFP-GABARAP as in (A). PC moment is set to 0 s. Yellow box (top), photoconverted region. Yellow box (bottom), distal region. Scale bar, 10 μm. (E) Quantification of intensities from boxed regions in (D). Intensity 60 s prior to PC moment is set to 1 for normalization. (F) EosFP-GABARAP expressing cells in FM with nocodazole for 2 hr, then incubated in EBSS with nocodazole and imaged. Images captured every 3 s. Blue arrows track defined puncta. Scale bars, 20 μm. (G) Confocal microscopy performed on cells from video in (F). After imaging, cells were fixed and stained for GM130. Arrows correspond to structures shown in (F). Scale bars, 20 μm. (H) HEK293A cells were treated with RF, WAC, or GM130 siRNAs for 72 hr and incubated with full medium (Fed) or EBSS (Starved) for 2 hr. Scale bars, 50 μm. See also Figure S6 and Movies S1 and S2.

Based on the localization and shape of the enlarged GABARAP-positive structure, we asked if it was positive for γ-tubulin, a marker for the PCM of the centrosome (Oakley, 2000). Using different GABARAP antibodies, we saw colocalization of GABARAP with γ-tubulin in HEK293A cells; we refer to this pool as centrosomal GABARAP (Figure 5A and Figure S5A). In five other cell lines, GABARAP and γ-tubulin also colocalized (Figure 5A). These GABARAP structures were not LC3B positive (see Figure 6C). Centrosomal GABARAP also localized around Centrin-3, a centriolar marker (Middendorp et al., 2000) (Figure 5B). Furthermore, in HEK293 cells with an enlarged PCM, knockdown of GABARAP reduced centrosomal GABARAP (Figures 5C and 5D). Using a tetracycline-inducible cell line, we confirmed that GFP-GABARAP colocalized with γ-tubulin (Figure 5E). Centrosomal GABARAP is not an aggresome, as it is negative for ubiquitin and p62, even upon proteasome inhibition with MG132 (Figures S5B and S5C). Wortmannin, an inhibitor of starvation-induced GABARAP-positive autophagosomes, did not affect GABARAP localization at the centrosome (Figure 5F). Moreover, the GABARAP G116A mutant, which is not lipidated (Figure S5D), also localized to the centrosome (Figure 5G).

We asked if centrosomal GABARAP was stably associated with the PCM. GABARAP associates with microtubules (Wang and Olsen, 2000), so we disassembled microtubules with nocodazole (Figure 5H and Figures S5E and S5F) and found that centrosomal GABARAP was significantly (p < 0.0001) reduced (Figure 5I) and there was a concomitant increased colocalization of GABARAP with Golgi mini-stacks (Figure 5H and Figure S5F). As expected, GABARAP relocalized to nocodazole-induced Golgi mini-stacks during wortmannin treatment (Figure S5F). In addition, centrosomal GABARAP dissociates during metaphase (Figure S5G).

After starvation, we saw a highly significant (p < 0.0001) decrease in centrosomal GABARAP signal and concurrent formation of GABARAP puncta (Figures 5F and 5J), suggesting GABARAP relocalizes from the centrosome to autophagosomes during starvation. In contrast to nocodazole treatment, dissembling the Golgi with Brefeldin A (BFA) (Figure S5H) did not diminish centrosomal GABARAP. Importantly, as BFA does not affect autophagic flux (Weidberg et al., 2010), GABARAP formed starvation-induced puncta even in the presence of BFA.

We conclude that centrosomal GABARAP is dynamic and is affected by disruption of microtubules or starvation, and that its association with the PCM and the Golgi is not dependent on lipidation but mediated by protein interactions.

### Centrosomal GABARAP Contributes to Autophagosome Formation

GABARAPs are essential for the final expansion or closure of the autophagosome (Weidberg et al., 2010). Given this, we suggest that centrosomal GABARAP acts as a reservoir for expansion of forming autophagosomes. To probe the dynamics of centrosomal GABARAP, we attached a photoconvertible fusion protein, EosFP, to the N terminus of GABARAP. EosFP is converted from green to red fluorescence upon excitation with UV light (Wiedenmann et al., 2004). EosFP-GABARAP colocalized with γ-tubulin and WIPI2 in starved HEK293 cells (Figure S6A), and EosFP-GABARAP formed rings around γ-tubulin, which are visible in live cells (Figure 6A, C and Figures S6B and S6C).

In starved live HEK293 cells, centrosomal green EosFP-GABARAP was photoconverted to red, and GABARAP-positive red spots formed while the intensity of red centrosomal EosFP-GABARAP decreased (Figure 6A, Figure S6B and Movie S1). Translocation of the photoconverted centrosomal EosFP-GABARAP to distal regions was monitored by quantifying fluorescence intensities (Figure 6B). Like autophagosomes, EosFP-GABARAP puncta were highly mobile (Kimura et al., 2008) and additionally made transient interactions with centrosomal EosFP-GABARAP (Movie S2). Strikingly, peripheral green EosFP-GABARAP spots (that existed prior to photoconversion) acquired centrosomally derived red photoconverted EosFP-GABARAP (Figure 6A and Figure S6B), implying that forming and expanding autophagosomes acquire GABARAP derived from the PCM. After time-lapse microscopy, the cells were fixed and stained with LC3 and γ-tubulin (Figure 6C and Figure S6C). Correlative confocal microscopy confirmed that the photoconverted pool of GABARAP was at the centrosome and that photoconverted centrosomal GABARAP had moved to peripheral puncta, many of which were LC3 positive (Figure 6C).

In contrast to the centrosomal pool, cytosolic EosFP-GABARAP diffused within seconds across the cell (Figures 6D–6E and Figures S6D and S6E), indicating that the red GABARAP-puncta in Figures 6A and S6B are derived from a centrosomal pool and not from unintentional photoconversion of the cytosolic pool.

Next, we treated live HEK293 cells expressing EosFP-GABARAP with nocadozole; EosFP-GABARAP was lost from the centrosome and dispersed into puncta (Figure 6F), which were not dynamic over a 20 min period after photoconversion. Correlative confocal microscopy revealed that similar to endogenous GABARAP (Figure 5H), EosFP-GABARAP was retained on Golgi mini-stacks (Figure 6G). Thus, without microtubules, centrosomal GABARAP relocalizes to the Golgi, becomes immobile and does not contribute to autophagosome formation.

### WAC Inhibits GM130 Tethering of GABARAP to Maintain the Centrosomal GABARAP Reservoir and GABARAP-Mediated ULK1 Activation

**Figure 7:**
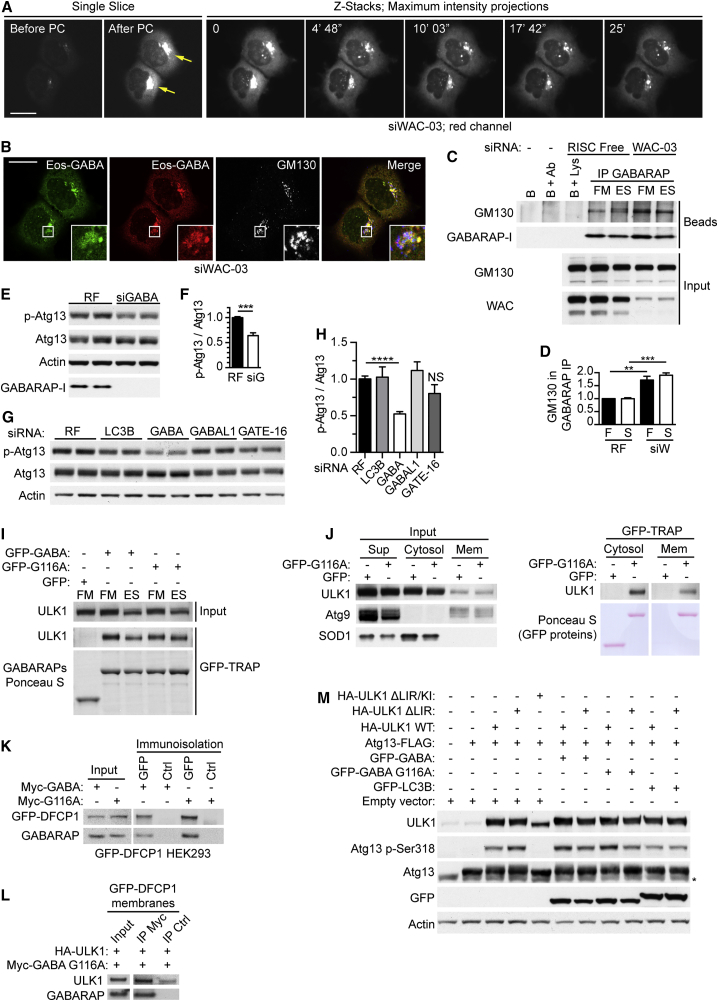
WAC Inhibits GM130 Binding of GABARAP and GABARAP-Mediated ULK1 Activation (A) EosFP-GABARAP expressed in HEK293A cells treated with WAC siRNA for 72 hr. Live-cell imaging under starvation conditions. During photoconversion (PC), images from a single z slice were acquired every 0.4 s. After PC, z stacks were acquired every 13 s. Yellow arrows denote PC regions. Scale bars, 20 μm. (B) After time-lapse imaging, cells shown in (A) were fixed and stained for GM130. Scale bars, 20 μm. (C) HEK293A cells were treated with RF or WAC siRNA for 72 hr and incubated with full medium (F) or EBSS (S) for 2 hr followed by GABARAP immunoprecipitation and immunoblotting. B, beads; Ab, GABARAP antibody; Lys, lysate. (D) Quantification of (C), Student’s t test, ^∗∗^p ≤ 0.01. Mean ± SEM of n = 3. (E) HEK293A cells treated with RF or GABARAP siRNA for 72 hr, incubated in EBSS for 2 hr followed by immunoblot. p-Atg13, pSer318. (F) Quantification of (E), Student’s t test, ^∗∗∗^p ≤ 0.001. Mean ± SEM of n = 3. (G) HEK293A cells treated with RF, LC3B, GABARAP, GABARAPL1, or GATE-16 siRNAs for 72 hr before 2 hr incubation in EBSS and immunoblot. p-Atg13, pSer318. (H) Quantification of (G), Student’s t test, ^∗∗∗∗^p ≤ 0.0001. Mean ± SEM of n = 3. (I) HEK293A cells expressing GFP, GFP-GABARAP, or GFP-GABARAP G116A were incubated in FM or ES for 2 hr prior to GFP-TRAP and immunoblot. (J) HEK293A cells expressing GFP or GFP-GABARAP G116A were incubated in EBSS for 2 hr prior to subcellular fractionation, GFP-TRAP, and immunoblot. Atg9 marks the membrane fraction and SOD1 the cytosol. (K) HEK293 cells stably expressing GFP-DFCP1 transfected with Myc-GABARAP or G116A mutant were starved for 2 hr in EBSS, and the GFP-DFCP1 compartment was immunoisolated prior to immunoblot. Flag M2 antibody was used as a control. (L) The GFP-DFCP1 compartment was isolated from HEK293 cells co-expressing the indicated constructs and starved in EBSS for 2 hr. Solubilised membranes were subjected to immunoprecipitation with anti-Myc or anti-FLAG M2 control. (M) HEK293A cells expressing indicated proteins analyzed by immunoblot. ^∗^, non-specific band. KI, kinase-inactive ULK1. Quantification shown in Figure S7F. See also Figure S7 and Movie S3. Excised lanes are indicated by a gap, and remaining lanes are from the same gel.

Since WAC and GM130 form a complex with GABARAP (Figures 4H–4J), we asked if WAC and GM130 controlled the localization of GABARAP to the PCM or forming autophagosomes. Centrosomal GABARAP co-localized with γ-tubulin in both fed and starved cells, and in starved cells GABARAP-positive autophagosomes appeared (Figures 6H and S6F). In starvation, WAC knockdown caused a striking accumulation of GABARAP on the Golgi and ERGIC and loss of centrosomal GABARAP (Figure 6H and Figures S6F and S6G). To investigate GABARAP dynamics in WAC-depleted cells, we used EosFP-GABARAP. As expected, EosFP-GABARAP accumulated on and around the Golgi (Figures 7A and 7B). Photoconversion and time-lapse microscopy showed that the pool of Golgi-accumulated EosFP-GABARAP is relatively immobile and does not form cytoplasmic GABARAP spots (Figures 7A and 7B, Movie S3). Importantly, in WAC knockdown cells there was an increase in the GM130-GABARAP interaction (Figures 7C and 7D). Thus, in the absence of WAC, GABARAP, likely non-lipidated, relocalizes from the centrosomal pool to the Golgi and ERGIC, is tethered by GM130, and does not contribute to autophagosome formation.

GM130 regulates centrosome morphology and function (Kodani and Sütterlin, 2008), and after GM130 knockdown, we saw an increase in the size and intensity of the centrosomal GABARAP pool (Figure 6H and Figure S6F). These data suggest that WAC and GM130 control the localization of centrosomal GABARAP in a reciprocal manner and that in cells WAC negatively regulates GABARAP-GM130 binding.

As WAC depletion reduced ATG13 phosphorylation (Figures 1B–1C and Figure S1D), we asked if GABARAP knockdown also attenuates ULK1 activation. ATG13 p-Ser318 levels were significantly decreased after depletion of GABARAP but not LC3B, GABARAPL1, or GATE-16 (Figures 7E–7H and Figure S7A). Centrosomal GABARAP is not lipidated, and non-lipidated GABARAP interacts constitutively with ULK1 (Figure 7I) in the cytosol and on membranes in a LIR-dependent manner (Figure 7J and Figures S7B and S7C). To define the membrane compartment harboring the ULK1-GABARAP complex, we immunoisolated DFCP-1-positive omegasomes and phagophores from starved cells. These DFCP-1 structures were positive for endogenous ULK1, WIPI2, GABARAP, and overexpressed wild-type ULK1, but not ΔLIR ULK1 (Figures S7D and S7E). GABARAP G116A was also found in similar amounts to GABARAP (Figure 7K), and immunoprecipitation of G116A revealed that it interacts with ULK1 on GFP-DFCP1 membranes (Figure 7L).

Out of the Atg8 family, ULK complex preferentially binds GABARAP through a LIR motif (Alemu et al., 2012, Kraft et al., 2012). Overexpression of HA-ULK1 in fed HEK293 cells was sufficient to drive ATG13-FLAG phosphorylation even under basal conditions and when the ULK1 LIR was mutated (Figure 7M). However, in the presence of GFP-GABARAP and the unlipidated G116A mutant, but not GFP-LC3B, the ULK1 LIR was required for maximal phosphorylation of ATG13-FLAG (Figure 7M and Figure S7F). Thus, GABARAP specifically promotes ULK1 activity through the ULK1 LIR motif and independently of lipidation.

In conclusion, we have shown a specific regulation of the centrosomal GABARAP pool by WAC and GM130 that correlates with initiation of autophagy, suggesting that centrosomally derived GABARAP signals back to autophagy initiation by sustaining ULK1 activity and explains why WAC, which inhibits GABARAP-GM130 interaction, also promotes ULK1 activation.

## Discussion

WAC and GM130 are two regulators of autophagy whose mechanism of action is unresolved and previously unconnected (McKnight et al., 2012, Totsukawa et al., 2011). Here we show that WAC directly binds to GM130 and that WAC-GM130 binding is required for autophagosome formation. WAC functions by suppressing GM130 binding to GABARAP, allowing the maintenance of a centrosomal GABARAP reservoir. Centrosomal GABARAP and Golgi WAC coordinate and regulate autophagosome formation through ULK1 activation.

Like GM130, WAC localizes to and co-purifies with the Golgi complex (Totsukawa et al., 2011), remaining on the Golgi after 1 M KCl wash (Nakamura et al., 1995, Totsukawa et al., 2011). We show that direct binding to GM130 tethers WAC to the Golgi complex and this is likely regulated by dephosphorylation of WAC during starvation. WAC-GM130 binding is independent of the RNF20/40 complex. The 10aa (aa611–620) of WAC are essential for GM130 binding and these 10aa are highly conserved back to *D. melanogaster* (Xu and Arnaout, 2002). WAC and GM130 form a stable complex: WAC remains bound to mitochondrially targeted GM130, even after 2 hr with LMB. GM130 has numerous functions, including vesicle tethering, signaling, and controlling mitosis (Baschieri et al., 2014, Nakamura, 2010, Wei et al., 2015).

GM130 can cycle between the ERGIC and the *cis*-Golgi (Marra et al., 2001), which explains the localization of GABARAP and WAC to these compartments. The binding of GM130 to GABARAP is direct and not regulated by starvation, and our live-cell imaging data suggest that the Golgi pool of GABARAP does not participate in autophagy. The strong preference of GM130 for GABARAP, and not LC3B for example, indicates a level of specificity that remains to be investigated.

In basal conditions, the Golgi acts as a hub for autophagy proteins such as BECLIN 1, GATE-16, ATG9, ATG16, GABARAP, and LC3 (Guo et al., 2012, Itoh et al., 2008, Kittler et al., 2001, Sagiv et al., 2000, Shoji-Kawata et al., 2013, Young et al., 2006), and Golgi-derived membranes are proposed to contribute to autophagosome biogenesis (Abada and Elazar, 2014). Recently, contact sites between the Golgi and the phagophore were identified that could be sites of lipid transfer (Biazik et al., 2015). Moreover, the Golgi complex is intimately linked with the centrosome, and GM130 controls centrosome morphology and function (Kodani et al., 2009, Kodani and Sütterlin, 2008). Golgi proteins such as AKAP450 (which binds GM130), GMAP-210, and HOOK3 connect the Golgi ribbon to the microtubule network and centrosome (Rios, 2014). Interestingly, ATG4D, which processes GABARAPL1, is localized to the centrosome (Betin and Lane, 2009). Here, we show an unexpected localization of non-lipidated GABARAP at the PCM.

We show that GM130 and WAC allow specific control of the centrosomal GABARAP pool. This pool of GABARAP is non-lipidated and how it associates with the PCM remains to be determined. Depletion of GM130 increases GABARAP residency on the centrosome and drives autophagy. In contrast, WAC knockdown results in loss of centrosomal GABARAP and relocalization to the ERGIC and Golgi due to increased GM130 binding. GABARAP directly binds microtubules (Wang and Olsen, 2000) and we speculate that microtubules may allow GABARAP transfer from the Golgi to the centrosome and from the centrosome to the autophagosome formation site. In the absence of microtubules, GABARAP relocalizes from the centrosome to Golgi ministacks, which are located at ER exit sites (Cole et al., 1996). However, the role of microtubules in these events remains to be clarified.

The Atg8s are critical autophagy proteins. In yeast there is one Atg8 and no GM130 or WAC homologs (Nakamura et al., 1997, Xu and Arnaout, 2002). In mammals there are 7 Atg8 homologs, the LC3s, and GABARAPs (Slobodkin and Elazar, 2013). Although there is evidence of both redundancy and specialization of the LC3 and GABARAP family members, a comprehensive understanding of regulation and function of these proteins is lacking. In addition, many LC3- and GABARAP-interacting proteins have been identified, raising questions on how these interactions are controlled (Birgisdottir et al., 2013).

Our work suggests that non-lipidated centrosomal GABARAP is transported to autophagosome formation sites where it localizes to autophagosomal membranes. Although the GABARAP subfamily promotes phagophore closure (Weidberg et al., 2010), it also binds the ULK complex. The ULK complex members ULK1/2, FIP200, and Atg13 bind GABARAP preferentially via LC3-interacting regions (LIRs) (Alemu et al., 2012, Kraft et al., 2012, Okazaki et al., 2000). The identification of LIRs in the ULK complex suggests that the LC3 and GABARAP subfamilies act as a scaffold for recruitment of the ULK complex to autophagic structures. This is supported by our data that the ULK1 LIR motif is required for ULK1 retention on GFP-DFCP1-positive membranes. Importantly, we show that knockdown of GABARAP specifically attenuates ULK1 activation: LC3B, GABARAPL1, and GATE-16 do not activate ULK1. In contrast, in yeast (Kraft et al., 2012) the Atg1 kinase is delivered to the vacuole via Atg8 to act as a brake on autophagy, and the Atg8-Atg1 interaction does not modulate kinase activity. The ULK1 association with GABARAP is constitutive and not regulated by starvation or lipidation, thus, the large non-lipidated pool of GABARAP at the centrosome may bind and prime ULK1 activity. In support of this, ULK1 activation by GABARAP, but not LC3B, requires the LIR motif but does not require lipidation. Thus, LIR motifs can be regulatory elements, for example, LIR-containing proteins displace Atg12–5-16 from Atg8–PE (Kaufmann et al., 2014). In the absence of WAC, aberrant GABARAP tethering to GM130 disrupts the autophagy-contributing centrosomal GABARAP reservoir and hence reduces GABARAP contribution to autophagosome formation and GABARAP-mediated ULK1 activation.

It has been hypothesized that the LIR-dependent binding of the ULK complex to LC3 and GABARAP proteins functions in the formation and maturation of autophagosomes, however the mechanism is unknown (Alemu et al., 2012). Supporting this idea, overexpression of an ULK1 LIR mutant results in the accumulation of WIPI2 puncta, suggesting autophagy has been stalled (Kraft et al., 2012). Similarly, knockdown of the GABARAP family causes accumulation of ATG5 and ATG16L1 puncta, suggesting autophagy is inhibited downstream of ATG12–5-16L1 complex recruitment (Weidberg et al., 2010).

Based on our data and existing literature, we propose a working model to explain the non-hierarchical (post-initiation) GABARAP-ULK1 function. Subsequent to activation of the BECLIN 1 complex by ULK1 (Russell et al., 2013), a pool of PtdIns(3)P is formed at the omegasome. This PtdIns(3)P recruits WIPI2, which recruits the ATG12–5-16L1 complex (Dooley et al., 2014). ATG16L1 can simultaneously bind WIPI2B and FIP200; FIP200 binding is downstream of WIPI2B and not required for LC3-lipidation (Dooley et al., 2014). Thus, recruitment of FIP200 (and the ULK complex) to the phagophore by ATG16L1 is downstream of PtdIns(3)P- (Karanasios et al., 2013), WIPI2B-, and ATG12–5-16L1-driven lipidation of the LC3 and GABARAP family (see Figure S7G). We hypothesize that the constitutive GABARAP-ULK1 complex, derived from either the centrosome or the cytosol, would be recruited to the phagophore through ATG16L1 binding. The GABARAP-ULK1 complex may also be stabilized by lipidation. GABARAP association maintains ULK1 activation and substrate phosphorylation during the final stages of phagophore formation, until the ULK1 complex dissociates (Karanasios et al., 2013) and the phagophore closes.

## Experimental Procedures

### Cell Culture and Reagents

Cell lines, transfection protocols, siRNA, DNA constructs, primers, and antibodies are detailed in the Supplemental Experimental Procedures.

### Microarray Studies

Whole-genome gene expression analysis was performed by the Genome Centre at Barts and the London School of Medicine and Dentistry, Queen Mary University of London (London, UK) using the Illumina human HT-12 v4 beadchip. Gene expression data were analyzed as described in the Supplemental Experimental Procedures.

### Protein Complex Purification and Mass Spectrometry

Protein complexes were pulled down from fed cells via a GFP tag using GFP-TRAP beads (ChromoTek) or by immunoprecipitation of WAC. Proteins were resolved by SDS-PAGE, fixed, and stained with GelCode, and full lanes were cut into slices for tryptic digestion and mass spectrometry analysis. For more details, see Supplemental Experimental Procedures.

## Accession Numbers

The accession number for the microarray data reported in this paper is GEO: GSE66475.

## Supplemental Information

Document S1. Supplemental Experimental Procedures and Figures S1–S7

Table S1. Overlapping Genes and Pathways Regulated by WAC, RNF20, and RNF40 Knockdown, Related to Figure 1

Movie S1. Centrosomal GABARAP Contributes to Autophagosome Formation, Related to Figure 6

Movie S2. GABARAP-Positive Autophagosomes Are Highly Mobile and Make Transient Interactions with the Centrosome, Related to Figure 6

Movie S3. Depletion of WAC Retains GABARAP on the Golgi, Where It Becomes Immobile and Does Not Make Autophagosomes, Related to Figure 7A

Document S2. Article plus Supplemental Information

## References

[bib1] Abada A., Elazar Z. (2014). Getting ready for building: signaling and autophagosome biogenesis. EMBO Rep..

[bib2] Alemu E.A., Lamark T., Torgersen K.M., Birgisdottir A.B., Larsen K.B., Jain A., Olsvik H., Øvervatn A., Kirkin V., Johansen T. (2012). ATG8 family proteins act as scaffolds for assembly of the ULK complex: sequence requirements for LC3-interacting region (LIR) motifs. J. Biol. Chem..

[bib3] Alers S., Löffler A.S., Wesselborg S., Stork B. (2012). Role of AMPK-mTOR-Ulk1/2 in the regulation of autophagy: cross talk, shortcuts, and feedbacks. Mol. Cell. Biol..

[bib4] Baschieri F., Confalonieri S., Bertalot G., Di Fiore P.P., Dietmaier W., Leist M., Crespo P., Macara I.G., Farhan H. (2014). Spatial control of Cdc42 signalling by a GM130-RasGRF complex regulates polarity and tumorigenesis. Nat. Commun..

[bib5] Behrends C., Sowa M.E., Gygi S.P., Harper J.W. (2010). Network organization of the human autophagy system. Nature.

[bib6] Betin V.M., Lane J.D. (2009). Caspase cleavage of Atg4D stimulates GABARAP-L1 processing and triggers mitochondrial targeting and apoptosis. J. Cell Sci..

[bib7] Biazik J., Ylä-Anttila P., Vihinen H., Jokitalo E., Eskelinen E.L. (2015). Ultrastructural relationship of the phagophore with surrounding organelles. Autophagy.

[bib8] Birgisdottir A.B., Lamark T., Johansen T. (2013). The LIR motif - crucial for selective autophagy. J. Cell Sci..

[bib9] Chan E.Y. (2009). mTORC1 phosphorylates the ULK1-mAtg13-FIP200 autophagy regulatory complex. Sci. Signal..

[bib10] Chang S.-H., Hong S.-H., Jiang H.-L., Minai-Tehrani A., Yu K.-N., Lee J.-H., Kim J.-E., Shin J.-Y., Kang B., Park S. (2012). GOLGA2/GM130, cis-Golgi matrix protein, is a novel target of anticancer gene therapy. Mol. Ther..

[bib11] Cole N.B., Sciaky N., Marotta A., Song J., Lippincott-Schwartz J. (1996). Golgi dispersal during microtubule disruption: regeneration of Golgi stacks at peripheral endoplasmic reticulum exit sites. Mol. Biol. Cell.

[bib12] Dooley H.C., Razi M., Polson H.E., Girardin S.E., Wilson M.I., Tooze S.A. (2014). WIPI2 links LC3 conjugation with PI3P, autophagosome formation, and pathogen clearance by recruiting Atg12-5-16L1. Mol. Cell.

[bib13] Füllgrabe J., Klionsky D.J., Joseph B. (2014). The return of the nucleus: transcriptional and epigenetic control of autophagy. Nat. Rev. Mol. Cell Biol..

[bib14] Guo Y., Chang C., Huang R., Liu B., Bao L., Liu W. (2012). AP1 is essential for generation of autophagosomes from the trans-Golgi network. J. Cell Sci..

[bib15] Itoh T., Fujita N., Kanno E., Yamamoto A., Yoshimori T., Fukuda M. (2008). Golgi-resident small GTPase Rab33B interacts with Atg16L and modulates autophagosome formation. Mol. Biol. Cell.

[bib16] Karanasios E., Stapleton E., Manifava M., Kaizuka T., Mizushima N., Walker S.A., Ktistakis N.T. (2013). Dynamic association of the ULK1 complex with omegasomes during autophagy induction. J. Cell Sci..

[bib17] Karpiuk O., Najafova Z., Kramer F., Hennion M., Galonska C., König A., Snaidero N., Vogel T., Shchebet A., Begus-Nahrmann Y. (2012). The histone H2B monoubiquitination regulatory pathway is required for differentiation of multipotent stem cells. Mol. Cell.

[bib18] Kaufmann A., Beier V., Franquelim H.G., Wollert T. (2014). Molecular mechanism of autophagic membrane-scaffold assembly and disassembly. Cell.

[bib19] Kimura S., Noda T., Yoshimori T. (2008). Dynein-dependent movement of autophagosomes mediates efficient encounters with lysosomes. Cell Struct. Funct..

[bib20] Kittler J.T., Rostaing P., Schiavo G., Fritschy J.M., Olsen R., Triller A., Moss S.J. (2001). The subcellular distribution of GABARAP and its ability to interact with NSF suggest a role for this protein in the intracellular transport of GABA(A) receptors. Mol. Cell. Neurosci..

[bib21] Kodani A., Sütterlin C. (2008). The Golgi protein GM130 regulates centrosome morphology and function. Mol. Biol. Cell.

[bib22] Kodani A., Kristensen I., Huang L., Sütterlin C. (2009). GM130-dependent control of Cdc42 activity at the Golgi regulates centrosome organization. Mol. Biol. Cell.

[bib23] Kraft C., Kijanska M., Kalie E., Siergiejuk E., Lee S.S., Semplicio G., Stoffel I., Brezovich A., Verma M., Hansmann I. (2012). Binding of the Atg1/ULK1 kinase to the ubiquitin-like protein Atg8 regulates autophagy. EMBO J..

[bib24] Lamb C.A., Yoshimori T., Tooze S.A. (2013). The autophagosome: origins unknown, biogenesis complex. Nat. Rev. Mol. Cell Biol..

[bib25] Marra P., Maffucci T., Daniele T., Tullio G.D., Ikehara Y., Chan E.K., Luini A., Beznoussenko G., Mironov A., De Matteis M.A. (2001). The GM130 and GRASP65 Golgi proteins cycle through and define a subdomain of the intermediate compartment. Nat. Cell Biol..

[bib26] McKnight N.C., Jefferies H.B., Alemu E.A., Saunders R.E., Howell M., Johansen T., Tooze S.A. (2012). Genome-wide siRNA screen reveals amino acid starvation-induced autophagy requires SCOC and WAC. EMBO J..

[bib27] Middendorp S., Küntziger T., Abraham Y., Holmes S., Bordes N., Paintrand M., Paoletti A., Bornens M. (2000). A role for centrin 3 in centrosome reproduction. J. Cell Biol..

[bib28] Nakamura N. (2010). Emerging new roles of GM130, a cis-Golgi matrix protein, in higher order cell functions. J. Pharmacol. Sci..

[bib29] Nakamura N., Rabouille C., Watson R., Nilsson T., Hui N., Slusarewicz P., Kreis T.E., Warren G. (1995). Characterization of a cis-Golgi matrix protein, GM130. J. Cell Biol..

[bib30] Nakamura N., Lowe M., Levine T.P., Rabouille C., Warren G. (1997). The vesicle docking protein p115 binds GM130, a cis-Golgi matrix protein, in a mitotically regulated manner. Cell.

[bib31] Oakley B.R. (2000). An abundance of tubulins. Trends Cell Biol..

[bib32] Okazaki N., Yan J., Yuasa S., Ueno T., Kominami E., Masuho Y., Koga H., Muramatsu M. (2000). Interaction of the Unc-51-like kinase and microtubule-associated protein light chain 3 related proteins in the brain: possible role of vesicular transport in axonal elongation. Brain Res. Mol. Brain Res..

[bib33] Orsi A., Razi M., Dooley H.C., Robinson D., Weston A.E., Collinson L.M., Tooze S.A. (2012). Dynamic and transient interactions of Atg9 with autophagosomes, but not membrane integration, are required for autophagy. Mol. Biol. Cell.

[bib34] Pankiv S., Lamark T., Bruun J.A., Øvervatn A., Bjørkøy G., Johansen T. (2010). Nucleocytoplasmic shuttling of p62/SQSTM1 and its role in recruitment of nuclear polyubiquitinated proteins to promyelocytic leukemia bodies. J. Biol. Chem..

[bib35] Poser I., Sarov M., Hutchins J.R., Hériché J.K., Toyoda Y., Pozniakovsky A., Weigl D., Nitzsche A., Hegemann B., Bird A.W. (2008). BAC TransgeneOmics: a high-throughput method for exploration of protein function in mammals. Nat. Methods.

[bib36] Rios R.M. (2014). The centrosome-Golgi apparatus nexus. Philos. Trans. R. Soc. Lond. B Biol. Sci..

[bib37] Russell R.C., Tian Y., Yuan H., Park H.W., Chang Y.-Y., Kim J., Kim H., Neufeld T.P., Dillin A., Guan K.-L. (2013). ULK1 induces autophagy by phosphorylating Beclin-1 and activating VPS34 lipid kinase. Nat. Cell Biol..

[bib38] Sagiv Y., Legesse-Miller A., Porat A., Elazar Z. (2000). GATE-16, a membrane transport modulator, interacts with NSF and the Golgi v-SNARE GOS-28. EMBO J..

[bib39] Shema E., Tirosh I., Aylon Y., Huang J., Ye C., Moskovits N., Raver-Shapira N., Minsky N., Pirngruber J., Tarcic G. (2008). The histone H2B-specific ubiquitin ligase RNF20/hBRE1 acts as a putative tumor suppressor through selective regulation of gene expression. Genes Dev..

[bib40] Shoji-Kawata S., Sumpter R., Leveno M., Campbell G.R., Zou Z., Kinch L., Wilkins A.D., Sun Q., Pallauf K., MacDuff D. (2013). Identification of a candidate therapeutic autophagy-inducing peptide. Nature.

[bib41] Shpilka T., Weidberg H., Pietrokovski S., Elazar Z. (2011). Atg8: an autophagy-related ubiquitin-like protein family. Genome Biol..

[bib42] Slobodkin M.R., Elazar Z. (2013). The Atg8 family: multifunctional ubiquitin-like key regulators of autophagy. Essays Biochem..

[bib43] Stolz A., Ernst A., Dikic I. (2014). Cargo recognition and trafficking in selective autophagy. Nat. Cell Biol..

[bib44] Totsukawa G., Kaneko Y., Uchiyama K., Toh H., Tamura K., Kondo H. (2011). VCIP135 deubiquitinase and its binding protein, WAC, in p97ATPase-mediated membrane fusion. EMBO J..

[bib45] Wang H., Olsen R.W. (2000). Binding of the GABA(A) receptor-associated protein (GABARAP) to microtubules and microfilaments suggests involvement of the cytoskeleton in GABARAPGABA(A) receptor interaction. J. Neurochem..

[bib46] Wei J.-H., Zhang Z.C., Wynn R.M., Seemann J. (2015). GM130 Regulates Golgi-Derived Spindle Assembly by Activating TPX2 and Capturing Microtubules. Cell.

[bib47] Weidberg H., Shvets E., Shpilka T., Shimron F., Shinder V., Elazar Z. (2010). LC3 and GATE-16/GABARAP subfamilies are both essential yet act differently in autophagosome biogenesis. EMBO J..

[bib48] Wiedenmann J., Ivanchenko S., Oswald F., Schmitt F., Röcker C., Salih A., Spindler K.D., Nienhaus G.U. (2004). EosFP, a fluorescent marker protein with UV-inducible green-to-red fluorescence conversion. Proc. Natl. Acad. Sci. USA.

[bib49] Wong M., Munro S. (2014). Membrane trafficking. The specificity of vesicle traffic to the Golgi is encoded in the golgin coiled-coil proteins. Science.

[bib50] Xu G.M., Arnaout M.A. (2002). WAC, a novel WW domain-containing adapter with a coiled-coil region, is colocalized with splicing factor SC35. Genomics.

[bib51] Young A.R.J., Chan E.Y.W., Hu X.W., Köchl R., Crawshaw S.G., High S., Hailey D.W., Lippincott-Schwartz J., Tooze S.A. (2006). Starvation and ULK1-dependent cycling of mammalian Atg9 between the TGN and endosomes. J. Cell Sci..

[bib52] Zhang F., Yu X. (2011). WAC, a functional partner of RNF20/40, regulates histone H2B ubiquitination and gene transcription. Mol. Cell.

