# Supplementary material for: Activation of ULK Kinase and Autophagy by GABARAP Trafficking from the Centrosome Is Regulated by WAC and GM130
Source: Mol Cell. 2015 Dec 17;60(6):899–913. doi: 10.1016/j.molcel.2015.11.018 (PMC4691241; doi:10.1016/j.molcel.2015.11.018)
Supplement: Document S1. Supplemental Experimental Procedures and Figures S1–S7 [file mmc1.pdf]

Molecular Cell, Volume *60*

Supplemental Information

Activation of ULK Kinase and Autophagy by GABARAP Trafficking from the Centrosome Is Regulated by WAC and GM130

Justin Joachim, Harold B.J. Jefferies, Minoo Razi, David Frith, Ambrosius P. Snijders, Probir Chakravarty, Delphine Judith, Sharon A. Tooze

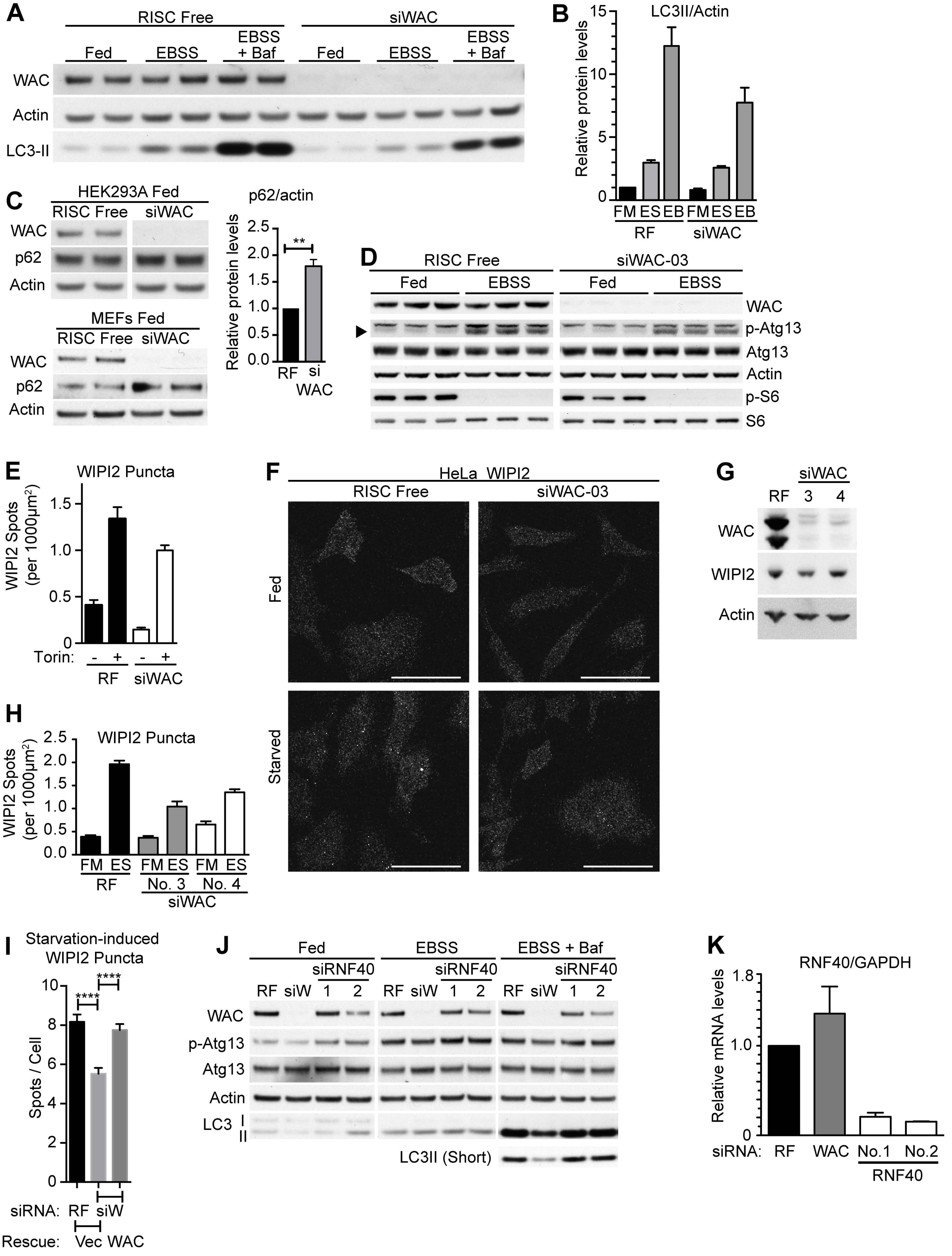

Supplementary Figure S1 Joachim et al.

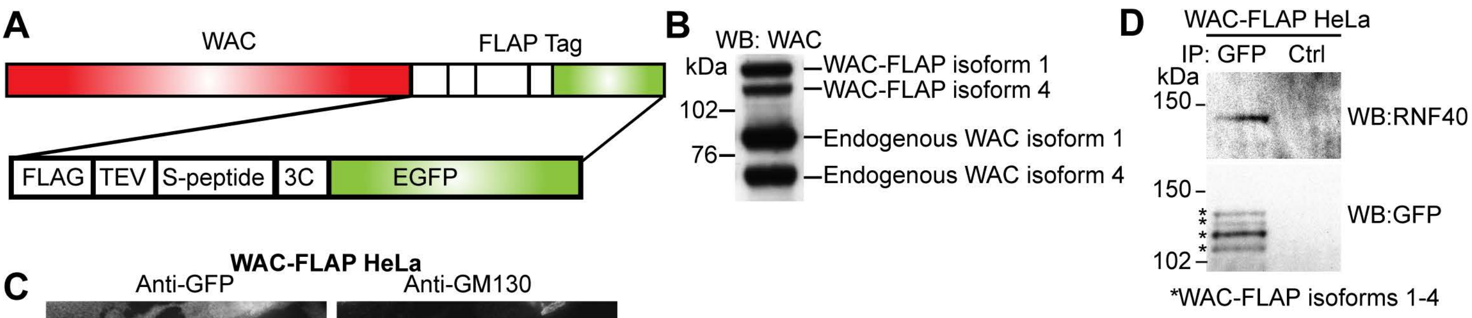

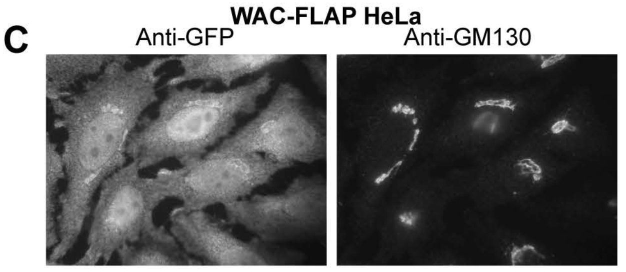

E

Gene names	PEP	Intensity	Unique peptides Ctrl	Unique peptides IP WAC	Unique peptides Ctrl	Unique peptides IP WAC-FLAP	Crapome Num of Expt. (found/total)
RNF20	0	1.05E+12	0	65	0	52	27 / 411
RNF40	0	9.78E+11	0	35	0	33	19 / 411
WAC (Q9BTA9)	0	3.51E+11	0	23	0	19	14 / 411
ERC1	5.30E-116	1.11E+08	0	21	0	1	44 / 411
GOLGA2	4.68E-28	9.31E+08	0	6	0	3	26 / 411
ADD2	1.89E-28	2.04E+10	0	5	0	1	12 / 411
PRPF40A	8.18E-09	8.03E+07	0	2	0	1	76 / 411
CYTSA;SPECC1L	9.85E-210	4.73E+10	0	1	0	20	17 / 411
WTAP	2.52E-214	1.94E+10	0	1	0	7	41 / 411
RBM15	6.52E-57	1.80E+10	0	1	0	9	40 / 411
UBE2A	2.29E-09	1.65E+10	0	1	0	1	
PSRC1	0.0014697	6.91E+09	0	1	0	2	
RAB1A;RAB1B;RAB1C	1.99E-08	6.39E+09	0	1	0	2	69 / 411
MYO18A	3.99E-34	5.42E+09	0	1	0	7	11 / 411
MCM4	4.76E-05	8.24E+08	0	1	0	2	113 / 411
EGFR;ERBB4;ERBB2	4.41E-05	7.47E+08	0	1	0	1	16 / 411
THOC2	1.96E-10	1.38E+08	0	1	0	1	64 / 411
MAP7D1;RPRC1	1.30E-07	1.38E+07	0	1	0	1	24 / 411
NACA;hCG_2016482	5.59E-07	2.65E+05	0	1	0	1	125 / 411

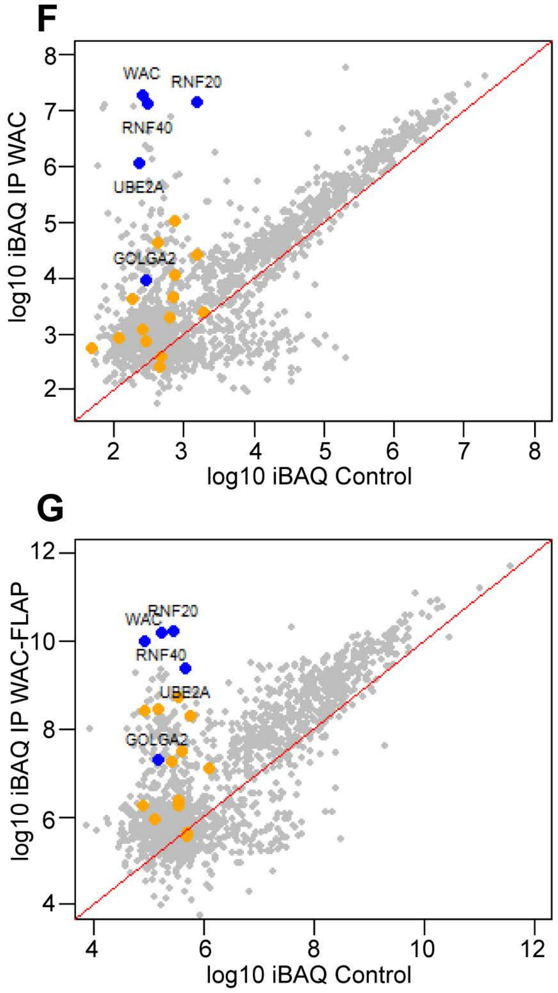

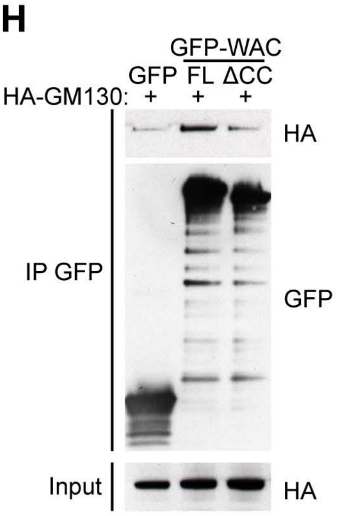

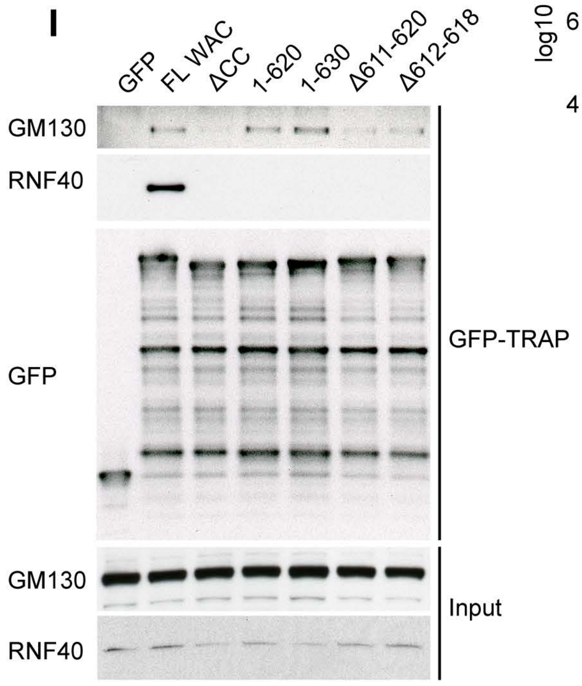

Supplementary Figure S2 Joachim et al.

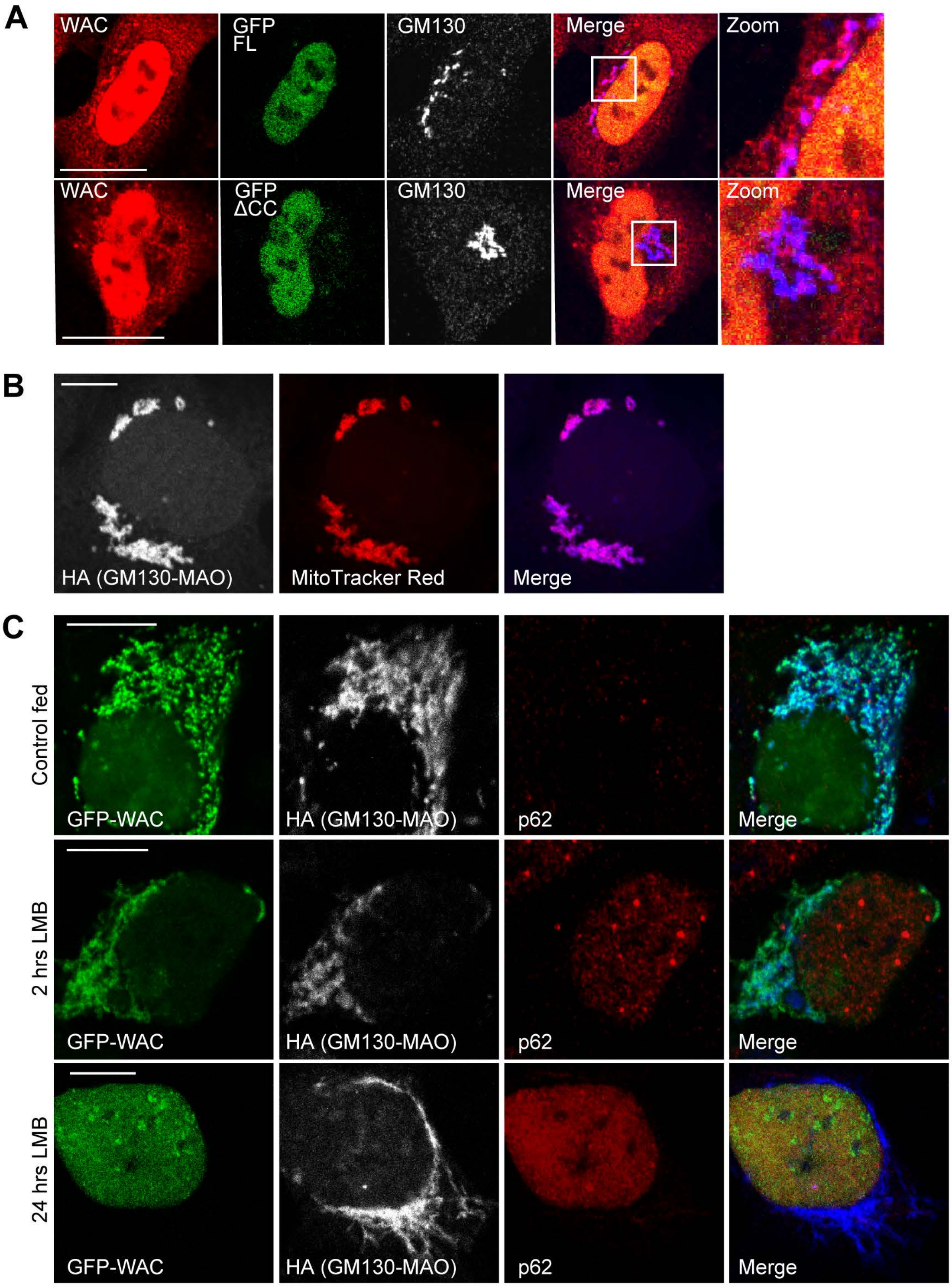

Supplementary Figure S3 Joachim et al.

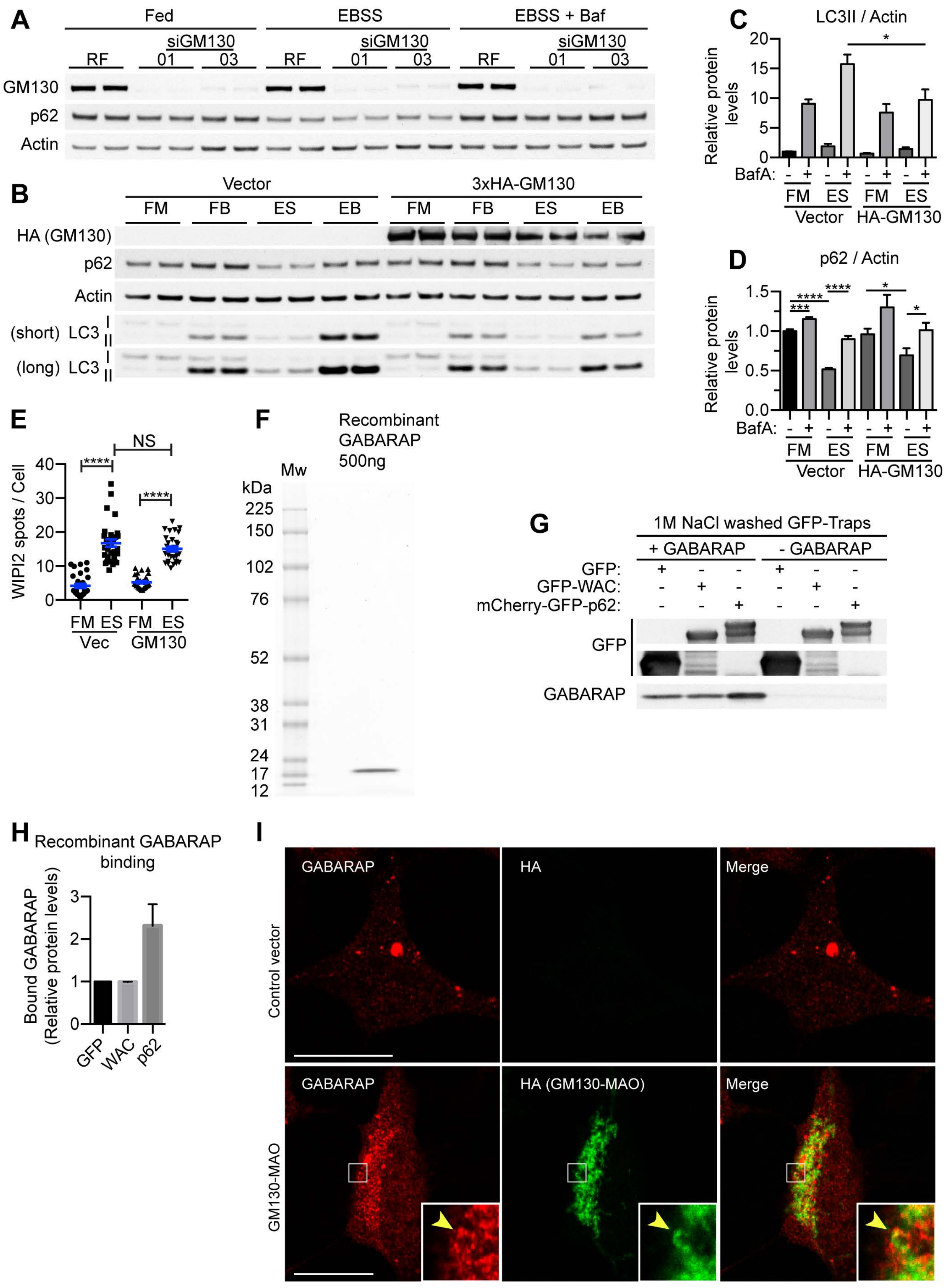

Supplementary Figure S4 Joachim et al.

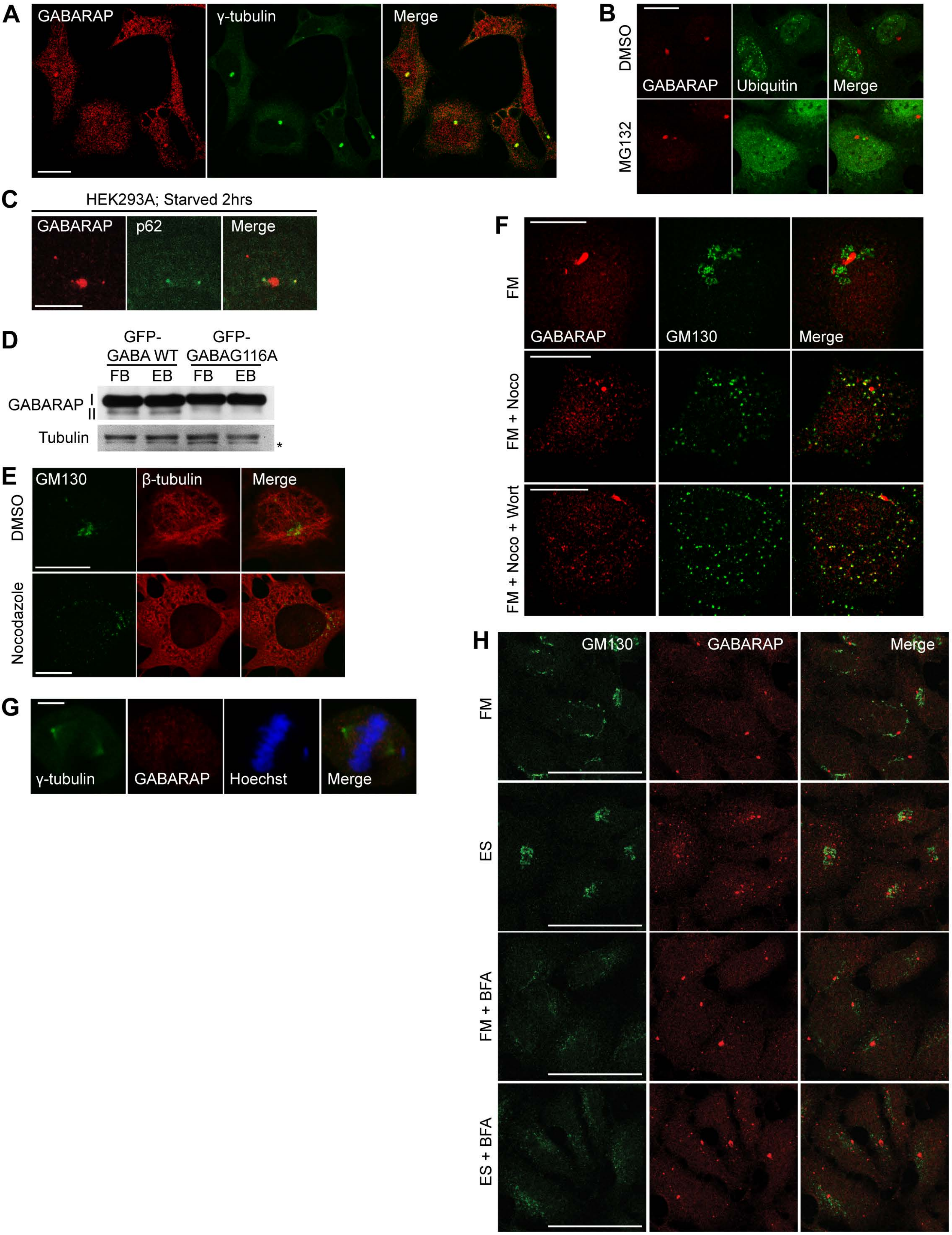

Supplementary Figure S5 Joachim et al.

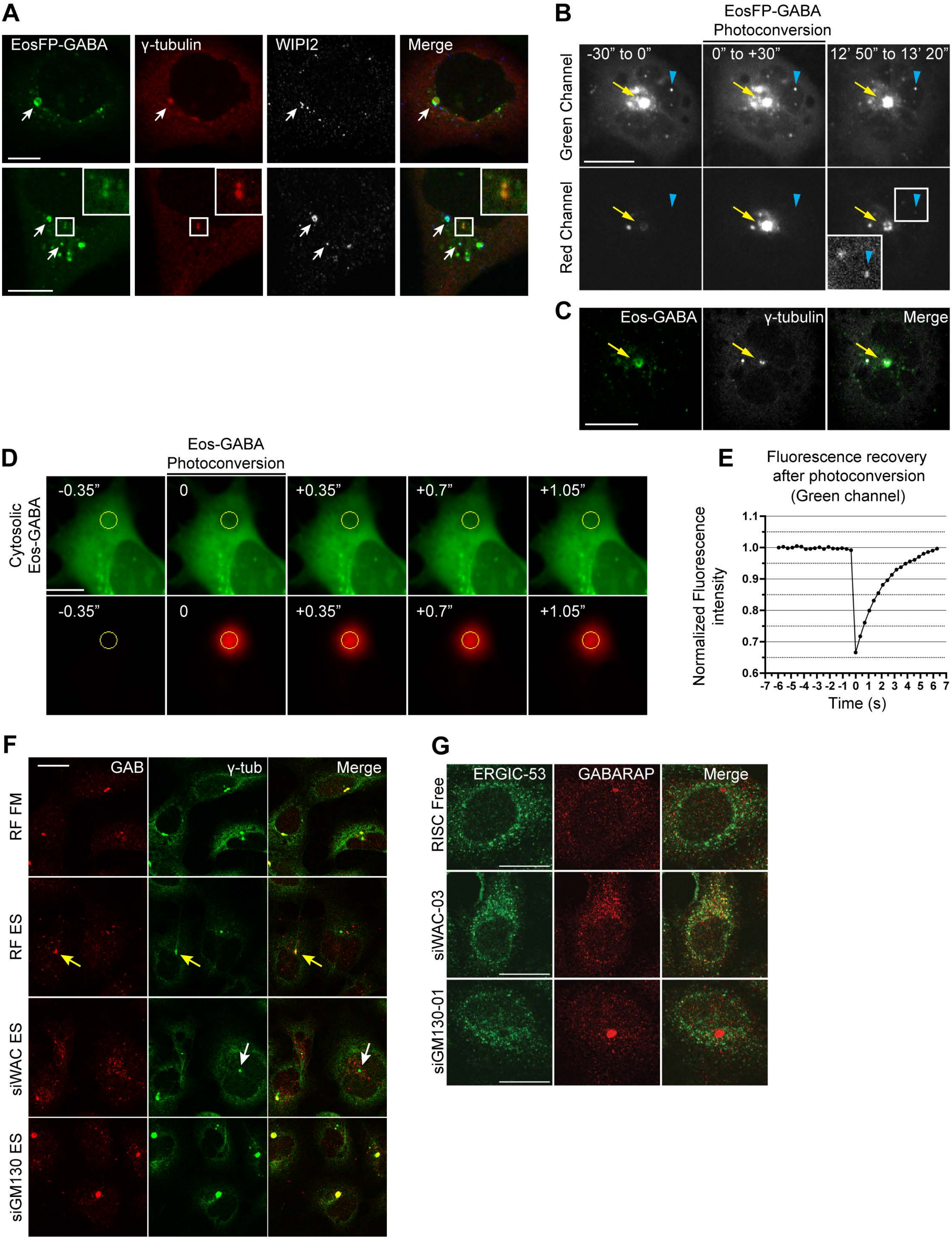

Supplementary Figure S6 Joachim et al.

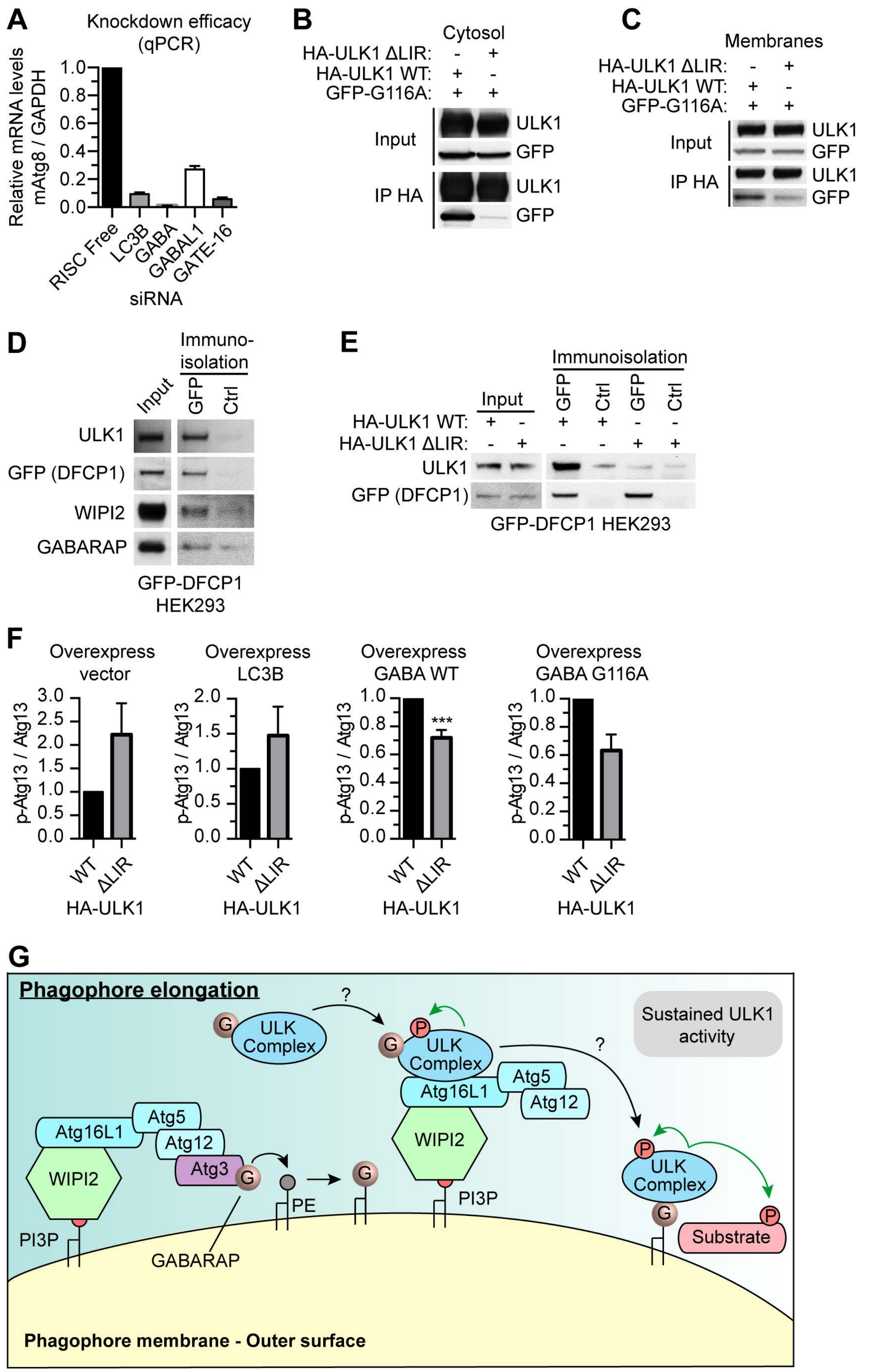

Supplementary Figure S7 Joachim et al.

Supplemental Figure Legends

Figure S1. WAC promotes autophagy in multiple cell lines and independently of RNF40, Related to Figure 1

A) HEK293A cells were treated for 72 hr with RF or WAC siRNA and then incubated in full medium (Fed), EBSS or EBSS + BafA1 for 2 hr before immunoblot analysis. B) Quantification of (A), mean ± SEM from 2 independent experiments. Full medium, FM; EBSS, ES; EBSS + BafA1, EB. C) HEK293A or MEF cells were treated for 72 hr with RF or WAC siRNA before immunoblot analysis. Graph shows quantification from HEK293A cells, statistics were performed using an unpaired Student's t test, *, $p \leq 0.05$. Mean ± SEM from 2 independent experiments. D) HEK293A cells were treated for 72 hr with RF or WAC siRNA and then incubated in full medium (Fed) or EBSS for 2 hr before immunoblot analysis. p-Atg13, anti-Atg13 pSer318; p-S6, anti-S6 pSer240/244. E) HEK293A cells were treated for 72 hr with RF or WAC siRNA and then incubated in full medium or full medium + Torin1 before analysis by confocal microscopy and quantification of WIPI2 puncta. Mean ± SEM from at least 80 cells per condition. F) HeLa cells were treated for 72 hr with RF or WAC siRNA (03 or 04) and then incubated in full medium (Fed) or EBSS (Starved) prior to analysis by either confocal microscopy (F) or immunoblot (G, Fed). H) Quantification of (F), mean ± SEM from at least 120 cells per condition. Full medium, FM; EBSS, ES. I) Vector (Vec) or Myc-WAC was expressed in HEK293A cells that were treated with RF or WAC (siW) siRNA for 72 hr and starved in EBSS for 2 hr prior to WIPI2 staining and confocal microscopy. Statistics were performed using an unpaired Student's t test, *, $p \leq 0.05$. Mean ± SEM from 3 independent experiments, >300 cells were counted per condition. J) HEK293A cells treated with RF, WAC or 2 different RNF40 siRNAs for 72 hr were incubated in full medium (fed), EBSS or EBSS + BafA1 for 2 hr prior to immunoblot analysis. K) HEK293A cells treated with RF, WAC or 2 different RNF40 siRNAs for 72 hr were maintained in full medium before analysis by qRT-PCR. mRNA levels of RNF40 were normalized to GAPDH. Mean ± SEM from duplicates. Excised lanes are indicated by a gap and remaining lanes are from the same gel.

Figure S2. Use of a bacterial artificial chromosome (BAC) to generate a WAC-FLAP HeLa cell line that mimics endogenous WAC and identifies GM130 as a WAC interactor, Related to Figure 2

A) Schematic showing WAC BAC containing the WAC gene (red) fused to a C-terminal FLAP tag. B) Immunoblot of WAC-FLAP HeLa cells showing tagged WAC isoform 1 and 4. C) Epifluorescence microscopy of WAC-FLAP HeLa cells. D) Lysates from WAC-FLAP HeLa cells were used for GFP-Trap pull-downs followed by immunoblot. Blocked agarose beads plus lysate was used as a negative control. E) MaxQuant protein groups table after applying filter criteria (see Supplemental Experimental Procedures). WAC and known interactors are highlighted in blue. GM130 (GOLGA2) is highlighted in orange. PEP = posterior error probability for protein identification. Control = beads and lysate without antibody. As a further quality parameter Crapome frequencies were added in the "Crapome Num of Expt. (found/total)" column, http://www.crapome.org. F) Intensity based absolute quantification (iBAQ) values are plotted for WAC interactors after immunoprecipitation of endogenous WAC from HEK293A cells versus the control sample (beads and lysate). Proteins in table (E) are highlighted as blue or orange dots. G) Intensity based absolute quantification (iBAQ) values are plotted for WAC interactors after immunoprecipitation of WAC-FLAP from HeLa cells versus the control sample (beads and lysate). Proteins in table (E) are highlighted as blue or orange dots. H) Lysates from HEK293A cells co-transfected with 3xHA-GM130 and EGFP, EGFP-WAC (FL) or aa1-610 (ΔCC) were used for GFP-Trap pull-down followed by immunoblot. I) Lysates from HEK293A cells transiently expressing EGFP, EGFP-WAC FL, ΔCC, 1-620, 1-630, Δ611-620, or Δ612-618 were used for GFP-Trap pull-down followed by immunoblot analysis.

Figure S3. Nuclear export of WAC is required to maintain cytoplasmic WAC-GM130 binding, Related to Figure 3

A) siRNA resistant EGFP-WAC FL or ΔCC were expressed in HEK293A cells treated with WAC siRNA for 72 hr before analysis by confocal microscopy using the indicated antibodies. Scale bars, 20 μm. B) HEK293A cells expressing GM130-ΔCterm-HA-MAO were incubated in full medium with MitoTracker Red for 2 hr before staining with anti-HA antibody and analysis by confocal microscopy. Scale bars, 10 μm. C) HEK293A cells expressing EGFP-WAC and GM130-ΔCterm-HA-MAO were incubated in full medium without or with Leptomycin B (LMB) for the indicated time before staining with HA and p62 antibodies and analysis by confocal microscopy. Scale bars, 10 μm.

Figure S4. GM130 overexpression suppresses LC3 lipidation during starvation and recruits GABARAP, Related to Figure 4

A) HEK293A cells treated with RF, GM130-01 or -03 siRNAs for 72 hr were incubated with full medium (fed), EBSS or EBSS + BafA for 2 hrs followed by immunoblot. The quantification of this experiment is shown in Fig. 4F. B) HEK293A cells expressing empty vector or 3xHA-GM130 were incubated in full medium (FM), full medium with BafA (FB), EBSS (ES), or EBSS with BafA (EB) for 2 hr prior to immunoblot. C) Quantification of (B) statistics were performed using an unpaired Student's t test, *, $p \leq 0.05$. Mean ± SEM from 3 independent experiments. D) Quantification of (B) statistics were performed using an unpaired Student's t test, *, $p \leq 0.05$. Mean ± SEM from 5 independent experiments. E) HEK293A cells expressing empty vector or 3xHA-GM130 were incubated in full medium (FM) or EBSS (ES) for 2 hr prior to confocal microscopy. F) Colloidal Coomassie stained SDS-PAGE gel showing purified untagged human GABARAP from *E. coli*. G) Lysates from HEK293A cells expressing the indicated GFP constructs were used for GFP-TRAP followed by salt washes and incubation with purified recombinant GABARAP from (F) and immunoblot. mCherry-GFP-p62 was used as a positive control for GABARAP binding. H) Quantification of (G) from 2 independent experiments. I) Vector or GM130-ΔCterm-HA-MAO were expressed in HEK293A cells followed by 2 hr incubation with EBSS, anti-HA and anti-GABARAP labelling and analysis by confocal microscopy. Arrows indicate co-localization. Scale bars, 20 μm.

Figure S5. Centrosomal GABARAP localization is dynamic and regulated by the cell cycle and microtubules but does not require the secretory pathway, Related to Figure 5

A) HEK293A cells were stained with sheep anti-GABARAP antibody and mouse anti-γ-tubulin before analysis by confocal microscopy. Scale bars, 20 μm. B) HEK293A cells incubated in DMSO or MG132 for 2 hr or C) in EBSS for 2 hr and assessed using the indicated antibodies. Scale bars, 10 μm. D) HEK293A cells expressing EGFP-GABARAP wild-type or the G116A mutant were incubated in full medium with BafA (FB) or EBSS with BafA (EB) for 2 hr prior to electrophoresis on an 8% SDS-PAGE gel and immunoblot. *, non-specific band. E) HEK293A cells were incubated in full medium with DMSO or nocodazole for 5 hr and assessed by confocal microscopy using the indicated antibodies. Scale bars, 20 μm. F) HEK293A cells were incubated in full medium (FM), FM with nocodazole or FM with nocodazole and wortmannin for 5 hr and assessed by confocal

microscopy using the indicated antibodies. Scale bars, 20 µm. G) Asynchronous HEK293A cells were incubated in full medium and metaphase cells were analysed by confocal microscopy. A single mitotic cell is shown. Scale bars, 5 µm. H) HEK293A cells were incubated in full medium (FM) or EBSS (ES) with or without Brefeldin A (BFA) for 2 hr and assessed by confocal microscopy using the indicated antibodies. Scale bars, 50 µm.

Figure S6. EosFP-GABARAP localizes to autophagosomes and the centrosome. Cytoplasmic EosFP-GABARAP translocates much more rapidly than the centrosomal species, Related to Figure 6

A) HEK293A cells expressing EosFP-GABARAP were incubated in EBSS for 2 hr before labelling with γ-tubulin and WIPI2 and analysis by confocal microscopy. Scale Bars, 10 µm. B) Live HEK293A cells expressing EosFP-GABARAP were starved in EBSS and imaged every 5 s using a swept field confocal microscope. Photoconversion (PC) was performed with localized pulses of 405nm light. PC moment is set to 0 s. Yellow arrow indicates photoconverted region. Blue arrow shows defined punctum. To reduce noise, 6 sequential images, equal to 30 s timeframe, were averaged for each time period shown. Scale bar, 20 µm. Inset has been contrast adjusted for clarity. C) Confocal microscopy performed on cell from (B). After time-lapse imaging, cells were fixed and stained for γ-tubulin. Yellow arrow indicates the same structure as in (B). Scale bar, 20 µm. D) HEK293A cells expressing high levels of cytosolic EosFP-GABARAP were starved in EBSS and imaged every 0.35 s using a swept field confocal microscope. Photoconversion (PC) was performed with 405nm light. PC moment is set to 0 s. Scale bar, 10 µm. E) Graph shows quantification of fluorescence intensity from yellow circle in images (D), for green channel only. Intensity 6 s prior to PA moment is set to 1 for normalization. F) HEK293A cells were treated with RF, WAC or GM130 siRNAs for 72 hr, incubated with full medium (FM) or EBSS (ES) for 2 hr and analyzed by confocal microscopy. Scale bars, 20 µm. Yellow arrows show GABARAP on the centrosome, white arrows show GABARAP dissociation from the centrosome. G) HEK293A cells were treated with RF, WAC or GM130 siRNAs for 72 hr, incubated with EBSS for 2 hr and analyzed by confocal microscopy. Scale bars, 20 µm.

Figure S7. GABARAP drives LIR-dependent ULK1 activation independently of lipidation and is present on DFCP1 positive membranes, Related to Figure 7

A) HEK293A cells were treated with RF, LC3B, GABARAP, GABARAPL1 or GATE-16 siRNA for 72 hr prior to analysis by qRT-PCR. Expression of each transcript is normalised to RF. n=3 independent experiments that are matched with 3 experiments from Fig. 7H. B) HEK293A cells expressing the indicated constructs were incubated in EBSS for 2 hr and subjected to subcellular fractionation and the cytosol was used for immunoprecipitation followed by immunoblot. C) HEK293A cells expressing the indicated constructs were incubated in EBSS for 2 hr and subjected to subcellular fractionation and the membranes were used for immunoprecipitation followed by immunoblot. D) HEK293 cells stably expressing GFP-DFCP1 were incubated in EBSS for 2 hr before homogenization and immunoisolation of the GFP-DFCP1 compartment with anti-GFP or anti-FLAG M2 control and immunoblot. E) HEK293 cells stably expressing GFP-DFCP1 and the indicated ULK1 constructs were incubated in EBSS for 2 hr before homogenization and immunoisolation of the GFP-DFCP1 compartment with anti-GFP or anti-FLAG M2 control and immunoblot. F) Quantifications of Fig. 7M, overexpress vector, 4 experiments; overexpress LC3B, 3 experiments; overexpress GABARAP, 5 experiments; overexpress G116A, 2 experiments. Statistics were performed using an unpaired Student's t test, ***, $p \leq 0.001$. G) Model is explained in the discussion. GABARAP promotes ULK1 activity and this requires the LIR motif of ULK1. As GABARAP acts during the membrane expansion/closure stage, this could maintain ULK1 activation past the early initiation stage of autophagy.

Table S1. Overlapping genes and pathways regulated by WAC, RNF20 and RNF40 knockdown, Related to Figure 1

Movie S1. Centrosomal GABARAP contributes to autophagosome formation, Related to Figure 6

Live cell imaging of a HEK293A cell expressing EosFP-GABARAP. Cells were washed into EBSS, imaged and photoconverted at 37°C with 10% CO_2 using a Nikon Eclipse Ti Swept Field Confocal microscope. Arrows mark the structures shown in Fig. 6A. Yellow arrow shows photoconverted centrosomal GABARAP. Blue arrow shows GABARAP punctum acquiring centrosomal GABARAP. Movie spans 25 mins played at 5 fps with 1 frame equal to 30 s. Note: first 5 frames are equal to 5 x 1 s images before photoactivation.

Movie S2. GABARAP positive autophagosomes are highly mobile and make transient interactions with the centrosome, Related to Figure 6

Live cell imaging of a HEK293A cell expressing EosFP-GABARAP. Cells were washed into EBSS, imaged and photoconverted at 37°C with 10% CO_2 using a Nikon Eclipse Ti Swept Field Confocal microscope. Centrosomal EosFP-GABARAP in the center of the cell is targeted for photoconversion. Blue arrow shows GABARAP punctum making contact with centrosomal GABARAP before leaving in a different direction. Movie spans 26 mins played at 10 fps with 1 frame equal to 5 s. Note: first 12 frames span the 60 s before photoactivation.

Movie S3. Depletion of WAC retains GABARAP on the Golgi where it becomes immobile and does not make autophagosomes, Related to Figure 7A

Live cell imaging of HEK293A cells depleted of WAC expressing EosFP-GABARAP. Cells were washed into EBSS, imaged and photoconverted at 37°C with 10% CO_2 using a Nikon Eclipse Ti Swept Field Confocal microscope. Arrows mark the structures shown in Fig. 7E. Golgi-localized GABARAP (yellow arrows) was targeted for photoconversion. Movie spans 25 mins played at 11.5 fps with 1 frame equal to 13.1 s. Maximum intensity projections of z-stacks are shown.

Extended Experimental Procedures

Cell culture and reagents

HEK293A, U2OS, RPE-1, HCT116, MEF, HEK293 GFP-DFCP1 and HeLa cells and their derivatives were grown in full medium: DMEM supplemented with 10% fetal calf serum and 4 mM L-glutamine. To induce autophagy, cells were washed 3 times with Earle's balanced salt solution (EBSS) and incubated in EBSS for two hours, unless otherwise stated. Where indicated, cells were treated with: 100 nM Bafilomycin A1 (Calbiochem), 100 nM Wortmannin (Calbiochem), 10 µg/ml Brefeldin A (Sigma), 100 nM Torin1 (Cayman Chemical), 50 µM Nocodazole (Sigma) or 20 ng/ml Leptomycin B (Sigma) for the specified time. HEK293 Flp-In T-Rex GFP-GABARAP cells were maintained in full medium + 200 µg/ml Hygromycin B + 5 µg/ml Blasticidin and induced for 24 hr with 1 µg/ml tetracycline in full medium to express GFP-GABARAP. HEK293 Flp-In T-Rex GFP-GABARAP cells were a kind gift from Anne Simonsen, (University of Oslo, Norway). The HEK293 GFP-DFCP1 stably expressing cells were a gift from N. Ktistakis (clone 201) (Axe et al., 2008) and maintained in the presence of G418 at µg/ml.

HeLa cell line stably expressing WAC-FLAP was established by transient transfection with a BAC (CTD-2309I17) containing the human WAC gene. The WAC BAC was modified by homologous recombination to contain a C-terminal FLAP tag (Poser et al., 2008). Transfection was carried out with Effectene (Qiagen) according to the method in (Poser et al., 2008). After transfection cells were selected with G418 (800 µg/ml) and cloned by FACs sorting for EGFP expression before being maintained in the presence of G418 at 400 µg/ml.

Lipofectamine 2000 (Life Technologies) was used for transient transfection of HEK293A cells according to the manufacturer's instructions. For rescue of autophagy with GFP-WAC and analysis by immunoblot, transfection was carried out with Lipofectamine 3000 (Life Technologies) according to the manufacturer's instructions. DNA plasmids were used at a concentration of 1 µg/mL of transfection mix. Where indicated pcDNA 3.1 (+) was used as an empty vector control.

For RNAi of HEK293A, MEF and HeLa cells, cells were transfected with the relevant siRNA oligo using Lipofectamine 2000 (Life Technologies). Cells were harvested 72 hr after transfection. For RNAi of GABARAP, cells were transfected with siRNA on day 1 and also day 2 of the procedure. Final concentration of siRNA oligos was 37.5 nM. siRNA oligos used (Dharmacon): D-001220-01 (RISC-Free, control), D-013325-02 (WAC-02 for MEFs), D-013325-03 (WAC-03), D-013325-04 (WAC-04), D-017282-01 (GM130-01), D-017282-03 (GM130-03), D-006913-01 (RNF40-01), D-006913-02 (RNF40-02), D-007027-01 (RNF20-01), D-007027-02 (RNF20-02), D-007027-03 (RNF20-03), D-007027-04 (RNF20-04), D-005049-04 (ULK1), D-012368-02 (GABARAP-02), D-012368-03 (GABARAP-03), D-012846-01 (LC3B), D-014715-02 (GABARAPL1), D-006853-03 (GATE-16). Unless otherwise specified, the siRNAs used for WAC, GM130 and GABARAP were -03, -01 and -02 respectively.

Knockdown and rescue experiments for Immunofluorescence were performed by transfection with siRNA on day 1 and transfection with DNA on day 3, as specified above. Rescue assay was carried out 24 hr after DNA transfection.

Human WAC BAC CTD-2309I17 was purchased from Life Technologies, pSC101-BAD-gbaAtet (for Red/ET recombination) was purchased from gene bridges, R6Kamp-FLAP for tagging the WAC BAC was a gift from Tony Hyman (Max Planck Institute or Molecular Cell Biology and Genetics, Dresden, Germany). Human Myc-WAC (pcDNA 3.1 (+)) and EGFP-WAC (pEGFP C2) truncations and Strep-Tag II-GM130 (Rat) (pcDNA 3.1 (+)) and EosFP-GABARAP (human) (pcDNA 3.1 (+)) were generated by PCR and cloned by ligation or using the In-fusion HD cloning kit (Clontech). Point mutations were generated by using QuikChange Multi Site-Directed Mutagenesis Kit (Agilent Technologies). Deletions were generated by Inverse PCR or PCR SOEing. HA-ULK1 and HA-ULK1 ΔLIR (D356A, F357A, P361A) were used in (Kraft et al., 2012). HA-ULK1 ΔLIR/KI contains the additional K49I kinase-inactivating mutation of human ULK1. Atg13-FLAG was used in (Chan et al., 2009). 3xHA-GM130 (pcDNA 3.1 (+)) (Rat) was a gift from Joachim Seemann (UT Southwestern Medical Center, Dallas, US), HA-GM130 truncations (Human) were a gift from Angelika Barnekow (University

of Münster, Germany), Rat GM130-ΔCterm-HA-MAO was a gift from Sean Munro (MRC laboratory of molecular biology, Cambridge, UK). EGFP-mAtg8 homologues and Myc-GABARAP (human) were a gift from Terje Johansen (UiT, The Arctic University of Norway, Tromsø). HA-VP35 (pcDNA 3.1/myc-His(-)) (Gantke et al., 2013) was a gift from Jesper Svejstrup (Francis Crick Institute, London, UK).

Mouse monoclonal antibodies: anti-ubiquitin (MBL, D058-3) anti-Centrin3 (Abcam, ab54531), anti-γ-tubulin ascites (Sigma, GTU-88, T6557), anti-p62 (BD Biosciences, 610832 & Abnova, H00008878-M01), anti-Myc (CRUK, 9E10), anti-FLAG M2 (Sigma), anti-p230/GOLGA4 (BD Biosciences, 611280), anti-ERGIC-53 (Enzo Life Sciences, ALX-804-602-C100), anti-βCOP (Sigma, M3A5, G2279), anti-GFP (CRUK, 3E1 & Roche, 11814460001), anti-GM130 (for IF) (BD Biosciences, 610822). Rabbit polyclonal antibodies: anti-β-tubulin (Abcam, ab6046), anti-GABARAP (Abgent, AP1821a), anti-HA (Covance, PRB-101P), anti-GFP (santa cruz, sc-8334), anti-RNF40 (Bethyl Laboratories, A300-718A), anti-WAC for WB (McKnight et al., 2012) for IF (Totsukawa et al., 2011), anti-WIPI2 (Polson et al., 2010), anti-Actin (Abcam, ab8227), anti-LC3 (Abcam, ab48394), anti-ULK1 (Santa Cruz, sc-33182), anti-ULK1 pSer757 (Cell Signaling, 6888), anti-Atg13 (Chan et al., 2009), anti-Atg13 pSer318 (Rockland, 600-401-C49), anti-phospho-S6 Ser240/244 (Cell Signaling, 2215). Rabbit monoclonal: anti-GM130 (Abcam, ab52649), anti-S6 (Cell Signaling, 2217). Hamster polyclonal: anti-Atg9 (Young et al., 2006). Sheep: anti-TGN46 (Serotec, AHP500G). Rat: anti-HA (for IF) (Roche, 3F10, 11867423001). Guinea pig polyclonal anti-p62 (for IF) (Progen, GP62-C). Antibodies were used at manufacturer's suggested concentrations. Secondary antibodies for IF, from Life Technologies unless otherwise specified, were anti-rabbit IgG Alexa Fluor 488, 555 and 647, anti-mouse IgG Alexa Fluor 488, 647 and 350, anti-guinea pig Alexa Fluor 555 and FITC (Santa Cruz), anti-rat Alexa Fluor 633 and 488, anti-sheep Alexa Fluor 488 and 647 and anti-hamster Cy3 (Jackson ImmunoResearch). HRP-conjugated secondary antibodies used for WB were from GE Healthcare.

Western Blotting

Cells were lysed in ice-cold TNTE buffer (20 mM Tris, pH 7.4, 150 mM NaCl, 0.5% w/v Triton X-100, 5 mM EDTA) containing EDTA-free Complete Protease Inhibitor cocktail (Roche). Lysates were cleared by centrifugation and resolved on NuPAGE®Bis-Tris 4–12% gels (Life Technologies) followed by transfer onto a PVDF membrane (Millipore). For WAC phosphorylation analysis, 25 μM Phos-Tag (NARD institute, AAL-107) 7.5% SDS-PAGE gels were prepared according to manufacturer's instructions. Following incubation with primary and secondary antibodies the blots were developed by enhanced chemiluminescence (GE Healthcare). Densitometry was performed with ImageJ software. For western blotting of GM130, primary antibody was diluted with SignalBoost Immunoreaction Enhancer Kit (Merck Millipore, 407207) and blots were developed with Luminata Crescendo Western HRP substrate (Merck Millipore).

Microarray studies

HEK293A cells were treated with RISC free, WAC, RNF40 or RNF20 siRNA pools (WAC-03 and 04; RNF40-01 and 02; RNF20-01, 02, 03 and 04) in triplicate and maintained in full medium, as described above. RNA was extracted using the RNeasy kit (Qiagen). RNA labelling was performed using the Ambion Total Prep Kit (Life Technologies).

Gene expression data was analyzed using Bioconductor 2.2 (http://bioconductor.org) running on R2.7.1 (http://www.R-project.org) Normalized probe set expression measures were calculated using log2 transformation and quantile normalization using the Lumi package (Du et al., 2008). All groups contained three independent samples except for RNF40, which had two independent samples after removal of one replicate for quality control.

To determine significant differences of expression in the three groups: siWAC, siRNF20 and siRNF40 relative to RISC free, a moderated Student's t-test was computed on a gene-by-gene basis using the empirical Bayes statistics in the Limma package (Ritchie et al., 2015). The resultant p-values were adjusted for multiple testing using the False Discovery Rate (FDR) Benjamini and Hochberg method, where any probe sets that exhibited a FDR

of less than 0.05 were called differentially expressed. No fold change thresholds were applied.

Differential genes from three comparisons were intersected to identify common genes (Fig. 1G and Table S1). The common genes (319) were further subdivided into two groups: those whose FC difference relative to RISC free were down regulated in all comparisons (183) and those whose FC difference relative to RISC free were up regulated in all comparisons (118). 18 genes were differentially regulated between knockdown of the 3 proteins and subtracted from our analysis.

Differentially expressed genes (301) were analyzed for enrichment of pathways, biological processes and transcription factor targets using gene sets from Metacore Pathway analysis tool (Thomson Reuters) using a hypergeometric distribution to determine enriched gene set using all genes on the Illumina array as the background. Pathways or processes that showed a FDR of less than 0.05 were called as enriched.

qRT-PCR

HEK293A cells were treated with the indicated siRNA and maintained in full medium. Total RNA was isolated using the RNeasy kit (Qiagen) and cDNA synthesis was performed with SuperScript II reverse transcriptase (Life Technologies). qRT-PCR was performed using the Fast SYBR Green Master Mix (Applied Biosystems, 4385612) and PCR products were detected by the 7500 FAST Real-Time PCR System (Applied Biosystems). The following primers were used to generate amplicons: forward (*5'-AAC AAC GGC AGG CTT GTG AAG ATG-3'*) and reverse (*5'-ATC GGA GAA GGG CTT CCA CAG TTT-3'*) primers for RNF40, forward (*5'-GAC CAC TTT GTC AAG CTC ATT TC-3'*) and reverse (*5'-CTC TCT TCC TCT TGT GCT CTT G-3'*) primers for GAPDH, forward (*5'- GCG AGA AGA TCC GAA AGA AA -3'*) and reverse (*5'- GAT CAG AAG GCA CCA GGT ATT T -3'*) primers for GABARAP, forward (*5'- TGG GCC AAC TGT ATG AGG A -3'*) and reverse (*5'- CTA CCC CCA AGT CCA GGT G -3'*) primers for GABARAPL1, forward (*5'- CCG TCG TTG TTG TTG TGC T -3'*) and reverse (*5'- CTC CAC GCA TCT GTG TTC C -3'*) primers for GATE-16, forward (*5'- GAG GAT CTT TAG GCC TGA G -3'*) and reverse (*5'- TTC TCA CAC AGC CCG TTT AC -3'*) primers for LC3B. The CT

values corresponding to target mRNA was normalized to that of GAPDH mRNA.

Protein complex purification and mass spectrometry

HEK293A or HeLa WAC-FLAP cells, maintained in full medium, were washed in PBS and lysed in TNTE buffer (20 mM Tris-HCl pH 7.4, 150 mM NaCl, 5 mM EDTA, 0.5 % Triton X-100, 1x Complete protease inhibitor (Roche), 1x PhosSTOP (Roche)) and the lysate clarified by centrifugation (16,100 x g, 15 min). Lysates from HeLa WAC-FLAP cells were incubated with GFP-TRAP® beads at 4°C for 2 hr. Lysates from HEK293A cells were incubated with Rabbit anti-WAC bound to protein G sepharose (Sigma) at 4°C for 2 hr. Pelleted beads were washed 3 times with TNTE buffer and eluted with 2x Laemmli sample buffer at 100°C for 10 min.

Eluted proteins were separated by SDS-PAGE and 8 bands covering the entire lane were excised for each sample. In-gel trypsin digestion was performed using a Perkin Elmer Janus liquid handling system. Lyophilized peptide samples were dissolved in 15 µl of 0.1 % TFA and subjected to LC-MS analysis using a LTQ-Orbitrap instrument for data acquisition. Raw spectra were processed using the MaxQuant/Andromeda bioinformatics suite (Cox and Mann, 2008) and further analyzed in Perseus. Data was searched against a UniProt fasta database containing human sequences and intensity based absolute quantification (iBAQ) (Schwanhausser et al., 2011) was used for label free quantification. WAC, WAC-FLAP and control IPs (beads and lysate) were analyzed to screen for novel WAC interactors and filtered as follows. Only proteins that received iBAQ values in both experiments and not in the control IPs were considered candidates. The original dataset contained 1536 protein identifications and using the filter criteria the list was reduced to 20 candidates that were enriched in the WAC & WAC-FLAP pull-downs vs control conditions.

Immunoprecipitation

Cells were lysed using TNTE buffer (20 mM Tris-HCl pH 7.4, 150 mM NaCl, 5 mM EDTA, 0.5 % Triton X-100, 1x Complete protease inhibitor (Roche), 1x PhosSTOP (Roche)) and the clarified lysates used for

immunoprecipitation with either rabbit anti-WAC, rabbit anti-GABARAP or rat anti-HA for 2 hr at 4°C. Where indicated, species matched anti-GFP is used as a control IP. Antibodies were coupled to protein G sepharose (Sigma). Pelleted beads were washed 3 times with TNTE buffer and eluted with 2x Laemmli sample buffer at 100°C for 10 min before resolving by SDS-PAGE (4-12 % Bis-Tris NuPAGE gels, Life Technologies) and western blotting. GFP-tagged proteins were immunoprecipitated using GFP-TRAP® beads and HA-tagged proteins with anti-HA affinity matrix 3F10 (Roche), using the same buffer and protocol. During western blotting of IP experiments, TrueBlot® (Rockland) was used to reduce background from IgG. Where indicated immunoprecipitates where washed 5 x 1M NaCl TNTE buffer (20 mM Tris-HCl pH 7.4, 1M NaCl, 5 mM EDTA, 0.5 % Triton X-100, 1x Complete protease inhibitor (Roche)) and then washed 2 x TNTE buffer before addition of cell lysate. For dephosphorylation of GFP-WAC, washed GFP-TRAPs of GFP-WAC were incubated in 100 µL volume with 1 µL lambda phosphatase (NEB, P0753) with the accompanying manufacturer's buffer and 1mM MnCl2 at 30°C for 30 mins. As a control, samples were incubated with TNTE buffer instead. Lambda phosphatase was then removed by 2 x TNTE buffer washes and where indicated HEK293A cell lysates were incubated with GFP-TRAPs before washing and immunoblotting as above.

Immunoisolation of GFP-DFCP1-positive membranes

HEK293 cells stably expressing GFP-DFCP1 were treated with EBSS for 2 hr. Cells were then washed in ice cold PBS and harvested by centrifugation at 200 x g at 4°C. Pellets were resuspended using a cold isotonic buffer (20mM HEPES, pH 7.4; 250mM sucrose; 1mM EDTA) supplemented with EDTA-free Complete protease inhibitor cocktail (Roche). The resuspended pellet was then passed through a 27G needle for homogenization before clarification by centrifugation at 3000 x g at 4°C. Supernatants were used for incubation overnight at 4°C with mouse anti-FLAG M2 or mouse anti-GFP antibody protein A Dynabeads®. The GFP-DFCP1-positive membranes on the beads were then washed 3 times (Isotonic buffer supplemented with 75mM NaCl) and eluted with 2x laemmli sample buffer before resolving by SDS-PAGE and western blotting.

Where indicated, GFP-DFCP1 cells were transfected with the appropriated plasmid 24h prior isolation of GFP-DFCP1-positive membrane.

Immunoprecipitation from GFP-DFCP1-positive membranes was performed by solubilizing immunoisolated membrane using cold TNTE buffer (20 mM Tris-HCl pH 7.4, 150 mM NaCl, 5 mM EDTA, 0.5 % Triton X-100, 1x Complete protease inhibitor (Roche), 1x PhosSTOP (Roche)). The lysates then were used for immunoprecipitation with the indicated antibody protein A Dynabeads® for 2h at 4°C. Subsequently, beads were washed 3 times with TNTE buffer and eluted with laemmli sample buffer before resolving by SDS-PAGE and western blotting.

Subcellular fractionation

Cells were washed and pelleted in ice cold HEPES buffer (20 mM HEPES-KOH pH 7.5, 10 mM KCl, 2.5 mM MgOAc, 1mM EDTA) and then resuspended in HEPES buffer with sucrose (20 mM HEPES-KOH pH 7.5, 250 mM sucrose, 10 mM KCl, 2.5 mM MgOAc, 1mM EDTA, 1 mM DTT, 1X PhosSTOP (Roche), 1X Complete EDTA-free protease inhibitor cocktail (Roche)) and incubated on ice for 20 minutes. Cells were then homogenised by passing through a 27G needle and homogenization was monitored by Trypan Blue staining. Nuclei were removed by centrifugation at 5,000 rpm for 5 minutes at 4°C in an Eppendorf micro centrifuge, this was repeated and the supernatant was subjected to centrifugation at 112,500 x g for 1 hr at 4°C to obtain the membrane pellet and the cytosol supernatant.

1% Triton X-100 and 150 mM NaCl were added to the cytosol fractions which were then subjected to immunoprecipitation at 4°C with GFP-TRAP beads (Chromotek) or anti-HA affinity matrix clone 3F10 (Roche) followed by SDS-PAGE and western blotting. Immunoprecipitates were washed 3 x with ice cold TNTE buffer before analysis (20 mM Tris-HCl pH 7.4, 150 mM NaCl, 5 mM EDTA, 0.5 % Triton X-100, 1x Complete protease inhibitor (Roche), 1x PhosSTOP (Roche)).

For membrane pellets, an equivalent volume (to cytosol) of HEPES buffer with sucrose (with 1% Triton X-100 and 150 mM NaCl) was added, and the pellets were incubated at 37°C for 30 minutes to solubilise membranes that would be insoluble in Triton X-100 at 4°C. Any insoluble material was

cleared by centrifugation at 16,100 x g and the supernatants were subjected to immunoprecipitation at 37°C followed by SDS-PAGE and western blotting. Immunoprecipitates were washed 3 x with TNTE buffer warmed to 37°C before analysis.

Protein expression, purification, *in vitro* binding and competition

Human GST-WAC fusion proteins were cloned into pGEX-4T-2 and expressed in *E. coli* BL21-CodonPlus(DE3)-RIL (Agilent) cells in LB medium. Human GST-GABARAP pGEX-5X-1 was a gift from Zvulun Elazar (Weizmann Institute of Science, Israel). Expression was induced by addition of 1 mM IPTG at OD_{600} = 0.6 and cells were incubated at 37°C for 4 hr. Harvested cells were lysed using sonication on ice in a lysis buffer (PBSA + 1 % Triton X-100, supplemented with 1 x Complete protease inhibitor (Roche)) and the clarified supernatant was subsequently applied to Glutathione Sepharose 4B beads (GE Healthcare). After several washes with (PBSA + 1 % Triton X-100 + 500 mM NaCl supplemented with 1 x Complete protease inhibitor (Roche)), fusion protein-bound beads were used directly in GST pulldown assays. Purified untagged human GABARAP was a gift from Stephane Mouilleron, Francis Crick Institute, London, UK.

To produce recombinant untagged WAC aa320-647, the protein was expressed as above and bound to Glutathione Sepharose 4B beads. The immobilized protein was incubated with Thrombin protease (GE Healthcare) for 16 hr at room temperature in PBS to cleave off the GST tag and 2X Complete EDTA-free protease inhibitor cocktail (Roche) was added to the supernatant followed by analysis by SDS-PAGE and colloidal Coomassie staining.

Strep-Tag II-GM130 (Rat) was cloned into pcDNA 3.1(+) and co-expressed in HEK293A cells with HA-VP35 to boost protein expression (Gantke et al., 2013). Cells were lysed in TNTE buffer (20 mM Tris-HCl pH 7.4, 150 mM NaCl, 5 mM EDTA, 0.5 % Triton X-100, 1x Complete protease inhibitor (Roche), 1x PhosSTOP (Roche)). After clarification of lysates, lysates were incubated with magnetic Strep-Tactin beads (Qiagen) for 1 hr before 5x washes in stringent conditions (20 mM Tris-HCl pH 7.4, 1 M NaCl, 5 mM EDTA, 1 % Triton X-100, 1x Complete protease inhibitor (Roche), 1x

PhosSTOP (Roche)). Beads were incubated with elution buffer (TNTE + 10mM Biotin) for 15 mins on ice to elute Strep-Tag II-GM130. Purity of eluted protein was assessed by SDS-PAGE and colloidal coomassie staining.

For *in vitro* binding, soluble Strep-Tag II-GM130 was incubated with immobilized GST (20 μg), GST-GABARAP (20 μg), GST-WAC or GST-WAC ΔCC on glutathione beads for 2 hr at 4°C in TNTE buffer (GST-WAC) or 1.5 hr at 4°C in TNTE buffer supplemented with 10% (v/v) glycerol and 0.1% (w/v) BSA (GST and GST-GABARAP). Beads were then washed 3 x with TNTE before SDS-PAGE and western blotting. For competition, soluble Strep-Tag II-GM130 was incubated with recombinant WAC aa320-647 for 2 hr at 4°C in TNTE buffer supplemented with 10% (v/v) glycerol and 0.1% (w/v) BSA and this mixture was then incubated with GST or GST-GABARAP beads as above.

Confocal and epifluorescence microscopy

Cells were grown on coverslips, fixed with 3 % paraformaldehyde in PBS for 20 min before permeabilization with either 0.1 % saponin in PBS for 20 min (WAC & βCOP), 0.2 % Triton X-100 in PBS for 3 min (ERGIC-53) or room temperature methanol for 5 min (WIPI2 & GABARAP & other antibodies). Coverslips were then blocked in 5 % BSA in PBS (Roche) after methanol permeabilization, with 0.2 % gelatin in PBS after Triton X-100 permeabilization or with 0.1 % saponin + 1 % BSA + 0.2 % gelatin in PBS for 20 min. Coverslips were incubated with primary antibody in 1 % BSA in PBS or 0.2 % gelatin in PBS for 1 hr at room temperature. For WAC and βCOP staining, coverslips were incubated with primary antibody + 0.1 % saponin, 1 % BSA and 0.2 % gelatin in PBS overnight at 4°C. Coverslips were washed and incubated with secondary antibody in the same buffer as primary for 1 hr, before final washing with PBS and water. LC3 and WIPI2 puncta formation and centrosomal GABARAP intensity was quantified by Imaris image analysis software.

Live cell imaging

Live cell imaging was performed on HEK293A cells transiently expressing EosFP-GABARAP. Alternatively, cells were treated with WAC

siRNA for 48 hr prior to transfection with EosFP-GABARAP and imaging 24 hr later. Cells were washed 3 times into EBSS and maintained at 37°C with 10% CO_2 during imaging with a Nikon Eclipse Ti microscope. Photoconversion was performed with pulses of 405 nm light. Images were processed, and data analyzed, using the Fiji distribution of ImageJ. Where indicated, cells were pretreated with 50 µM Nocodazole (Sigma) in full medium for 2 hr before incubation with EBSS + 50 µM Nocodazole followed by imaging.

After time-lapse microscopy, cells were immediately fixed in 3% paraformaldehyde and processed for confocal microscopy as detailed above. Photoconverted cells were located using gridded MatTek dishes. During 4-colour imaging (Fig. 6C), 405 nm illumination was carried out only after acquisition of the other fluorophore signals, in order to prevent artefactual photoconversion.

Statistical analysis

Statistics were performed using GraphPad Prism 6 software, as detailed in the figure legends.

Primers used in this study

Primer	Description	Sequence (5’-3’)
WAC SDM Primer 1	Silent mutations To make WAC (Q9BTA9-1) resistant to WAC-03 siRNA	GAA AAG AAT CTA CAT CAG GTG ATA AAC CCG TAT CAC ATT C
WAC SDM Primer 2	Silent mutations To make WAC (Q9BTA9-1) resistant to WAC-03 siRNA	CTA CAT CAG GTG ATA AGC CGG TAT CGC ATT CTT GCA CAA CTC CTT C
WAC SDM I626S L629S	Mutations of hydrophobic ‘a’ and ‘d’ positions in WAC CC domain to remove RNF40 binding	TTG CGA GAG CAA AGG AGC CTA TTT TCG AGA CAA CAA ATT AAG
GABARAP SDM primer	To make human GABARAP G116A mutation	GAC GAA AGT GTC TAC GCT CTG TGA AGC TGC TCG

References

Axe, E.L., Walker, S.A., Manifava, M., Chandra, P., Roderick, H.L., Habermann, A., Griffiths, G., and Ktistakis, N.T. (2008). Autophagosome formation from membrane compartments enriched in phosphatidylinositol 3-phosphate and dynamically connected to the endoplasmic reticulum. J Cell Biol *182*, 685-701.

Chan, E.Y., Longatti, A., McKnight, N.C., and Tooze, S.A. (2009). Kinase-inactivated ULK proteins inhibit autophagy via their conserved C-terminal domain using an Atg13-independent mechanism. Mol Cell Biol *29*, 157-171.

Cox, J., and Mann, M. (2008). MaxQuant enables high peptide identification rates, individualized p.p.b.-range mass accuracies and proteome-wide protein quantification. Nat Biotechnol *26*, 1367-1372.

Du, P., Kibbe, W.A., and Lin, S.M. (2008). lumi: a pipeline for processing Illumina microarray. Bioinformatics *24*, 1547-1548.

Gantke, T., Boussouf, S., Janzen, J., Morrice, N.A., Howell, S., Muhlberger, E., and Ley, S.C. (2013). Ebola virus VP35 induces high-level production of recombinant TPL-2-ABIN-2-NF-kappaB1 p105 complex in co-transfected HEK-293 cells. Biochem J *452*, 359-365.

Kraft, C., Kijanska, M., Kalie, E., Siergiejuk, E., Lee, S.S., Semplicio, G., Stoffel, I., Brezovich, A., Verma, M., Hansmann, I.*, et al.* (2012). Binding of the Atg1/ULK1 kinase to the ubiquitin-like protein Atg8 regulates autophagy. EMBO J *31*, 3691-3703.

McKnight, N.C., Jefferies, H.B., Alemu, E.A., Saunders, R.E., Howell, M., Johansen, T., and Tooze, S.A. (2012). Genome-wide siRNA screen reveals amino acid starvation-induced autophagy requires SCOC and WAC. EMBO J *31*, 1931-1946.

Polson, H.E.J., de Lartigue, J., Rigden, D.J., Reedijk, M., Urbe, S., Clague, M.J., and Tooze, S.A. (2010). Mammalian Atg18 (WIPI2) localizes to omegasome-anchored phagophores and positively regulates LC3 lipidation. Autophagy *6*, 506-522.

Poser, I., Sarov, M., Hutchins, J.R., Heriche, J.K., Toyoda, Y., Pozniakovsky, A., Weigl, D., Nitzsche, A., Hegemann, B., Bird, A.W.*, et al.* (2008). BAC TransgeneOmics: a high-throughput method for exploration of protein function in mammals. Nat Methods *5*, 409-415.

Ritchie, M.E., Phipson, B., Wu, D., Hu, Y., Law, C.W., Shi, W., and Smyth, G.K. (2015). limma powers differential expression analyses for RNA-sequencing and microarray studies. Nucleic Acids Res *43*, e47.

Schwanhausser, B., Busse, D., Li, N., Dittmar, G., Schuchhardt, J., Wolf, J., Chen, W., and Selbach, M. (2011). Global quantification of mammalian gene expression control. Nature *473*, 337-342.

Totsukawa, G., Kaneko, Y., Uchiyama, K., Toh, H., Tamura, K., and Kondo, H. (2011). VCIP135 deubiquitinase and its binding protein, WAC, in p97ATPase-mediated membrane fusion. Embo J *30*, 3581-3593.

Young, A.R.J., Chan, E.Y.W., Hu, X.W., Köchl, R., Crawshaw, S.G., High, S., Hailey, D.W., Lippincott-Schwartz, J., and Tooze, S.A. (2006). Starvation and ULK1-dependent cycling of Mammalian Atg9 between the TGN and endosomes. J Cell Sci *119*, 3888-3900.
