# Supplementary figures and images for: Activation of ULK Kinase and Autophagy by GABARAP Trafficking from the Centrosome Is Regulated by WAC and GM130
Source: Mol Cell. 2015 Dec 17;60(6):899–913. doi: 10.1016/j.molcel.2015.11.018 (PMC4691241; doi:10.1016/j.molcel.2015.11.018)

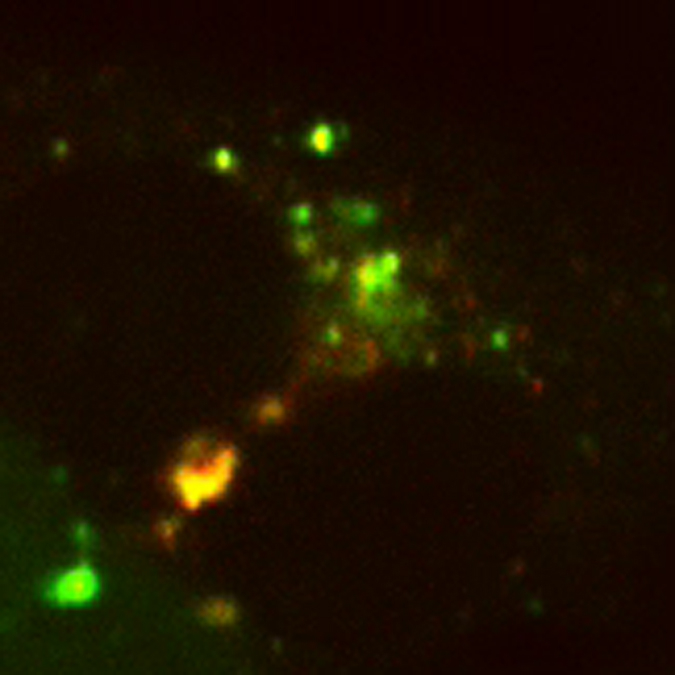

Supplement: Movie S1. Centrosomal GABARAP Contributes to Autophagosome Formation, Related to Figure 6 [file mmc3.jpg]

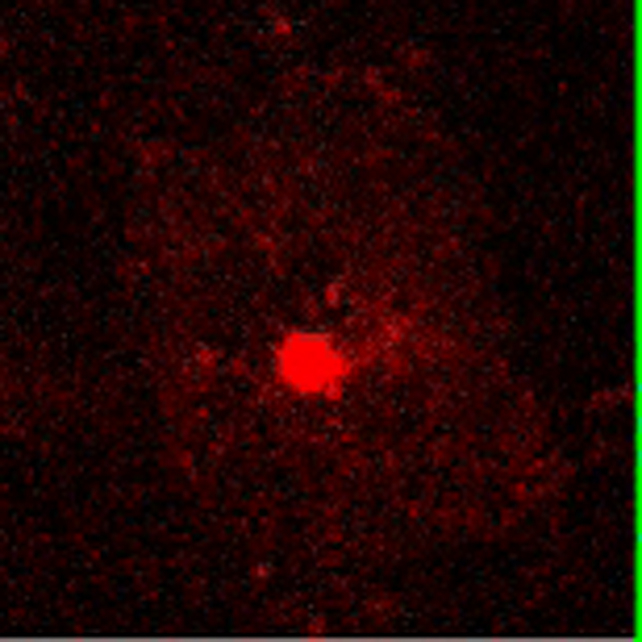

Supplement: Movie S2. GABARAP-Positive Autophagosomes Are Highly Mobile and Make Transient Interactions with the Centrosome, Related to Figure 6 [file mmc4.jpg]

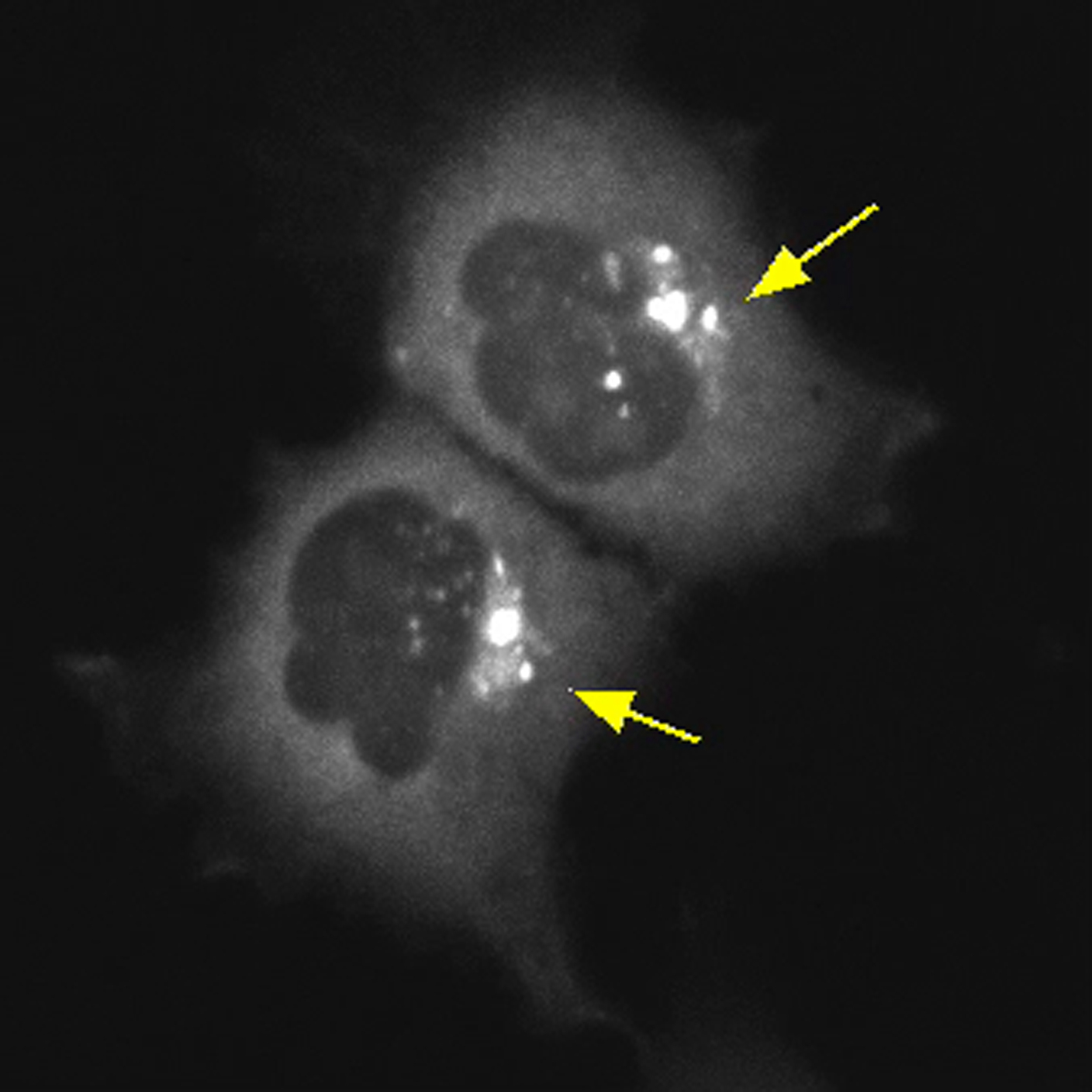

Supplement: Movie S3. Depletion of WAC Retains GABARAP on the Golgi, Where It Becomes Immobile and Does Not Make Autophagosomes, Related to Figure 7A [file mmc5.jpg]
