# Supplementary material for: Activation of ULK Kinase and Autophagy by GABARAP Trafficking from the Centrosome Is Regulated by WAC and GM130
Source: Mol Cell. 2015 Dec 17;60(6):899–913. doi: 10.1016/j.molcel.2015.11.018 (PMC4691241; doi:10.1016/j.molcel.2015.11.018)
Supplement: Document S2. Article plus Supplemental Information [file mmc6.pdf]

Molecular Cell

Article

Activation of ULK Kinase and Autophagy by GABARAP Trafficking from the Centrosome Is Regulated by WAC and GM130

Graphical Abstract

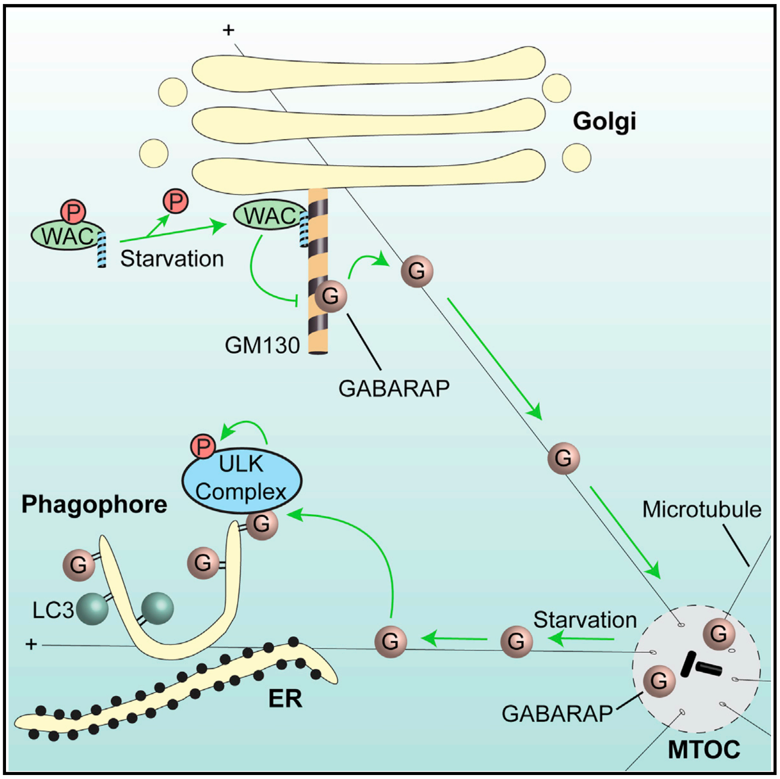

Highlights

- WAC is required for ULK activation and autophagy and interacts with GM130
- WAC suppresses GM130 binding to GABARAP and activates autophagy
- GABARAP traffics from the pericentriolar matrix to forming autophagosomes
- WAC controls the non-hierarchical activation of ULK complex by centrosomal GABARAP

Authors

Justin Joachim, Harold B.J. Jefferies, Minoo Razi, ..., Probir Chakravarty, Delphine Judith, Sharon A. Tooze

Correspondence

sharon.tooze@crick.ac.uk

In Brief

WAC and GM130 are two Golgi proteins that regulate autophagy. Joachim et al. show that WAC inhibits binding of the autophagy protein GABARAP to the vesicle tethering golgin GM130. This allows trafficking of the centrosomal pool of GABARAP to forming autophagosomes and GABARAP-mediated activation of the autophagy-initiating ULK1 kinase.

Accession Numbers

GSE66475

CrossMark

Joachim et al., 2015, Molecular Cell 60, 899–913

December 17, 2015 ©2015 The Authors
http://dx.doi.org/10.1016/j.molcel.2015.11.018

CellPress

Activation of ULK Kinase and Autophagy by GABARAP Trafficking from the Centrosome Is Regulated by WAC and GM130

Justin Joachim,[1] Harold B.J. Jefferies,[1] Minoo Razi,[1] David Frith,[2] Ambrosius P. Snijders,[2] Probir Chakravarty,[3] Delphine Judith,[1] and Sharon A. Tooze[1,*]
[1]Molecular Cell Biology of Autophagy
[2]Mass Spectrometry
[3]Bioinformatics Core
The Francis Crick Institute, Lincoln's Inn Fields Laboratories, 44 Lincoln's Inn Fields, London WC2A 3LY, UK
*Correspondence: sharon.tooze@crick.ac.uk
http://dx.doi.org/10.1016/j.molcel.2015.11.018
This is an open access article under the CC BY license (http://creativecommons.org/licenses/by/4.0/).

SUMMARY

Starvation-induced autophagy requires activation of the ULK complex at the phagophore. Two Golgi proteins, WAC and GM130, regulate autophagy, however their mechanism of regulation is unknown. In search of novel interaction partners of WAC, we found that GM130 directly interacts with WAC, and this interaction is required for autophagy. WAC is bound to the Golgi by GM130. WAC and GM130 interact with the Atg8 homolog GABARAP and regulate its subcellular localization. GABARAP is on the pericentriolar matrix, and this dynamic pool contributes to autophagosome formation. Tethering of GABARAP to the Golgi by GM130 inhibits autophagy, demonstrating an unexpected role for a golgin. WAC suppresses GM130 binding to GABARAP, regulating starvation-induced centrosomal GABARAP delivery to the phagophore. GABARAP, unlipidated and lipidated, but not LC3B, GABARAPL1, and GATE-16, specifically promotes ULK kinase activation dependent on the ULK1 LIR motif, elucidating a unique non-hierarchical role for GABARAP in starvation-induced activation of autophagy.

INTRODUCTION

Cellular homeostasis requires apposition of anabolic and catabolic pathways acting in coordination to regulate protein synthesis, trafficking, and secretion. Autophagy, a conserved catabolic pathway, delivers cytosolic cargo, sequestered by autophagosomes, to the lysosome for degradation. Degraded cargo is recycled for protein synthesis and metabolism. Autophagosomes also sequester damaged organelles, misfolded proteins, or pathogens to maintain cell health and prevent infection.

The formation of autophagosomes occurs from subdomains of the ER, mediated by ATG9-positive vesicles, followed by activation and recruitment of the ULK kinase complex and the class III PtdIns(3) (phosphatidylinositol(3)) kinase complex containing BECLIN 1 and ATG14. Association of ATG14 to the ER facilitates production of PtdIns(3)P and establishes a DFCP1-rich omegasome from which the phagophore, the nascent autophagosome, forms (Lamb et al., 2013). Further expansion of the phagophore requires recruitment of lipidated Atg8 proteins via attachment of phosphatidylethanolamine (PE) on their C-terminal glycine by a ubiquitination-like pathway. The lipidation machinery is recruited to the phagophore membrane by the PtdIns(3)P-effector WIPI2B (Dooley et al., 2014).

The mammalian Atg8 family includes LC3A, LC3B, and LC3C, GABARAP, GABARAPL1, and GABARAPL2 (or GATE-16), which perform three functions in autophagy: first, to mediate expansion and closure of the phagophore membrane; second, as cargo receptors to recruit cytoplasmic cargo through the LIR (LC3-interacting region) motif (Slobodkin and Elazar, 2013); and third, as adaptors to recruit signaling and trafficking proteins and the autophagy machinery to the autophagosome (Birgisdottir et al., 2013; Stolz et al., 2014).

To uncover novel regulators of starvation-induced autophagy, we performed a siGenome screen (McKnight et al., 2012) and identified WAC (WW domain-containing adaptor with coiled coil), which is required for starvation-induced autophagy. WAC is in the nucleus and cytoplasm and associates with the Golgi complex. Nuclear WAC binds to RNA Polymerase II and the E3 ligase complex RNF20/40 to facilitate transcription-coupled H2B monoubiquitination at lysine 120 (Zhang and Yu, 2011), which is associated with sites of active transcription (Karpiuk et al., 2012). Golgi WAC interacts with the deubiquitinase VCIP135, which binds VCP/p97 (Totsukawa et al., 2011). Here, starting from the identification of GM130 (GOLGA2) as a novel interactor of WAC, we show that WAC is tethered to the Golgi by GM130, and while WAC positively regulates autophagy, GM130 is a negative regulator. GM130 is a coiled-coil domain *cis*-Golgi matrix tethering protein required for vesicle trafficking and Golgi maintenance through mitosis (Nakamura, 2010).

Furthermore, we show that WAC and GM130 interact with GABARAP and control its subcellular localization. GABARAP is

on the Golgi and the pericentriolar matrix (PCM): tethering of GABARAP to the Golgi by GM130 inhibits autophagy, while the PCM pool of GABARAP contributes to autophagosome formation. WAC suppresses GM130 binding to GABARAP, regulating the PCM reservoir of GABARAP. GABARAP association with the PCM does not require lipidation. A photoconvertible GABARAP shows that PCM-associated GABARAP traffics to forming autophagosomes and GABARAP trafficking to the autophagosome requires WAC. Lastly, our data show that like WAC, GABARAP is required for ULK kinase activation and has a non-hierarchical function in formation of autophagosomes.

RESULTS

WAC Drives Starvation-Induced Autophagy and ULK1 Kinase Activation

In confirmation of our previous results (McKnight et al., 2012), WAC depletion using siRNA reduced LC3 lipidation during starvation and caused an increase in SQSTM1 (better known as p62) levels under basal conditions (Figures S1A–S1C). Decreased LC3-II accumulation after WAC depletion was rescued with siRNA-resistant GFP-WAC (Figure 1A).

To understand the role of WAC during autophagy, we determined the stage WAC acted to promote autophagy. In nutrient-rich conditions, mTORC1 phosphorylates and represses ULK1 kinase activity (Chan, 2009). Starvation inhibits mTORC1, allowing ULK1 activation and phosphorylation of the ULK complex member ATG13 on Ser318 (Alers et al., 2012). A time course of starvation in WAC-depleted cells revealed that mTORC1 inactivation, measured by loss of phosphorylation of ULK1 at Ser757 and S6 at Ser240/244, was unaffected (Figures 1B–1C and Figure S1D). In contrast, ATG13 Ser318 phosphorylation was reduced after WAC depletion, suggesting that ULK1 activation, not mTORC1 deactivation, is impaired by loss of WAC.

We next assessed WIPI2 puncta formation, which correlates with the amount of PtdIns(3)P produced by the BECLIN 1, ATG14-containing class III PtdIns(3) kinase complex (Dooley et al., 2014) and is a measure of the activation of the autophagy-specific PtdIns(3) kinase. WIPI2 puncta formation, but not protein levels, was reduced by WAC depletion in starved or Torin-treated cells (Figures 1D–1F, and Figures S1E–S1H). Moreover, siRNA-resistant Myc-WAC could rescue WIPI2 puncta formation, and thus PtdIns(3) kinase activation, during starvation (Figure S1I).

Finally, starvation-induced ATG9 trafficking to forming autophagosomes, which is required for autophagy (Orsi et al., 2012; Young et al., 2006), was inhibited by WAC depletion. ATG9 remained localized in the Golgi region (Figure 1G) to an extent similar to that of ULK1 depletion. In short, during amino acid starvation WAC promotes ULK1 activation and the initiation of autophagosome formation.

WAC Regulates Autophagy Independently of Its Nuclear Function

WAC localizes to the nucleus and the Golgi (Totsukawa et al., 2011; Zhang and Yu, 2011). To determine if WAC affects autophagy through its nuclear or cytoplasmic function, we performed a microarray analysis after knockdown of the nuclear complex of WAC/RNF20/RNF40. This generated an overlapping set of 301 potential gene targets that were significantly ($p < 0.05$) down- or upregulated by depletion of the nuclear complex (Figure 1H and Table S1). Analysis of the 301 hits (Table S1) revealed that p53 and p53 target genes ($p = 0.00279$) were significantly downregulated. This agrees with data that WAC regulates p53 targets and that RNF20 regulates p53 expression (Shema et al., 2008; Zhang and Yu, 2011). Autophagy, membrane trafficking, or lysosomal biogenesis pathways were not significantly up- or downregulated. Thus, unlike TFEB or ZKSCAN3 (Füllgrabe et al., 2014), the WAC/RNF20/40 complex does not regulate mRNA expression of the autophagy/lysosome pathway. Finally, knockdown of RNF40 did not affect LC3 lipidation or ULK1 signaling to ATG13 (Figures S1J and S1K).

WAC Directly Interacts with GM130 through Its Conserved Coiled-Coil Domain

WAC interacts with Flag-HA-BECLIN 1 (Behrends et al., 2010) and GFP-BECLIN 1 (McKnight et al., 2012), but not endogenous BECLIN 1 (data not shown). To identify novel binding partners of WAC that regulate autophagy, we used pull-down experiments followed by mass spectrometry. A human *WAC* gene in a bacterial artificial chromosome (BAC) was modified with a C-terminal FLAP tag (Poser et al., 2008) (see Figure S2A) and used to generate a HeLa cell line expressing WAC-FLAP; all four isoforms of WAC were tagged and expressed at levels similar to those of endogenous WAC (Figures S2B and S2D). WAC-FLAP localizes to the nucleus and Golgi and binds RNF40 (Figures S2C and S2D). WAC immunoprecipitations from HEK293 cells, GFP immunoprecipitations from WAC-FLAP HeLa cells and mass spectrometry identified the *cis*-Golgi coiled-coil tether GM130 as a WAC interactor (Figures S2E–S2G), as well as the WAC interactors RNF20, RNF40, and UBE2A.

We confirmed binding of endogenous WAC to GM130 by co-immunoprecipitation (Figure 2A). Upon starvation of HEK293 or HeLa WAC-FLAP cells, the amount of GM130, but not RNF40, interacting with WAC or WAC-FLAP increased (Figures 2B–2E). As WAC is phosphorylated (Xu and Arnaout, 2002) we asked if phosphorylation regulates the WAC-GM130 interaction. Using Phos-tag SDS-PAGE gels after 30 min starvation, WAC migrated faster, suggesting that WAC is dephosphorylated upon starvation (Figures 2F and 2G). To test if dephosphorylation enhanced the WAC-GM130 interaction, we salt washed GFP-WAC to remove bound proteins, including GM130, and treated with lambda phosphatase (Figure 2H). Dephosphorylated salt washed GFP-WAC had enhanced binding to endogenous GM130 compared to untreated salt washed GFP-WAC or N-terminal WAC (see Figure 2J). These results suggest that WAC is dephosphorylated during starvation and this enhances its ability to bind GM130.

The highly conserved WAC C-terminal coiled-coil (CC) domain was required for interaction with GM130 (Figures 2I–2J and Figure S2H). Truncation analyses of WAC revealed that a 10 amino acid sequence in the WAC CC domain (aa610–620) containing one heptad (aa612–618) was required for GM130 binding (Figure 2K and Figure S2I). RNF20/40 bind the C terminus of WAC including the CC domain (Zhang and Yu, 2011). Isoleucine 626

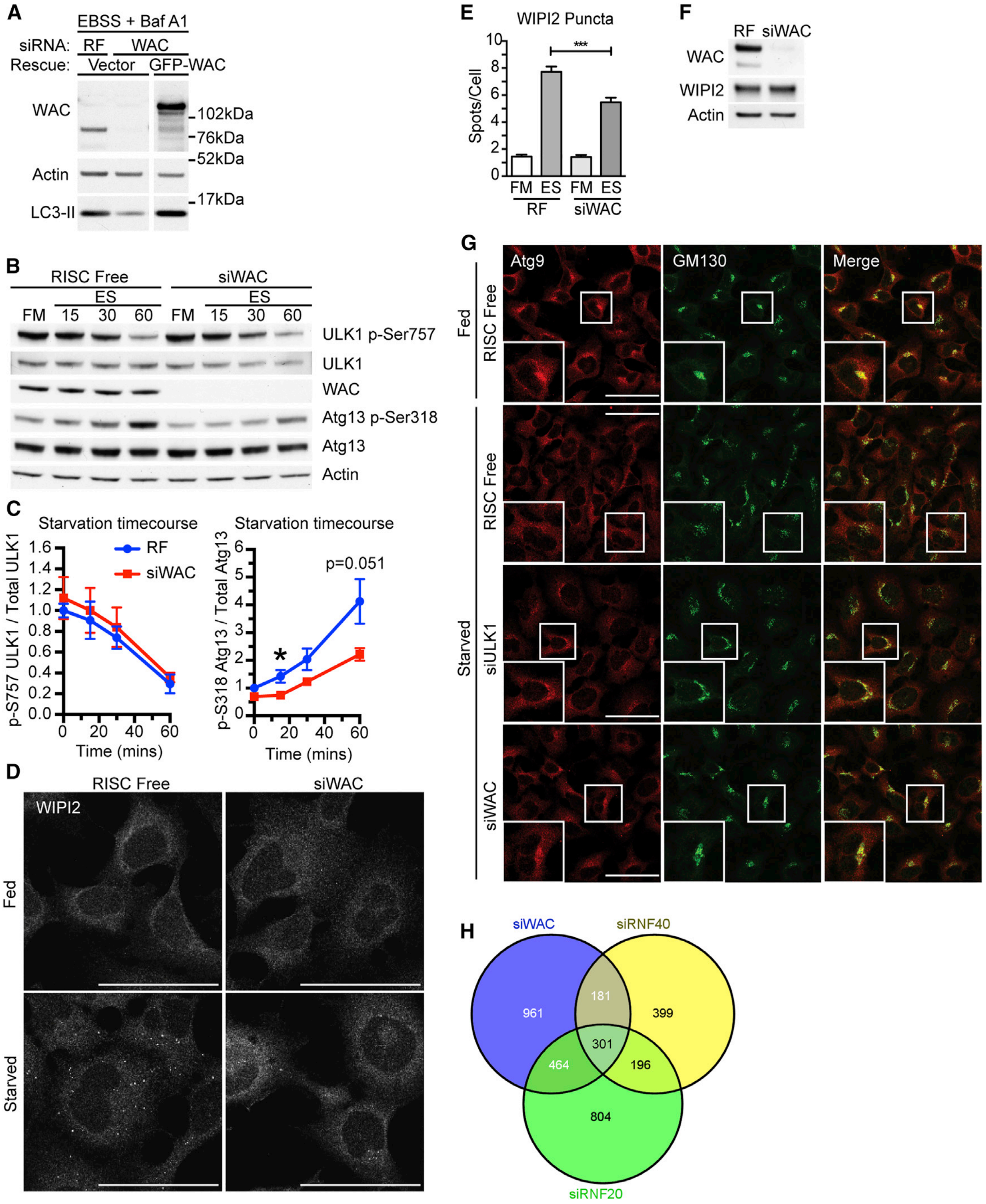

Figure 1. WAC Promotes Starvation-Induced Autophagy and ULK1 Kinase Activation

(A) siRNA-resistant EGFP-WAC or vector was expressed in HEK293A cells treated for 72 hr with either RISC-free (RF) or WAC siRNA. After 2 hr starvation with EBSS and BafA1, cells were analyzed by immunoblot.

(B) HEK293A cells treated with RF or WAC siRNA for 72 hr were incubated in full medium (FM) or EBSS (ES) for 15, 30, and 60 min.

(C) Quantification of (B); statistical analysis using unpaired Student's t test, mean ± SEM, n = 3 experiments, *p ≤ 0.05.

(D) HEK293A cells were treated with RF or WAC siRNA for 72 hr then starved 2 hr, fixed, and labeled with WIPI2. Scale bars, 50 μm.

(E) WIPI2 puncta in (D) were counted. Mean ± SEM from n = 2 experiments, >150 cells counted per condition, unpaired Student's t test, ***p ≤ 0.001.

(F) HEK293A cells treated with RF or WAC siRNA for 72 hr before immunoblotting.

(G) HEK293A cells were treated with RF, ULK1, or WAC siRNA for 72 hr, incubated in fed or 2 hr EBSS (Starved), and labeled for Atg9 and GM130. Scale bars, 50 μm.

(H) Venn diagram of number of genes significantly (p < 0.05) up- or downregulated in HEK293A cells treated with WAC, RNF20, or RNF40 siRNA for 72 hr versus RF control.

See also Figure S1 and Table S1. Excised lanes are indicated by a gap, and remaining lanes are from the same gel.

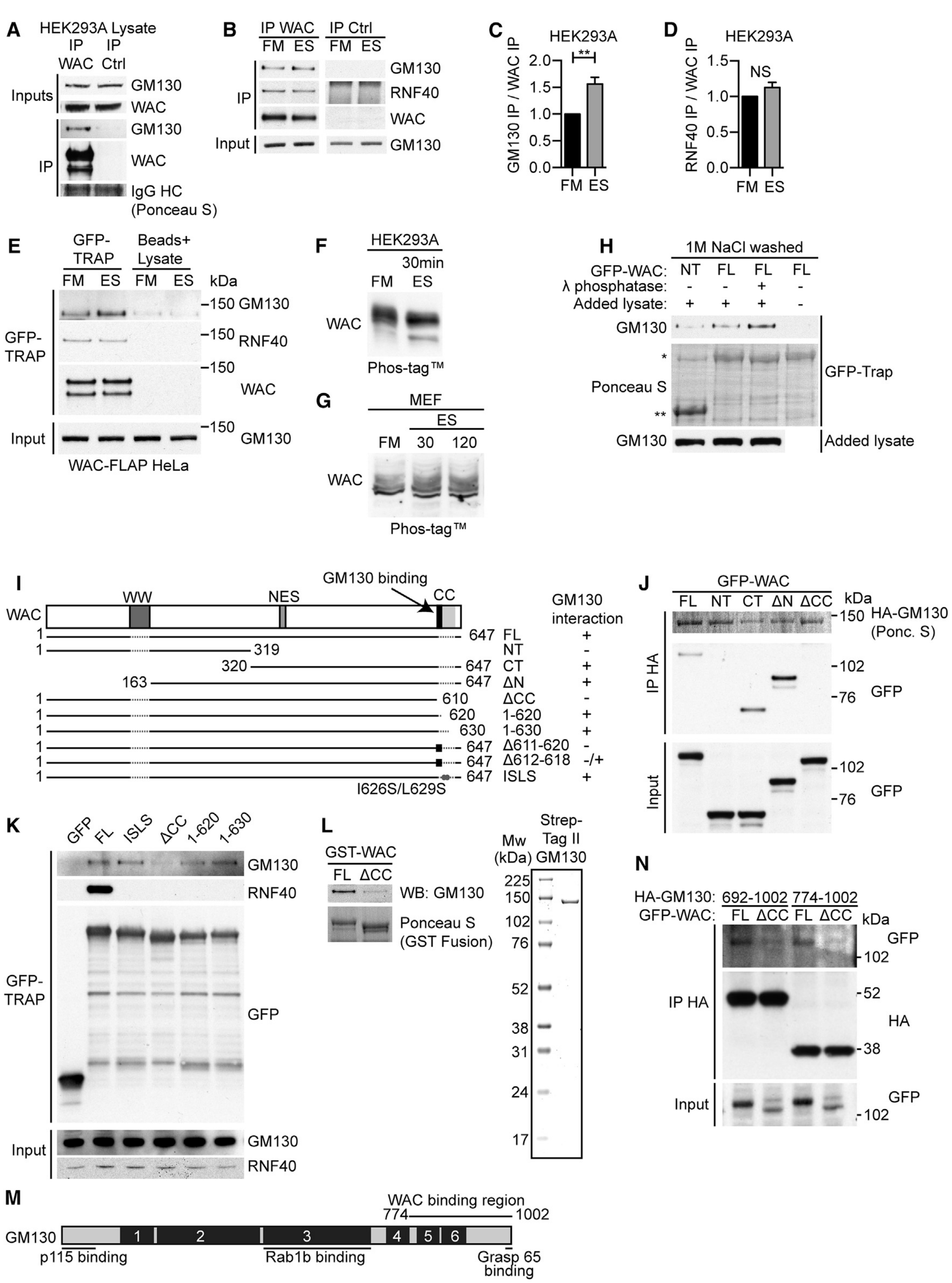

Figure 2. The WAC-GM130 Interaction Is Direct and Independent of RNF40

(A) Anti-WAC and anti-GFP immunoprecipitates analyzed by immunoblotting. HC, IgG heavy chain.

(B) HEK293A cells in full medium (FM) or EBSS (ES) for 2 hr prior to lysis, followed by treatment as in (A).

(C) Quantification of GM130 as in (B) after normalization to immunoprecipitated WAC. Statistical analysis using unpaired Student's t test, mean ± SEM, n = 4, $^{**}p \leq 0.01$.

(D) Quantification of RNF40 as in (B) after normalization to immunoprecipitated WAC. Mean ± SEM, n = 2.

(legend continued on next page)

and leucine 629, at the "a" and "d" positions of a CC heptad respectively (I626S/L629S mutant), are essential for binding to RNF40 but not GM130 (Figure 2K). Finally, recombinant GST-WAC bound directly to purified GM130 and required the WAC CC domain (Figure 2L).

GM130 interacts with proteins involved in Golgi trafficking including USO1 (p115), RAB1B, RAB33B, and GRASP65 (Nakamura, 2010). Truncations of GM130 showed that full-length WAC, but not WAC ΔCC, binds to the C-terminal of GM130 at aa774–1002. This includes the fifth and sixth CC domains of GM130 as well as the GRASP65 binding site (Figures 2M and 2N).

GM130 Tethers WAC to the Golgi, and the Interaction Promotes Autophagosome Formation

WAC colocalizes with GM130 (Totsukawa et al., 2011), and in addition with the COPI coat protein βCOP and the ERGIC marker ERGIC-53 (Figure 3A), but less with the TGN markers p230 (GOLGA4) and TGN46 (Figure 3B). To test if GM130 is required for Golgi localization of WAC, we knocked down GM130. In cells with no detectable GM130, we never saw juxtanuclear WAC localization, even though the TGN remained intact (Figure 3C). GFP-WAC but not ΔCC WAC, localized to the Golgi region (Figure 3D). In addition, we performed a knockdown rescue experiment. In WAC knockdown cells, siRNA-resistant GFP-tagged full-length WAC but not ΔCC WAC, was detected on the Golgi (Figure S3A). We conclude that GM130 tethers WAC to the Golgi.

Using the recently developed mitochondrially targeted golgins that can tether vesicles to mitochondria (Wong and Munro, 2014) (see Figure S3B), we show that GM130-MAO can target GFP-WAC to mitochondria but GFP-WAC NT remained exclusively nuclear (Figure 3E). We next asked if the Golgi and nuclear pools of WAC are exchangeable. WAC has a putative nuclear export signal (NES) (Figure 2I), so we inhibited CRM-1-dependent nuclear export using leptomycin B (LMB) (Figure S3C). In contrast to control and 2 hr LMB, 24 hr LMB inhibited GFP-WAC targeting to the mitochondria. p62 was retained in the nucleus after LMB treatment, as expected (Pankiv et al., 2010). This suggests that in contrast to the rapid nuclear shuttling of p62, WAC is retained in the cytoplasm by GM130 and export of nuclear WAC is required to maintain the cytoplasmic WAC population.

Finally, knockdown and rescue experiments showed that the GM130-interacting CC domain of WAC was required to rescue LC3B spot formation under starvation conditions (Figures 3F and 3G), implying that the WAC-GM130 interaction promotes autophagosome formation.

GM130 Is a Negative Regulator of Autophagy and Interacts with GABARAP

Loss of GM130 affects cell growth and increases autophagy in tumor cells and in a lung cancer mouse model (Chang et al., 2012). Similarly, in HEK293 cells depletion of GM130 increased LC3-II lipidation in both fed and starved cells, increased WIPI2 spots and basal ULK1 activation, and caused a significant decrease of p62 levels (Figures 4A–4F and Figure S4A). Overexpression of HA-GM130 reduced LC3-II levels but had no effect on p62 levels or WIPI2 spots (Figures S4B–S4E). These data imply that GM130 functions as a negative regulator of autophagy.

To probe the link between GM130 and autophagy, we investigated the localization of GM130 relative to autophagosome markers. We examined GABARAP, which localizes to the Golgi complex (Shpilka et al., 2011), and saw that GM130 in fed cells was distributed around an enlarged GABARAP structure, as well as occasionally colocalized with starvation-induced GABARAP puncta (Figure 4G).

Do GM130 and GABARAP, or other Atg8 homologs interact? Immunoprecipitation of a panel of mammalian Atg8 homologs revealed that GM130 preferentially bound GFP-GABARAP, whereas less binding was seen with GFP-LC3 family members (Figure 4H). WAC also interacted with the Atg8s including GFP-GABARAP. Importantly, WAC and GM130 interact with endogenous GABARAP (Figure 4I). No direct GABARAP-WAC interaction could be detected (Figures S4F–S4H), however, GM130 and GABARAP interact directly, and increasing amounts of WAC disrupt this interaction (Figure 4J). Additionally, mitochondrially targeted GM130 recruits GFP-GABARAP (Figure 4K) and endogenous GABARAP to the mitochondria (Figure S4I).

GABARAP Localizes to the Centrosome and This Is Controlled by Nutrient Status and Microtubules

Based on the localization and shape of the enlarged GABARAP-positive structure, we asked if it was positive for γ-tubulin, a marker for the PCM of the centrosome (Oakley, 2000). Using different GABARAP antibodies, we saw colocalization of GABARAP with γ-tubulin in HEK293A cells; we refer to this

(E) GFP-TRAP pull down of HeLa WAC-FLAP cell followed by immunoblot. Cells in FM or EBSS (ES) for 2 hr prior to lysis.

(F and G) WAC in FM or EBSS for 30 min in HEK293 (F) or 30 and 120 min in MEFS (G) were analyzed by Phos-tag SDS-PAGE.

(H) GFP-TRAPs of GFP-WAC full length (FL) or aa1–319 (NT), salt washed, treated or not with lambda phosphatase, incubated with HEK293 lysate, and immunoblotted with anti-GM130.

(I) Human WAC isoform 1 and deletion mutants used here: WW domain, putative nuclear export signal (NES), and C-terminal coiled-coil (CC) domain, including the GM130 binding region. GM130 binding ability is indicated. Black square, 10aa or 7aa heptad deletions. Grey circles, ISLS mutations.

(J) HEK293A cells co-transfected with 3xHA-GM130 and EGFP-WAC FL, NT, aa320–647 (CT), aa163–647 (ΔN), or aa1–610 (ΔCC) immunoprecipitated with anti-HA and immunoblotted.

(K) GFP-TRAP of cells expressing EGFP or EGFP-WAC FL, ISLS, ΔCC, 1–620, or 1–630 and immunoblot of GM130 and RNF40.

(L) GST-WAC FL or GST-WAC ΔCC incubated with purified Strep-Tag II-GM130. GST-WAC monitored by Ponceau S staining. Right, colloidal Coomassie staining of Strep-Tag II-GM130.

(M) Human GM130 isoform 1, boxes 1–6, coiled-coil domains. USO1 (p115), Rab1b, Grasp65, and WAC binding regions are indicated.

(N) HEK293A cell lysates containing EGFP-WAC FL or ΔCC were mixed with HA-GM130 aa692–1,002 or HA-GM130 aa774–1,002 lysates, immunoprecipitated with anti-HA, and analyzed by immunoblotting.

See also Figure S2. Excised lanes are indicated by a gap, and remaining lanes are from the same gel.

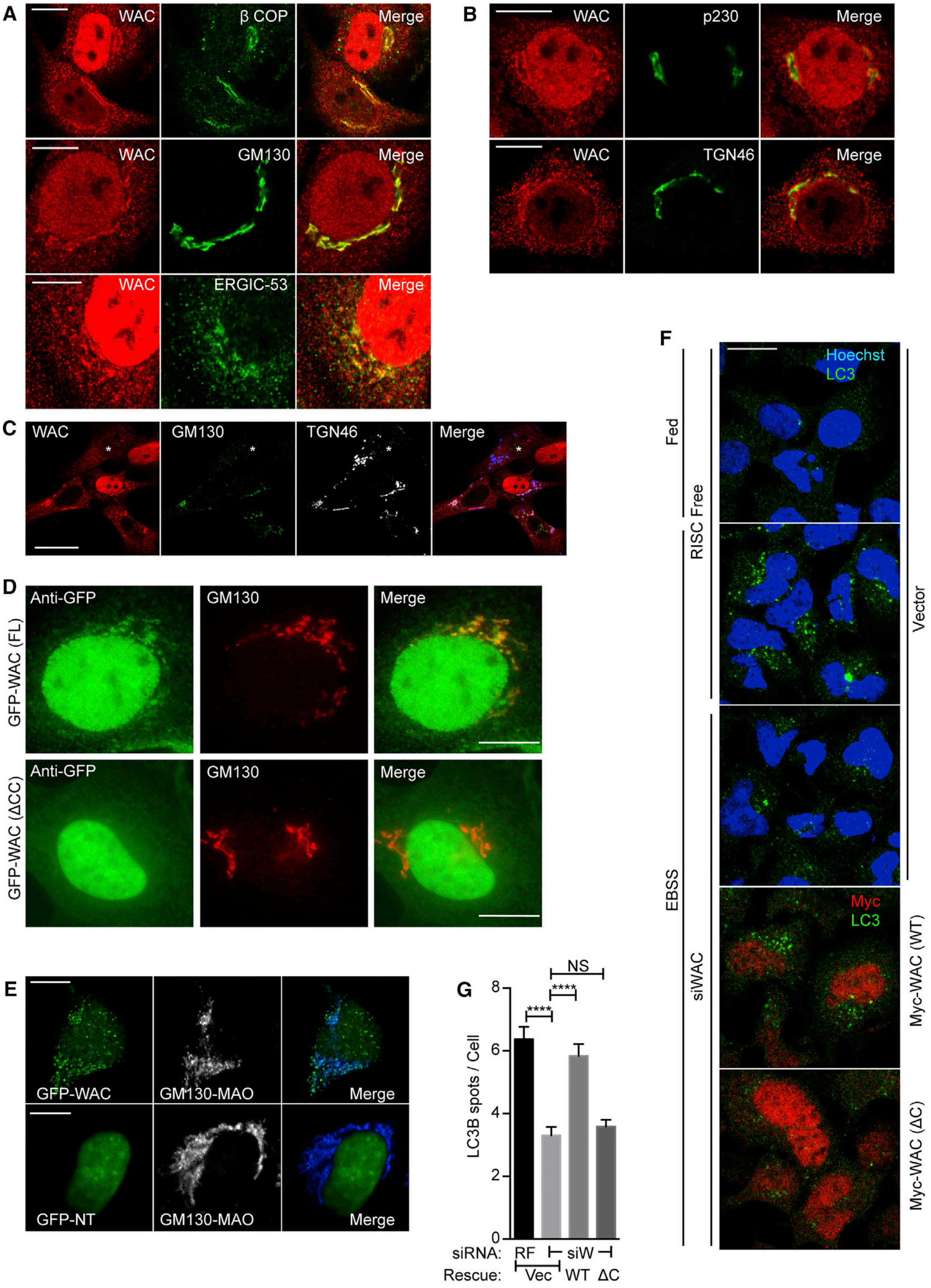

Figure 3. GM130 Tethers WAC to the Golgi, and the Interaction Is Required for Autophagosome Formation

(A and B) WAC localized with βCOP, GM130, or ERGIC-53 (A) or p230 and TGN46 (B). Scale bars, 10 μm.

(C) WAC, TGN46, and GM130 in GM130-depleted cells. (*) cell depleted of GM130. Scale bars, 25 μm.

(D) EGFP-WAC FL or ΔCC expressed after WAC depletion, labeled with indicated antibodies for epifluorescence microscopy.

(E) EGFP-WAC (FL) or aa1–319 (NT) co-expressed with GM130-ΔCterm-HA-MAO labeled with anti-HA. Scale bars, 10 μm.

(legend continued on next page)

pool as centrosomal GABARAP (Figure 5A and Figure S5A). In five other cell lines, GABARAP and γ-tubulin also colocalized (Figure 5A). These GABARAP structures were not LC3B positive (see Figure 6C). Centrosomal GABARAP also localized around Centrin-3, a centriolar marker (Middendorp et al., 2000) (Figure 5B). Furthermore, in HEK293 cells with an enlarged PCM, knockdown of GABARAP reduced centrosomal GABARAP (Figures 5C and 5D). Using a tetracycline-inducible cell line, we confirmed that GFP-GABARAP colocalized with γ-tubulin (Figure 5E). Centrosomal GABARAP is not an aggresome, as it is negative for ubiquitin and p62, even upon proteasome inhibition with MG132 (Figures S5B and S5C). Wortmannin, an inhibitor of starvation-induced GABARAP-positive autophagosomes, did not affect GABARAP localization at the centrosome (Figure 5F). Moreover, the GABARAP G116A mutant, which is not lipidated (Figure S5D), also localized to the centrosome (Figure 5G).

We asked if centrosomal GABARAP was stably associated with the PCM. GABARAP associates with microtubules (Wang and Olsen, 2000), so we disassembled microtubules with nocodazole (Figure 5H and Figures S5E and S5F) and found that centrosomal GABARAP was significantly ($p < 0.0001$) reduced (Figure 5I) and there was a concomitant increased colocalization of GABARAP with Golgi mini-stacks (Figure 5H and Figure S5F). As expected, GABARAP relocalized to nocodazole-induced Golgi mini-stacks during wortmannin treatment (Figure S5F). In addition, centrosomal GABARAP dissociates during metaphase (Figure S5G).

After starvation, we saw a highly significant ($p < 0.0001$) decrease in centrosomal GABARAP signal and concurrent formation of GABARAP puncta (Figures 5F and 5J), suggesting GABARAP relocalizes from the centrosome to autophagosomes during starvation. In contrast to nocodazole treatment, dissembling the Golgi with Brefeldin A (BFA) (Figure S5H) did not diminish centrosomal GABARAP. Importantly, as BFA does not affect autophagic flux (Weidberg et al., 2010), GABARAP formed starvation-induced puncta even in the presence of BFA.

We conclude that centrosomal GABARAP is dynamic and is affected by disruption of microtubules or starvation, and that its association with the PCM and the Golgi is not dependent on lipidation but mediated by protein interactions.

Centrosomal GABARAP Contributes to Autophagosome Formation

GABARAPs are essential for the final expansion or closure of the autophagosome (Weidberg et al., 2010). Given this, we suggest that centrosomal GABARAP acts as a reservoir for expansion of forming autophagosomes. To probe the dynamics of centrosomal GABARAP, we attached a photoconvertible fusion protein, EosFP, to the N terminus of GABARAP. EosFP is converted from green to red fluorescence upon excitation with UV light (Wiedenmann et al., 2004). EosFP-GABARAP colocalized with γ-tubulin and WIPI2 in starved HEK293 cells (Figure S6A), and EosFP-GABARAP formed rings around γ-tubulin, which are visible in live cells (Figure 6A, C and Figures S6B and S6C).

In starved live HEK293 cells, centrosomal green EosFP-GABARAP was photoconverted to red, and GABARAP-positive red spots formed while the intensity of red centrosomal EosFP-GABARAP decreased (Figure 6A, Figure S6B and Movie S1). Translocation of the photoconverted centrosomal EosFP-GABARAP to distal regions was monitored by quantifying fluorescence intensities (Figure 6B). Like autophagosomes, EosFP-GABARAP puncta were highly mobile (Kimura et al., 2008) and additionally made transient interactions with centrosomal EosFP-GABARAP (Movie S2). Strikingly, peripheral green EosFP-GABARAP spots (that existed prior to photoconversion) acquired centrosomally derived red photoconverted EosFP-GABARAP (Figure 6A and Figure S6B), implying that forming and expanding autophagosomes acquire GABARAP derived from the PCM. After time-lapse microscopy, the cells were fixed and stained with LC3 and γ-tubulin (Figure 6C and Figure S6C). Correlative confocal microscopy confirmed that the photoconverted pool of GABARAP was at the centrosome and that photoconverted centrosomal GABARAP had moved to peripheral puncta, many of which were LC3 positive (Figure 6C).

In contrast to the centrosomal pool, cytosolic EosFP-GABARAP diffused within seconds across the cell (Figures 6D–6E and Figures S6D and S6E), indicating that the red GABARAP-puncta in Figures 6A and S6B are derived from a centrosomal pool and not from unintentional photoconversion of the cytosolic pool.

Next, we treated live HEK293 cells expressing EosFP-GABARAP with nocadozole; EosFP-GABARAP was lost from the centrosome and dispersed into puncta (Figure 6F), which were not dynamic over a 20 min period after photoconversion. Correlative confocal microscopy revealed that similar to endogenous GABARAP (Figure 5H), EosFP-GABARAP was retained on Golgi mini-stacks (Figure 6G). Thus, without microtubules, centrosomal GABARAP relocalizes to the Golgi, becomes immobile and does not contribute to autophagosome formation.

WAC Inhibits GM130 Tethering of GABARAP to Maintain the Centrosomal GABARAP Reservoir and GABARAP-Mediated ULK1 Activation

Since WAC and GM130 form a complex with GABARAP (Figures 4H–4J), we asked if WAC and GM130 controlled the localization of GABARAP to the PCM or forming autophagosomes. Centrosomal GABARAP co-localized with γ-tubulin in both fed and starved cells, and in starved cells GABARAP-positive autophagosomes appeared (Figures 6H and S6F). In starvation, WAC knockdown caused a striking accumulation of GABARAP on the Golgi and ERGIC and loss of centrosomal GABARAP (Figure 6H and Figures S6F and S6G). To investigate GABARAP dynamics in WAC-depleted cells, we used EosFP-GABARAP. As expected, EosFP-GABARAP accumulated on and around the Golgi (Figures 7A and 7B). Photoconversion and time-lapse

(F) RF or WAC (siW) siRNA-treated cells for 72 hr transfected with vector (Vec), Myc-WAC (WT), or Myc-WAC aa1–610 (ΔC), starved for 2 hr with EBSS. LC3B puncta were analyzed by confocal microscopy.
(G) LC3B puncta from (F), Mean ± SEM of n = 3, >400 cells counted per condition, unpaired Student's t test, ****$p \leq 0.0001$.
See also Figure S3.

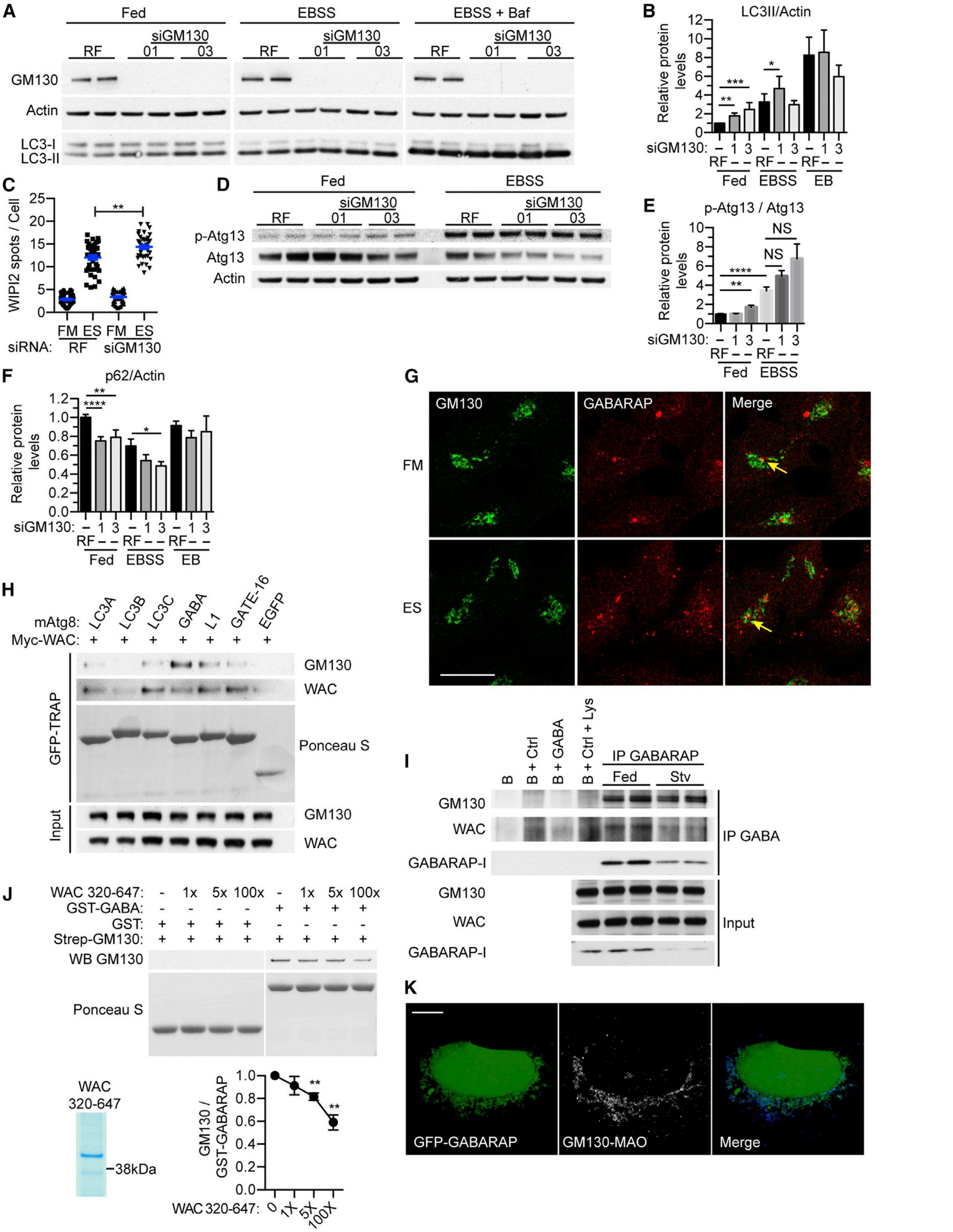

Figure 4. GM130 Is a Negative Regulator of the Early Stages of Autophagy and Interacts with GABARAP

(A) HEK293A cells treated with RF or GM130 siRNA-01 or -03 incubated in full medium (FM), or EBSS (ES) with or without BafA for 2 hr.

(B) LC3-II levels from (A). Mean ± SEM of n = 4, Mann-Whitney test, *p ≤ 0.05.

(legend continued on next page)

microscopy showed that the pool of Golgi-accumulated EosFP-GABARAP is relatively immobile and does not form cytoplasmic GABARAP spots (Figures 7A and 7B, Movie S3). Importantly, in WAC knockdown cells there was an increase in the GM130-GABARAP interaction (Figures 7C and 7D). Thus, in the absence of WAC, GABARAP, likely non-lipidated, relocalizes from the centrosomal pool to the Golgi and ERGIC, is tethered by GM130, and does not contribute to autophagosome formation.

GM130 regulates centrosome morphology and function (Kodani and Sütterlin, 2008), and after GM130 knockdown, we saw an increase in the size and intensity of the centrosomal GABARAP pool (Figure 6H and Figure S6F). These data suggest that WAC and GM130 control the localization of centrosomal GABARAP in a reciprocal manner and that in cells WAC negatively regulates GABARAP-GM130 binding.

As WAC depletion reduced ATG13 phosphorylation (Figures 1B–1C and Figure S1D), we asked if GABARAP knockdown also attenuates ULK1 activation. ATG13 p-Ser318 levels were significantly decreased after depletion of GABARAP but not LC3B, GABARAPL1, or GATE-16 (Figures 7E–7H and Figure S7A). Centrosomal GABARAP is not lipidated, and non-lipidated GABARAP interacts constitutively with ULK1 (Figure 7I) in the cytosol and on membranes in a LIR-dependent manner (Figure 7J and Figures S7B and S7C). To define the membrane compartment harboring the ULK1-GABARAP complex, we immunoisolated DFCP-1-positive omegasomes and phagophores from starved cells. These DFCP-1 structures were positive for endogenous ULK1, WIPI2, GABARAP, and overexpressed wild-type ULK1, but not ΔLIR ULK1 (Figures S7D and S7E). GABARAP G116A was also found in similar amounts to GABARAP (Figure 7K), and immunoprecipitation of G116A revealed that it interacts with ULK1 on GFP-DFCP1 membranes (Figure 7L).

Out of the Atg8 family, ULK complex preferentially binds GABARAP through a LIR motif (Alemu et al., 2012; Kraft et al., 2012). Overexpression of HA-ULK1 in fed HEK293 cells was sufficient to drive ATG13-FLAG phosphorylation even under basal conditions and when the ULK1 LIR was mutated (Figure 7M). However, in the presence of GFP-GABARAP and the unlipidated G116A mutant, but not GFP-LC3B, the ULK1 LIR was required for maximal phosphorylation of ATG13-FLAG (Figure 7M and Figure S7F). Thus, GABARAP specifically promotes ULK1 activity through the ULK1 LIR motif and independently of lipidation.

In conclusion, we have shown a specific regulation of the centrosomal GABARAP pool by WAC and GM130 that correlates with initiation of autophagy, suggesting that centrosomally derived GABARAP signals back to autophagy initiation by sustaining ULK1 activity and explains why WAC, which inhibits GABARAP-GM130 interaction, also promotes ULK1 activation.

DISCUSSION

WAC and GM130 are two regulators of autophagy whose mechanism of action is unresolved and previously unconnected (McKnight et al., 2012; Totsukawa et al., 2011). Here we show that WAC directly binds to GM130 and that WAC-GM130 binding is required for autophagosome formation. WAC functions by suppressing GM130 binding to GABARAP, allowing the maintenance of a centrosomal GABARAP reservoir. Centrosomal GABARAP and Golgi WAC coordinate and regulate autophagosome formation through ULK1 activation.

Like GM130, WAC localizes to and co-purifies with the Golgi complex (Totsukawa et al., 2011), remaining on the Golgi after 1 M KCl wash (Nakamura et al., 1995; Totsukawa et al., 2011). We show that direct binding to GM130 tethers WAC to the Golgi complex and this is likely regulated by dephosphorylation of WAC during starvation. WAC-GM130 binding is independent of the RNF20/40 complex. The 10aa (aa611–620) of WAC are essential for GM130 binding and these 10aa are highly conserved back to *D. melanogaster* (Xu and Arnaout, 2002). WAC and GM130 form a stable complex: WAC remains bound to mitochondrially targeted GM130, even after 2 hr with LMB. GM130 has numerous functions, including vesicle tethering, signaling, and controlling mitosis (Baschieri et al., 2014; Nakamura, 2010; Wei et al., 2015).

GM130 can cycle between the ERGIC and the *cis*-Golgi (Marra et al., 2001), which explains the localization of GABARAP and WAC to these compartments. The binding of GM130 to GABARAP is direct and not regulated by starvation, and our live-cell imaging data suggest that the Golgi pool of GABARAP does not participate in autophagy. The strong preference of GM130 for GABARAP, and not LC3B for example, indicates a level of specificity that remains to be investigated.

In basal conditions, the Golgi acts as a hub for autophagy proteins such as BECLIN 1, GATE-16, ATG9, ATG16, GABARAP, and LC3 (Guo et al., 2012; Itoh et al., 2008; Kittler et al., 2001; Sagiv et al., 2000; Shoji-Kawata et al., 2013; Young et al., 2006), and Golgi-derived membranes are proposed to contribute to autophagosome biogenesis (Abada and Elazar, 2014). Recently, contact sites between the Golgi and the phagophore were identified that could be sites of lipid transfer (Biazik et al., 2015). Moreover, the Golgi complex is intimately linked with the centrosome, and GM130 controls centrosome morphology

(C) HEK293A cells treated with RF or GM130 siRNA incubated in full medium (FM) or EBSS (ES) for 2 hr. Statistical analysis using unpaired Student's t test, mean ± SEM, n = 3, **p ≤ 0.01. 30 fields of cells were analyzed per condition.

(D) HEK293A cells treated with RF or GM130 siRNA-01 or -03 incubated in full medium (FM) or EBSS (ES) for 2 hr and immunoblot.

(E) Quantification of (D). Statistical analysis using unpaired Student's t test, mean ± SEM, n = 5, **p ≤ 0.01.

(F) p62 degradation from Figure S4A, mean ± SEM of n = 5, Mann-Whitney test, *p ≤ 0.05.

(G) HEK293A cells in FM or EBSS for 2 hr. Arrows indicate colocalization. Scale bars, 20 μm.

(H) Myc-WAC and EGFP, EGFP-LC3A, LC3B, LC3C, GABARAP, GABARAPL1, or GATE-16 co-expressed, followed by GFP-TRAP and immunoblot.

(I) Immunoprecipitation of GABARAP from fed or starved cells and immunoblot analysis. B, beads; Ctrl, Anti-GFP; GABA, Anti-GABARAP.

(J) Purified Strep II-GM130 was incubated with recombinant WAC 320–647 before binding to recombinant GST or GST-GABARAP beads and immunoblot. Statistical analysis using unpaired Student's t test, mean ± SEM, n = 3. **p ≤ 0.01.

(K) Co-expressed EGFP-GABARAP and GM130-ΔCterm-HA-MAO labeled with anti-HA. Scale bars, 10 μm.

See also Figure S4. Excised lanes are indicated by a gap, and remaining lanes are from the same gel.

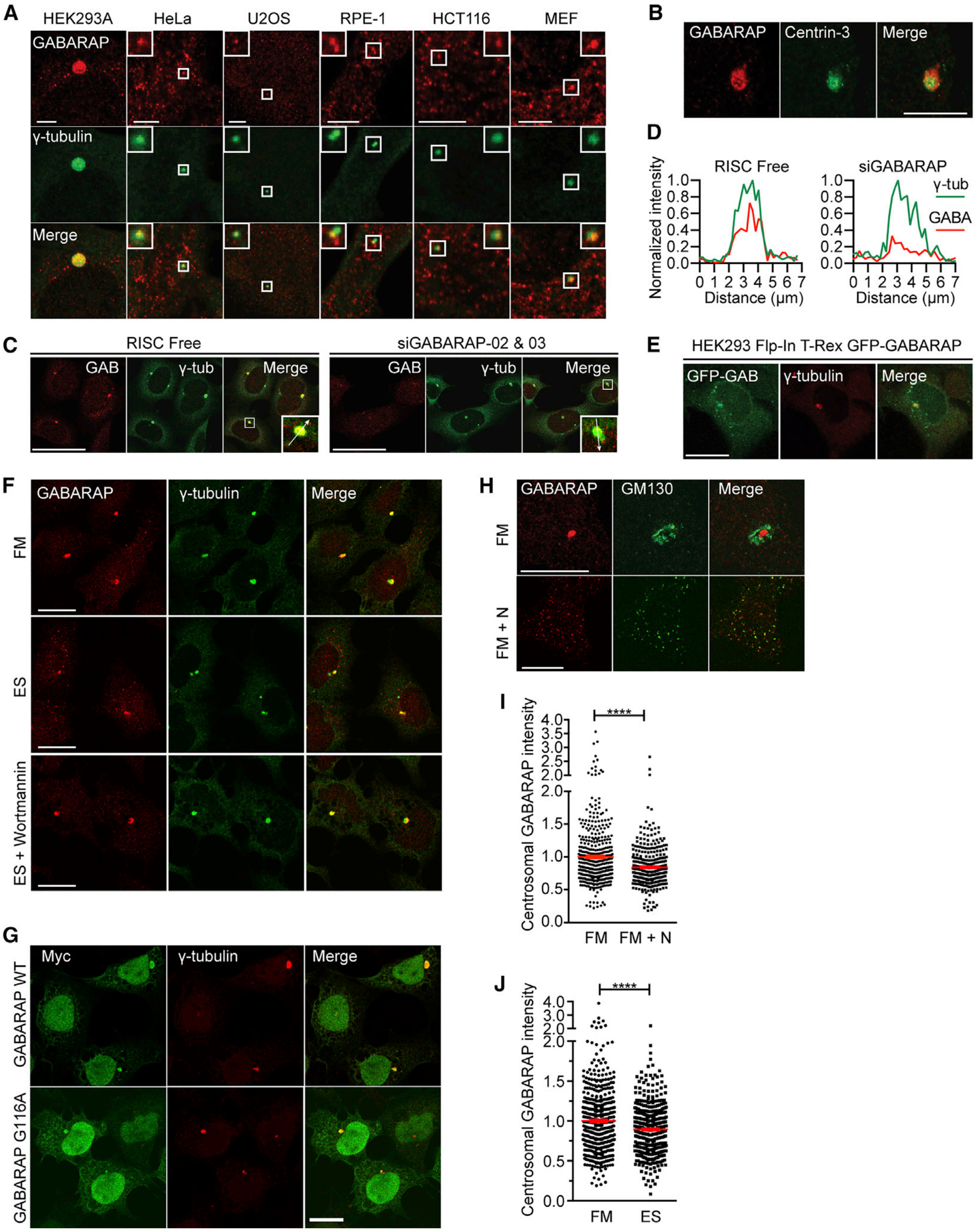

Figure 5. Non-lipidated GABARAP Localization at the Centrosome Is Regulated by Starvation and Microtubules

(A) HEK293A, HeLa, U2OS, RPE-1, HCT116, and MEF cells were labeled as indicated. Scale bars, 5 µm.

(B) HEK293A cells labeled as indicated. Scale bars, 10 µm.

(C) HEK293A cells were treated for 72 hr with RF or GABARAP siRNAs and labeled as in (A). Scale bars, 50 µm. Inset, line scan.

(D) Line scans of (C).

(E) GFP-GABARAP expression in HEK293 Flp-In T-Rex cells after tetracycline for 24 hr. Scale bars, 20 µm.

(F) HEK293A cells incubated in FM, EBSS (ES), or EBSS with wortmannin for 2 hr. Scale bars, 20 µm.

(G) HEK293A cells expressing Myc-GABARAP or G116A mutant labeled as indicated. Scale bars, 20 µm.

(H) HEK293A cells in FM or FM plus nocodazole (FM + N) for 5 hr. Scale bars, 20 µm.

(I) Quantification of centrosomal GABARAP as in (H). Mean ± SEM of n = 3, >300 cells counted per condition, unpaired Student's t test, ****p $\leq$ 0.0001.

(J) Quantification of centrosomal GABARAP after 2 hr with FM or EBSS. Mean ± SEM of n = 3, >420 cells counted per condition, unpaired Student's t test, ****p $\leq$ 0.0001.

See also Figure S5.

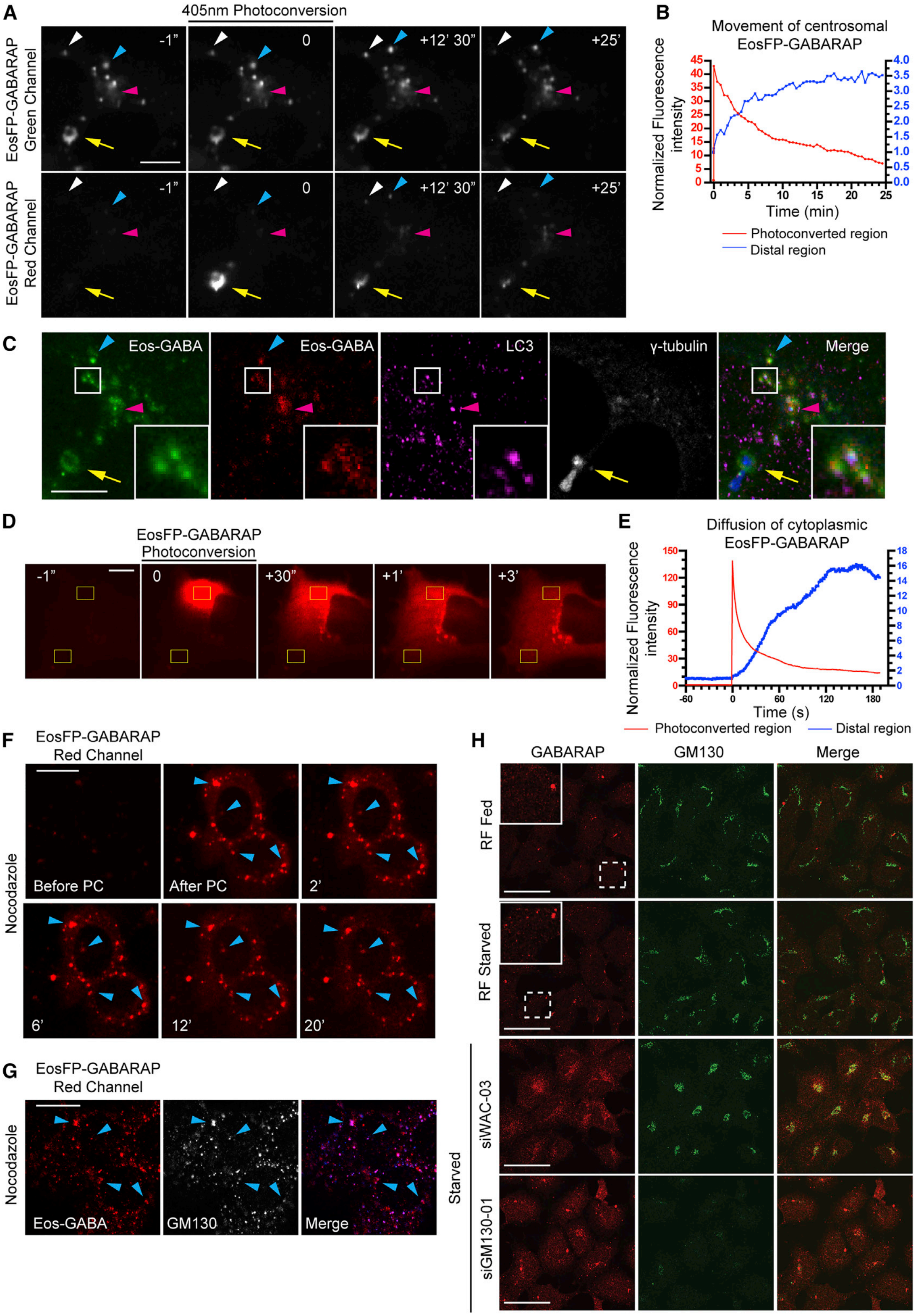

Figure 6. Centrosomal GABARAP Contributes to Autophagosome Formation

(A) EosFP-GABARAP in starved cells was imaged every 30 s using a swept field confocal microscope. 5 × 1 s images were captured prior to photoconversion (PC). PC moment is set to 0 s. Yellow arrow, photoconverted region. Magenta arrowhead, distal region. White and blue arrows track defined puncta. Scale bars, 10 μm.

(legend continued on next page)

and function (Kodani et al., 2009; Kodani and Sütterlin, 2008). Golgi proteins such as AKAP450 (which binds GM130), GMAP-210, and HOOK3 connect the Golgi ribbon to the microtubule network and centrosome (Rios, 2014). Interestingly, ATG4D, which processes GABARAPL1, is localized to the centrosome (Betin and Lane, 2009). Here, we show an unexpected localization of non-lipidated GABARAP at the PCM.

We show that GM130 and WAC allow specific control of the centrosomal GABARAP pool. This pool of GABARAP is non-lipidated and how it associates with the PCM remains to be determined. Depletion of GM130 increases GABARAP residency on the centrosome and drives autophagy. In contrast, WAC knockdown results in loss of centrosomal GABARAP and relocalization to the ERGIC and Golgi due to increased GM130 binding. GABARAP directly binds microtubules (Wang and Olsen, 2000) and we speculate that microtubules may allow GABARAP transfer from the Golgi to the centrosome and from the centrosome to the autophagosome formation site. In the absence of microtubules, GABARAP relocalizes from the centrosome to Golgi ministacks, which are located at ER exit sites (Cole et al., 1996). However, the role of microtubules in these events remains to be clarified.

The Atg8s are critical autophagy proteins. In yeast there is one Atg8 and no GM130 or WAC homologs (Nakamura et al., 1997; Xu and Arnaout, 2002). In mammals there are 7 Atg8 homologs, the LC3s, and GABARAPs (Slobodkin and Elazar, 2013). Although there is evidence of both redundancy and specialization of the LC3 and GABARAP family members, a comprehensive understanding of regulation and function of these proteins is lacking. In addition, many LC3- and GABARAP-interacting proteins have been identified, raising questions on how these interactions are controlled (Birgisdottir et al., 2013).

Our work suggests that non-lipidated centrosomal GABARAP is transported to autophagosome formation sites where it localizes to autophagosomal membranes. Although the GABARAP subfamily promotes phagophore closure (Weidberg et al., 2010), it also binds the ULK complex. The ULK complex members ULK1/2, FIP200, and Atg13 bind GABARAP preferentially via LC3-interacting regions (LIRs) (Alemu et al., 2012; Kraft et al., 2012; Okazaki et al., 2000). The identification of LIRs in the ULK complex suggests that the LC3 and GABARAP subfamilies act as a scaffold for recruitment of the ULK complex to autophagic structures. This is supported by our data that the ULK1 LIR motif is required for ULK1 retention on GFP-DFCP1-positive membranes. Importantly, we show that knockdown of GABARAP specifically attenuates ULK1 activation: LC3B, GABARAPL1, and GATE-16 do not activate ULK1. In contrast, in yeast (Kraft et al., 2012) the Atg1 kinase is delivered to the vacuole via Atg8 to act as a brake on autophagy, and the Atg8-Atg1 interaction does not modulate kinase activity. The ULK1 association with GABARAP is constitutive and not regulated by starvation or lipidation, thus, the large non-lipidated pool of GABARAP at the centrosome may bind and prime ULK1 activity. In support of this, ULK1 activation by GABARAP, but not LC3B, requires the LIR motif but does not require lipidation. Thus, LIR motifs can be regulatory elements, for example, LIR-containing proteins displace Atg12–5-16 from Atg8–PE (Kaufmann et al., 2014). In the absence of WAC, aberrant GABARAP tethering to GM130 disrupts the autophagy-contributing centrosomal GABARAP reservoir and hence reduces GABARAP contribution to autophagosome formation and GABARAP-mediated ULK1 activation.

It has been hypothesized that the LIR-dependent binding of the ULK complex to LC3 and GABARAP proteins functions in the formation and maturation of autophagosomes, however the mechanism is unknown (Alemu et al., 2012). Supporting this idea, overexpression of an ULK1 LIR mutant results in the accumulation of WIPI2 puncta, suggesting autophagy has been stalled (Kraft et al., 2012). Similarly, knockdown of the GABARAP family causes accumulation of ATG5 and ATG16L1 puncta, suggesting autophagy is inhibited downstream of ATG12–5-16L1 complex recruitment (Weidberg et al., 2010).

Based on our data and existing literature, we propose a working model to explain the non-hierarchical (post-initiation) GABARAP-ULK1 function. Subsequent to activation of the BECLIN 1 complex by ULK1 (Russell et al., 2013), a pool of PtdIns(3)P is formed at the omegasome. This PtdIns(3)P recruits WIPI2, which recruits the ATG12–5-16L1 complex (Dooley et al., 2014). ATG16L1 can simultaneously bind WIPI2B and FIP200; FIP200 binding is downstream of WIPI2B and not required for LC3-lipidation (Dooley et al., 2014). Thus, recruitment of FIP200 (and the ULK complex) to the phagophore by ATG16L1 is downstream of PtdIns(3)P- (Karanasios et al., 2013), WIPI2B-, and ATG12–5-16L1-driven lipidation of the LC3 and GABARAP family (see Figure S7G). We hypothesize that the constitutive GABARAP-ULK1 complex, derived from either the centrosome or the cytosol, would be recruited to the phagophore through ATG16L1 binding. The GABARAP-ULK1 complex may also be stabilized by lipidation. GABARAP association maintains ULK1 activation and substrate phosphorylation during the final stages of phagophore formation, until the ULK1 complex dissociates (Karanasios et al., 2013) and the phagophore closes.

(B) Quantification of fluorescence intensity from video in (A). PC region marked by yellow arrow and distal region marked by magenta arrowhead in (A). Intensity 5 s prior to PC moment is set to 1 for normalization.

(C) Confocal microscopy performed on cell from video in (A). After imaging, cells were fixed and stained for LC3 and γ-tubulin. Arrows and arrowheads correspond to structures shown in (A). Scale bars, 10 μm.

(D) EosFP-GABARAP as in (A). PC moment is set to 0 s. Yellow box (top), photoconverted region. Yellow box (bottom), distal region. Scale bar, 10 μm.

(E) Quantification of intensities from boxed regions in (D). Intensity 60 s prior to PC moment is set to 1 for normalization.

(F) EosFP-GABARAP expressing cells in FM with nocodazole for 2 hr, then incubated in EBSS with nocodazole and imaged. Images captured every 3 s. Blue arrows track defined puncta. Scale bars, 20 μm.

(G) Confocal microscopy performed on cells from video in (F). After imaging, cells were fixed and stained for GM130. Arrows correspond to structures shown in (F). Scale bars, 20 μm.

(H) HEK293A cells were treated with RF, WAC, or GM130 siRNAs for 72 hr and incubated with full medium (Fed) or EBSS (Starved) for 2 hr. Scale bars, 50 μm. See also Figure S6 and Movies S1 and S2.

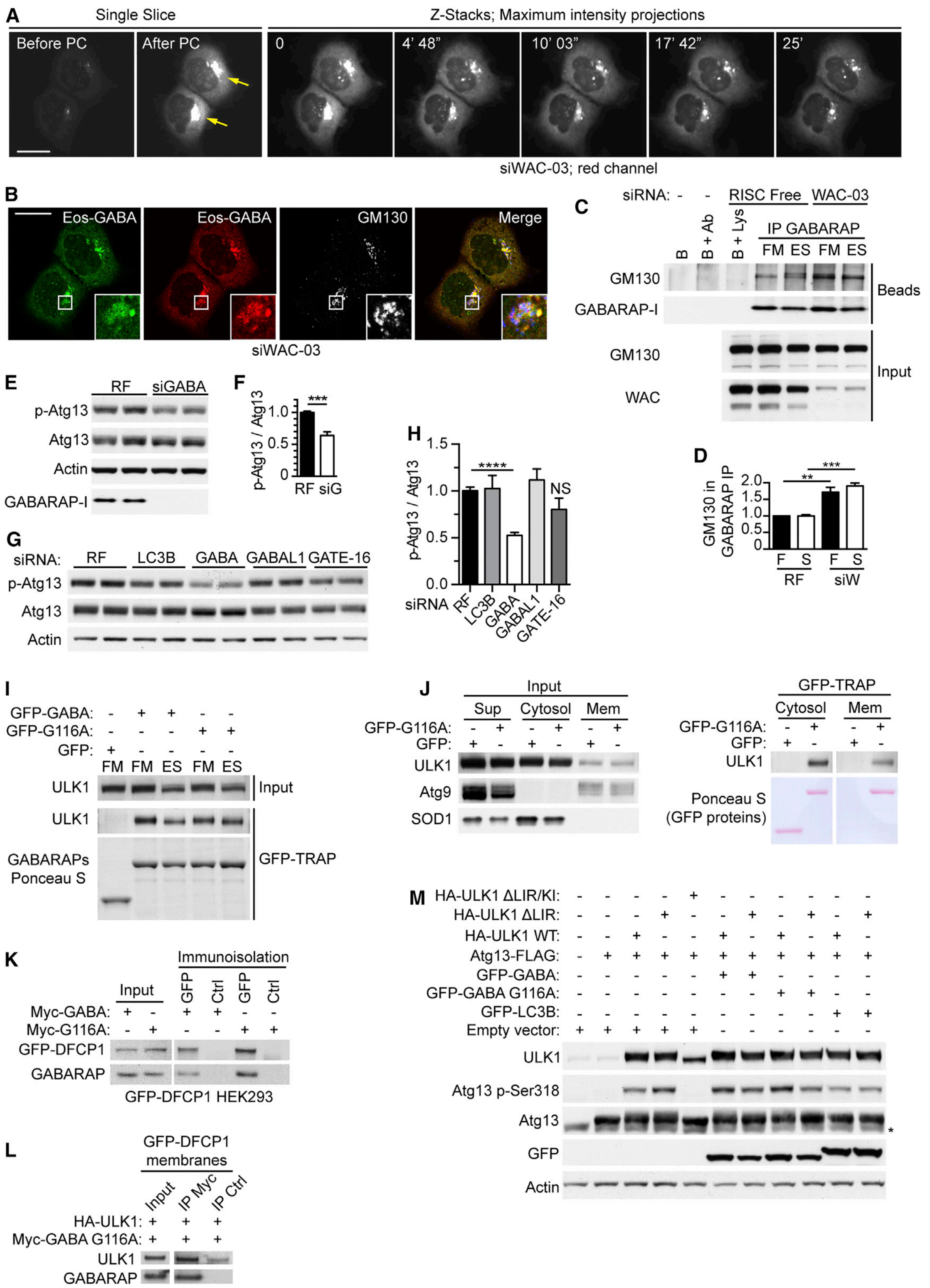

Figure 7. WAC Inhibits GM130 Binding of GABARAP and GABARAP-Mediated ULK1 Activation

(A) EosFP-GABARAP expressed in HEK293A cells treated with WAC siRNA for 72 hr. Live-cell imaging under starvation conditions. During photoconversion (PC), images from a single z slice were acquired every 0.4 s. After PC, z stacks were acquired every 13 s. Yellow arrows denote PC regions. Scale bars, 20 μm.

(B) After time-lapse imaging, cells shown in (A) were fixed and stained for GM130. Scale bars, 20 μm.

(legend continued on next page)

EXPERIMENTAL PROCEDURES

Cell Culture and Reagents
Cell lines, transfection protocols, siRNA, DNA constructs, primers, and antibodies are detailed in the Supplemental Experimental Procedures.

Microarray Studies
Whole-genome gene expression analysis was performed by the Genome Centre at Barts and the London School of Medicine and Dentistry, Queen Mary University of London (London, UK) using the Illumina human HT-12 v4 beadchip. Gene expression data were analyzed as described in the Supplemental Experimental Procedures.

Protein Complex Purification and Mass Spectrometry
Protein complexes were pulled down from fed cells via a GFP tag using GFP-TRAP beads (ChromoTek) or by immunoprecipitation of WAC. Proteins were resolved by SDS-PAGE, fixed, and stained with GelCode, and full lanes were cut into slices for tryptic digestion and mass spectrometry analysis. For more details, see Supplemental Experimental Procedures.

ACCESSION NUMBERS

The accession number for the microarray data reported in this paper is GEO: GSE66475.

SUPPLEMENTAL INFORMATION

Supplemental Information includes Supplemental Experimental Procedures, seven figures, one table, and three movies and can be found with this article online at http://dx.doi.org/10.1016/j.molcel.2015.11.018.

ACKNOWLEDGMENTS

We thank Terje Johansen (University of Tromsø) and Anne Simonsen (University of Oslo) for reagents; Mark Petalcorin (The Francis Crick Institute) for advice on BAC technology; Hisao Kondo (Kyushu University, Japan) for anti-WAC antibody and advice on staining; Hesso Farhan (University of Konstanz), Sean Munro (MRC Laboratory of Molecular Biology), Jesper Svejstrup (The Francis Crick Institute), Angelika Barnekow (University of Munster), Martin Lowe (University of Manchester), and Joachim Seemann (UTSW) for constructs; Almut Schulze (University of Wurzburg) for help in the design of Microarray analysis; Christopher Lamb for reading the manuscript; and the rest of the Tooze lab for support and advice. This work was supported by Cancer Research UK and the Francis Crick Institute, which receives its core funding from Cancer Research UK, the UK Medical Research Council, and the Wellcome Trust.

Received: May 4, 2015
Revised: August 18, 2015
Accepted: November 12, 2015
Published: December 17, 2015

REFERENCES

Abada, A., and Elazar, Z. (2014). Getting ready for building: signaling and autophagosome biogenesis. EMBO Rep. *15*, 839–852.

Alemu, E.A., Lamark, T., Torgersen, K.M., Birgisdottir, A.B., Larsen, K.B., Jain, A., Olsvik, H., Øvervatn, A., Kirkin, V., and Johansen, T. (2012). ATG8 family proteins act as scaffolds for assembly of the ULK complex: sequence requirements for LC3-interacting region (LIR) motifs. J. Biol. Chem. *287*, 39275–39290.

Alers, S., Löffler, A.S., Wesselborg, S., and Stork, B. (2012). Role of AMPK-mTOR-Ulk1/2 in the regulation of autophagy: cross talk, shortcuts, and feedbacks. Mol. Cell. Biol. *32*, 2–11.

Baschieri, F., Confalonieri, S., Bertalot, G., Di Fiore, P.P., Dietmaier, W., Leist, M., Crespo, P., Macara, I.G., and Farhan, H. (2014). Spatial control of Cdc42 signalling by a GM130-RasGRF complex regulates polarity and tumorigenesis. Nat. Commun. *5*, 4839.

Behrends, C., Sowa, M.E., Gygi, S.P., and Harper, J.W. (2010). Network organization of the human autophagy system. Nature *466*, 68–76.

Betin, V.M., and Lane, J.D. (2009). Caspase cleavage of Atg4D stimulates GABARAP-L1 processing and triggers mitochondrial targeting and apoptosis. J. Cell Sci. *122*, 2554–2566.

Biazik, J., Ylä-Anttila, P., Vihinen, H., Jokitalo, E., and Eskelinen, E.L. (2015). Ultrastructural relationship of the phagophore with surrounding organelles. Autophagy *11*, 439–451.

Birgisdottir, A.B., Lamark, T., and Johansen, T. (2013). The LIR motif - crucial for selective autophagy. J. Cell Sci. *126*, 3237–3247.

Chan, E.Y. (2009). mTORC1 phosphorylates the ULK1-mAtg13-FIP200 autophagy regulatory complex. Sci. Signal. *2*, pe51–pe51.

Chang, S.-H., Hong, S.-H., Jiang, H.-L., Minai-Tehrani, A., Yu, K.-N., Lee, J.-H., Kim, J.-E., Shin, J.-Y., Kang, B., Park, S., et al. (2012). GOLGA2/GM130, cis-Golgi matrix protein, is a novel target of anticancer gene therapy. Mol. Ther. *20*, 2052–2063.

Cole, N.B., Sciaky, N., Marotta, A., Song, J., and Lippincott-Schwartz, J. (1996). Golgi dispersal during microtubule disruption: regeneration of Golgi stacks at peripheral endoplasmic reticulum exit sites. Mol. Biol. Cell *7*, 631–650.

Dooley, H.C., Razi, M., Polson, H.E., Girardin, S.E., Wilson, M.I., and Tooze, S.A. (2014). WIPI2 links LC3 conjugation with PI3P, autophagosome formation, and pathogen clearance by recruiting Atg12-5-16L1. Mol. Cell *55*, 238–252.

Füllgrabe, J., Klionsky, D.J., and Joseph, B. (2014). The return of the nucleus: transcriptional and epigenetic control of autophagy. Nat. Rev. Mol. Cell Biol. *15*, 65–74.

(C) HEK293A cells were treated with RF or WAC siRNA for 72 hr and incubated with full medium (F) or EBSS (S) for 2 hr followed by GABARAP immunoprecipitation and immunoblotting. B, beads; Ab, GABARAP antibody; Lys, lysate.
(D) Quantification of (C), Student's t test, $^{**}p \leq 0.01$. Mean ± SEM of n = 3.
(E) HEK293A cells treated with RF or GABARAP siRNA for 72 hr, incubated in EBSS for 2 hr followed by immunoblot. p-Atg13, pSer318.
(F) Quantification of (E), Student's t test, $^{***}p \leq 0.001$. Mean ± SEM of n = 3.
(G) HEK293A cells treated with RF, LC3B, GABARAP, GABARAPL1, or GATE-16 siRNAs for 72 hr before 2 hr incubation in EBSS and immunoblot. p-Atg13, pSer318.
(H) Quantification of (G), Student's t test, $^{****}p \leq 0.0001$. Mean ± SEM of n = 3.
(I) HEK293A cells expressing GFP, GFP-GABARAP, or GFP-GABARAP G116A were incubated in FM or ES for 2 hr prior to GFP-TRAP and immunoblot.
(J) HEK293A cells expressing GFP or GFP-GABARAP G116A were incubated in EBSS for 2 hr prior to subcellular fractionation, GFP-TRAP, and immunoblot. Atg9 marks the membrane fraction and SOD1 the cytosol.
(K) HEK293 cells stably expressing GFP-DFCP1 transfected with Myc-GABARAP or G116A mutant were starved for 2 hr in EBSS, and the GFP-DFCP1 compartment was immunoisolated prior to immunoblot. Flag M2 antibody was used as a control.
(L) The GFP-DFCP1 compartment was isolated from HEK293 cells co-expressing the indicated constructs and starved in EBSS for 2 hr. Solubilised membranes were subjected to immunoprecipitation with anti-Myc or anti-FLAG M2 control.
(M) HEK293A cells expressing indicated proteins analyzed by immunoblot. *, non-specific band. KI, kinase-inactive ULK1. Quantification shown in Figure S7F. See also Figure S7 and Movie S3. Excised lanes are indicated by a gap, and remaining lanes are from the same gel.

Guo, Y., Chang, C., Huang, R., Liu, B., Bao, L., and Liu, W. (2012). AP1 is essential for generation of autophagosomes from the trans-Golgi network. J. Cell Sci. *125*, 1706–1715.

Itoh, T., Fujita, N., Kanno, E., Yamamoto, A., Yoshimori, T., and Fukuda, M. (2008). Golgi-resident small GTPase Rab33B interacts with Atg16L and modulates autophagosome formation. Mol. Biol. Cell *19*, 2916–2925.

Karanasios, E., Stapleton, E., Manifava, M., Kaizuka, T., Mizushima, N., Walker, S.A., and Ktistakis, N.T. (2013). Dynamic association of the ULK1 complex with omegasomes during autophagy induction. J. Cell Sci. *126*, 5224–5238.

Karpiuk, O., Najafova, Z., Kramer, F., Hennion, M., Galonska, C., König, A., Snaidero, N., Vogel, T., Shchebet, A., Begus-Nahrmann, Y., et al. (2012). The histone H2B monoubiquitination regulatory pathway is required for differentiation of multipotent stem cells. Mol. Cell *46*, 705–713.

Kaufmann, A., Beier, V., Franquelim, H.G., and Wollert, T. (2014). Molecular mechanism of autophagic membrane-scaffold assembly and disassembly. Cell *156*, 469–481.

Kimura, S., Noda, T., and Yoshimori, T. (2008). Dynein-dependent movement of autophagosomes mediates efficient encounters with lysosomes. Cell Struct. Funct. *33*, 109–122.

Kittler, J.T., Rostaing, P., Schiavo, G., Fritschy, J.M., Olsen, R., Triller, A., and Moss, S.J. (2001). The subcellular distribution of GABARAP and its ability to interact with NSF suggest a role for this protein in the intracellular transport of GABA(A) receptors. Mol. Cell. Neurosci. *18*, 13–25.

Kodani, A., and Sütterlin, C. (2008). The Golgi protein GM130 regulates centrosome morphology and function. Mol. Biol. Cell *19*, 745–753.

Kodani, A., Kristensen, I., Huang, L., and Sütterlin, C. (2009). GM130-dependent control of Cdc42 activity at the Golgi regulates centrosome organization. Mol. Biol. Cell *20*, 1192–1200.

Kraft, C., Kijanska, M., Kalie, E., Siergiejuk, E., Lee, S.S., Semplicio, G., Stoffel, I., Brezovich, A., Verma, M., Hansmann, I., et al. (2012). Binding of the Atg1/ULK1 kinase to the ubiquitin-like protein Atg8 regulates autophagy. EMBO J. *31*, 3691–3703.

Lamb, C.A., Yoshimori, T., and Tooze, S.A. (2013). The autophagosome: origins unknown, biogenesis complex. Nat. Rev. Mol. Cell Biol. *14*, 759–774.

Marra, P., Maffucci, T., Daniele, T., Tullio, G.D., Ikehara, Y., Chan, E.K., Luini, A., Beznoussenko, G., Mironov, A., and De Matteis, M.A. (2001). The GM130 and GRASP65 Golgi proteins cycle through and define a subdomain of the intermediate compartment. Nat. Cell Biol. *3*, 1101–1113.

McKnight, N.C., Jefferies, H.B., Alemu, E.A., Saunders, R.E., Howell, M., Johansen, T., and Tooze, S.A. (2012). Genome-wide siRNA screen reveals amino acid starvation-induced autophagy requires SCOC and WAC. EMBO J. *31*, 1931–1946.

Middendorp, S., Küntziger, T., Abraham, Y., Holmes, S., Bordes, N., Paintrand, M., Paoletti, A., and Bornens, M. (2000). A role for centrin 3 in centrosome reproduction. J. Cell Biol. *148*, 405–416.

Nakamura, N. (2010). Emerging new roles of GM130, a cis-Golgi matrix protein, in higher order cell functions. J. Pharmacol. Sci. *112*, 255–264.

Nakamura, N., Rabouille, C., Watson, R., Nilsson, T., Hui, N., Slusarewicz, P., Kreis, T.E., and Warren, G. (1995). Characterization of a cis-Golgi matrix protein, GM130. J. Cell Biol. *131*, 1715–1726.

Nakamura, N., Lowe, M., Levine, T.P., Rabouille, C., and Warren, G. (1997). The vesicle docking protein p115 binds GM130, a cis-Golgi matrix protein, in a mitotically regulated manner. Cell *89*, 445–455.

Oakley, B.R. (2000). An abundance of tubulins. Trends Cell Biol. *10*, 537–542.

Okazaki, N., Yan, J., Yuasa, S., Ueno, T., Kominami, E., Masuho, Y., Koga, H., and Muramatsu, M. (2000). Interaction of the Unc-51-like kinase and microtubule-associated protein light chain 3 related proteins in the brain: possible role of vesicular transport in axonal elongation. Brain Res. Mol. Brain Res. *85*, 1–12.

Orsi, A., Razi, M., Dooley, H.C., Robinson, D., Weston, A.E., Collinson, L.M., and Tooze, S.A. (2012). Dynamic and transient interactions of Atg9 with autophagosomes, but not membrane integration, are required for autophagy. Mol. Biol. Cell *23*, 1860–1873.

Pankiv, S., Lamark, T., Bruun, J.A., Øvervatn, A., Bjørkøy, G., and Johansen, T. (2010). Nucleocytoplasmic shuttling of p62/SQSTM1 and its role in recruitment of nuclear polyubiquitinated proteins to promyelocytic leukemia bodies. J. Biol. Chem. *285*, 5941–5953.

Poser, I., Sarov, M., Hutchins, J.R., Hériché, J.K., Toyoda, Y., Pozniakovsky, A., Weigl, D., Nitzsche, A., Hegemann, B., Bird, A.W., et al. (2008). BAC TransgeneOmics: a high-throughput method for exploration of protein function in mammals. Nat. Methods *5*, 409–415.

Rios, R.M. (2014). The centrosome-Golgi apparatus nexus. Philos. Trans. R. Soc. Lond. B Biol. Sci. *369*, 20130462.

Russell, R.C., Tian, Y., Yuan, H., Park, H.W., Chang, Y.-Y., Kim, J., Kim, H., Neufeld, T.P., Dillin, A., and Guan, K.-L. (2013). ULK1 induces autophagy by phosphorylating Beclin-1 and activating VPS34 lipid kinase. Nat. Cell Biol. *15*, 741–750.

Sagiv, Y., Legesse-Miller, A., Porat, A., and Elazar, Z. (2000). GATE-16, a membrane transport modulator, interacts with NSF and the Golgi v-SNARE GOS-28. EMBO J. *19*, 1494–1504.

Shema, E., Tirosh, I., Aylon, Y., Huang, J., Ye, C., Moskovits, N., Raver-Shapira, N., Minsky, N., Pirngruber, J., Tarcic, G., et al. (2008). The histone H2B-specific ubiquitin ligase RNF20/hBRE1 acts as a putative tumor suppressor through selective regulation of gene expression. Genes Dev. *22*, 2664–2676.

Shoji-Kawata, S., Sumpter, R., Leveno, M., Campbell, G.R., Zou, Z., Kinch, L., Wilkins, A.D., Sun, Q., Pallauf, K., MacDuff, D., et al. (2013). Identification of a candidate therapeutic autophagy-inducing peptide. Nature *494*, 201–206.

Shpilka, T., Weidberg, H., Pietrokovski, S., and Elazar, Z. (2011). Atg8: an autophagy-related ubiquitin-like protein family. Genome Biol. *12*, 226.

Slobodkin, M.R., and Elazar, Z. (2013). The Atg8 family: multifunctional ubiquitin-like key regulators of autophagy. Essays Biochem. *55*, 51–64.

Stolz, A., Ernst, A., and Dikic, I. (2014). Cargo recognition and trafficking in selective autophagy. Nat. Cell Biol. *16*, 495–501.

Totsukawa, G., Kaneko, Y., Uchiyama, K., Toh, H., Tamura, K., and Kondo, H. (2011). VCIP135 deubiquitinase and its binding protein, WAC, in p97ATPase-mediated membrane fusion. EMBO J. *30*, 3581–3593.

Wang, H., and Olsen, R.W. (2000). Binding of the GABA(A) receptor-associated protein (GABARAP) to microtubules and microfilaments suggests involvement of the cytoskeleton in GABARAPGABA(A) receptor interaction. J. Neurochem. *75*, 644–655.

Wei, J.-H., Zhang, Z.C., Wynn, R.M., and Seemann, J. (2015). GM130 Regulates Golgi-Derived Spindle Assembly by Activating TPX2 and Capturing Microtubules. Cell *162*, 287–299.

Weidberg, H., Shvets, E., Shpilka, T., Shimron, F., Shinder, V., and Elazar, Z. (2010). LC3 and GATE-16/GABARAP subfamilies are both essential yet act differently in autophagosome biogenesis. EMBO J. *29*, 1792–1802.

Wiedenmann, J., Ivanchenko, S., Oswald, F., Schmitt, F., Röcker, C., Salih, A., Spindler, K.D., and Nienhaus, G.U. (2004). EosFP, a fluorescent marker protein with UV-inducible green-to-red fluorescence conversion. Proc. Natl. Acad. Sci. USA *101*, 15905–15910.

Wong, M., and Munro, S. (2014). Membrane trafficking. The specificity of vesicle traffic to the Golgi is encoded in the golgin coiled-coil proteins. Science *346*, 1256898.

Xu, G.M., and Arnaout, M.A. (2002). WAC, a novel WW domain-containing adapter with a coiled-coil region, is colocalized with splicing factor SC35. Genomics *79*, 87–94.

Young, A.R.J., Chan, E.Y.W., Hu, X.W., Köchl, R., Crawshaw, S.G., High, S., Hailey, D.W., Lippincott-Schwartz, J., and Tooze, S.A. (2006). Starvation and ULK1-dependent cycling of mammalian Atg9 between the TGN and endosomes. J. Cell Sci. *119*, 3888–3900.

Zhang, F., and Yu, X. (2011). WAC, a functional partner of RNF20/40, regulates histone H2B ubiquitination and gene transcription. Mol. Cell *41*, 384–397.

Molecular Cell, Volume *60*

Supplemental Information

Activation of ULK Kinase and Autophagy by GABARAP Trafficking from the Centrosome Is Regulated by WAC and GM130

Justin Joachim, Harold B.J. Jefferies, Minoo Razi, David Frith, Ambrosius P. Snijders, Probir Chakravarty, Delphine Judith, Sharon A. Tooze

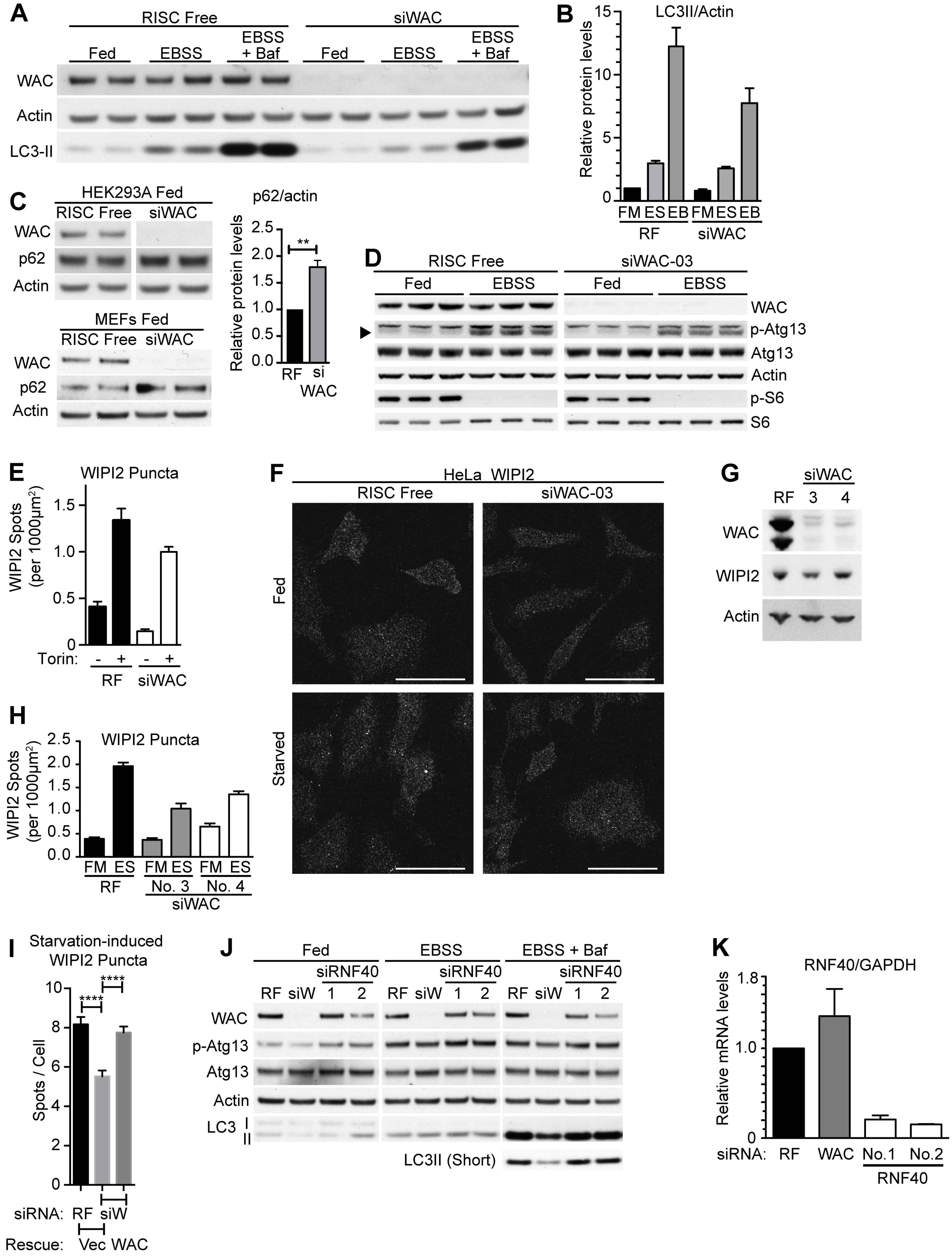

Supplementary Figure S1 Joachim et al.

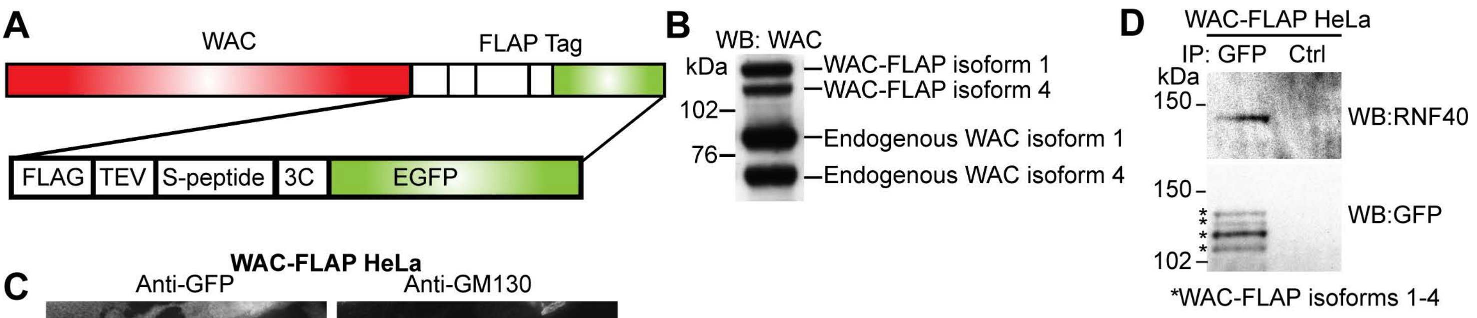

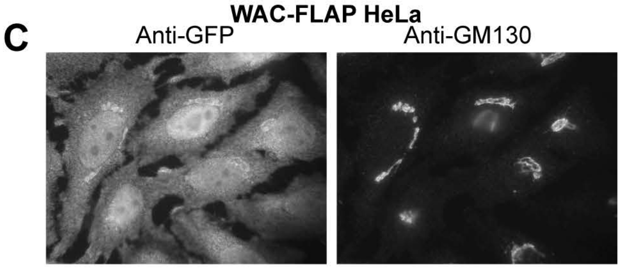

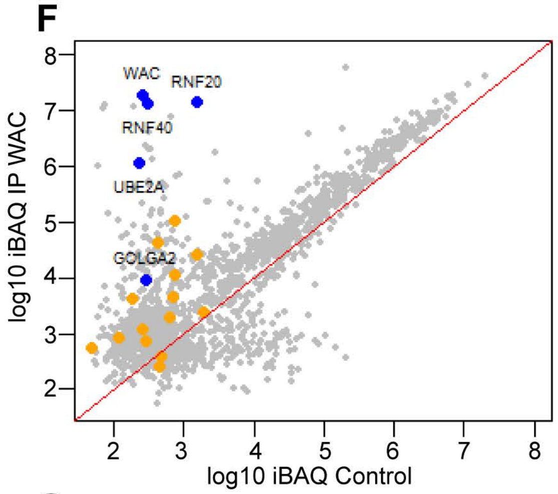

E

Gene names	PEP	Intensity	Unique peptides Ctrl	Unique peptides IP WAC	Unique peptides Ctrl	Unique peptides IP WAC-FLAP	Crapome Num of Expt. (found/total)
RNF20	0	1.05E+12	0	65	0	52	27 / 411
RNF40	0	9.78E+11	0	35	0	33	19 / 411
WAC (Q9BTA9)	0	3.51E+11	0	23	0	19	14 / 411
ERC1	5.30E-116	1.11E+08	0	21	0	1	44 / 411
GOLGA2	4.68E-28	9.31E+08	0	6	0	3	26 / 411
ADD2	1.89E-28	2.04E+10	0	5	0	1	12 / 411
PRPF40A	8.18E-09	8.03E+07	0	2	0	1	76 / 411
CYTSA;SPECC1L	9.85E-210	4.73E+10	0	1	0	20	17 / 411
WTAP	2.52E-214	1.94E+10	0	1	0	7	41 / 411
RBM15	6.52E-57	1.80E+10	0	1	0	9	40 / 411
UBE2A	2.29E-09	1.65E+10	0	1	0	1	
PSRC1	0.0014697	6.91E+09	0	1	0	2	
RAB1A;RAB1B;RAB1C	1.99E-08	6.39E+09	0	1	0	2	69 / 411
MYO18A	3.99E-34	5.42E+09	0	1	0	7	11 / 411
MCM4	4.76E-05	8.24E+08	0	1	0	2	113 / 411
EGFR;ERBB4;ERBB2	4.41E-05	7.47E+08	0	1	0	1	16 / 411
THOC2	1.96E-10	1.38E+08	0	1	0	1	64 / 411
MAP7D1;RPRC1	1.30E-07	1.38E+07	0	1	0	1	24 / 411
NACA;hCG_2016482	5.59E-07	2.65E+05	0	1	0	1	125 / 411

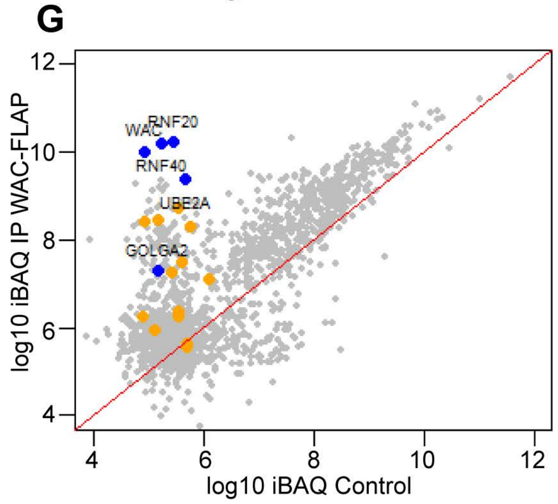

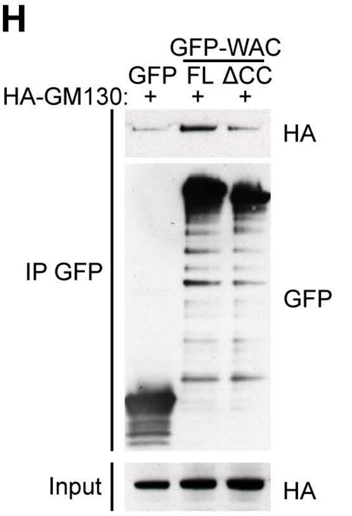

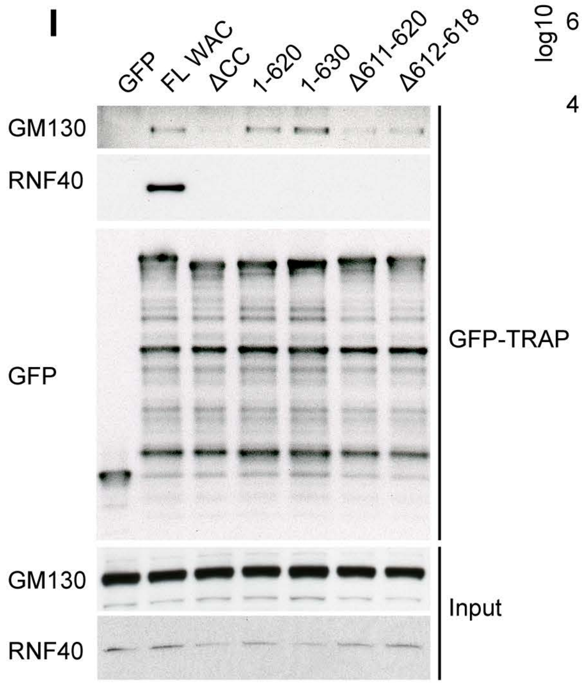

Supplementary Figure S2 Joachim et al.

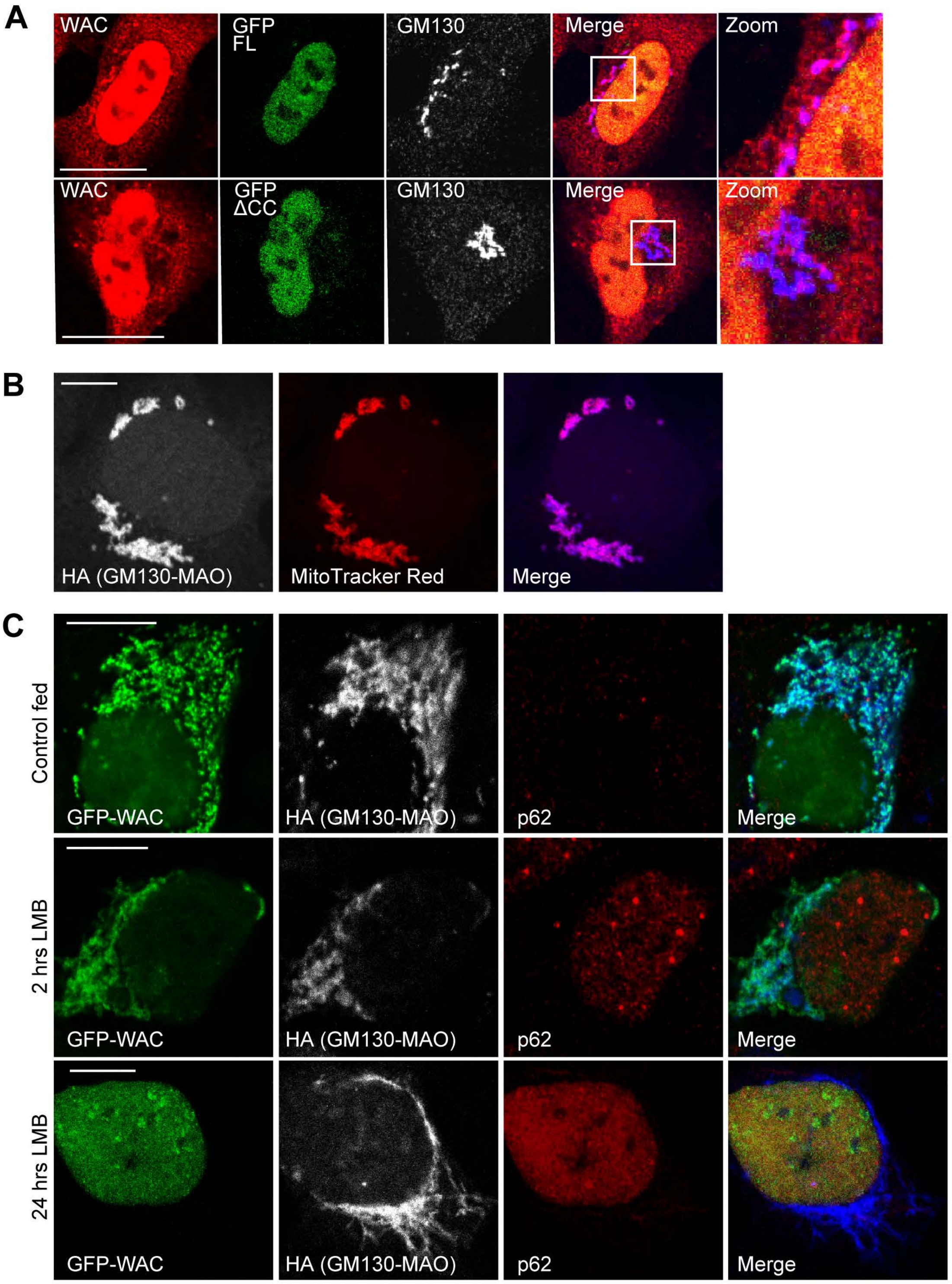

Supplementary Figure S3 Joachim et al.

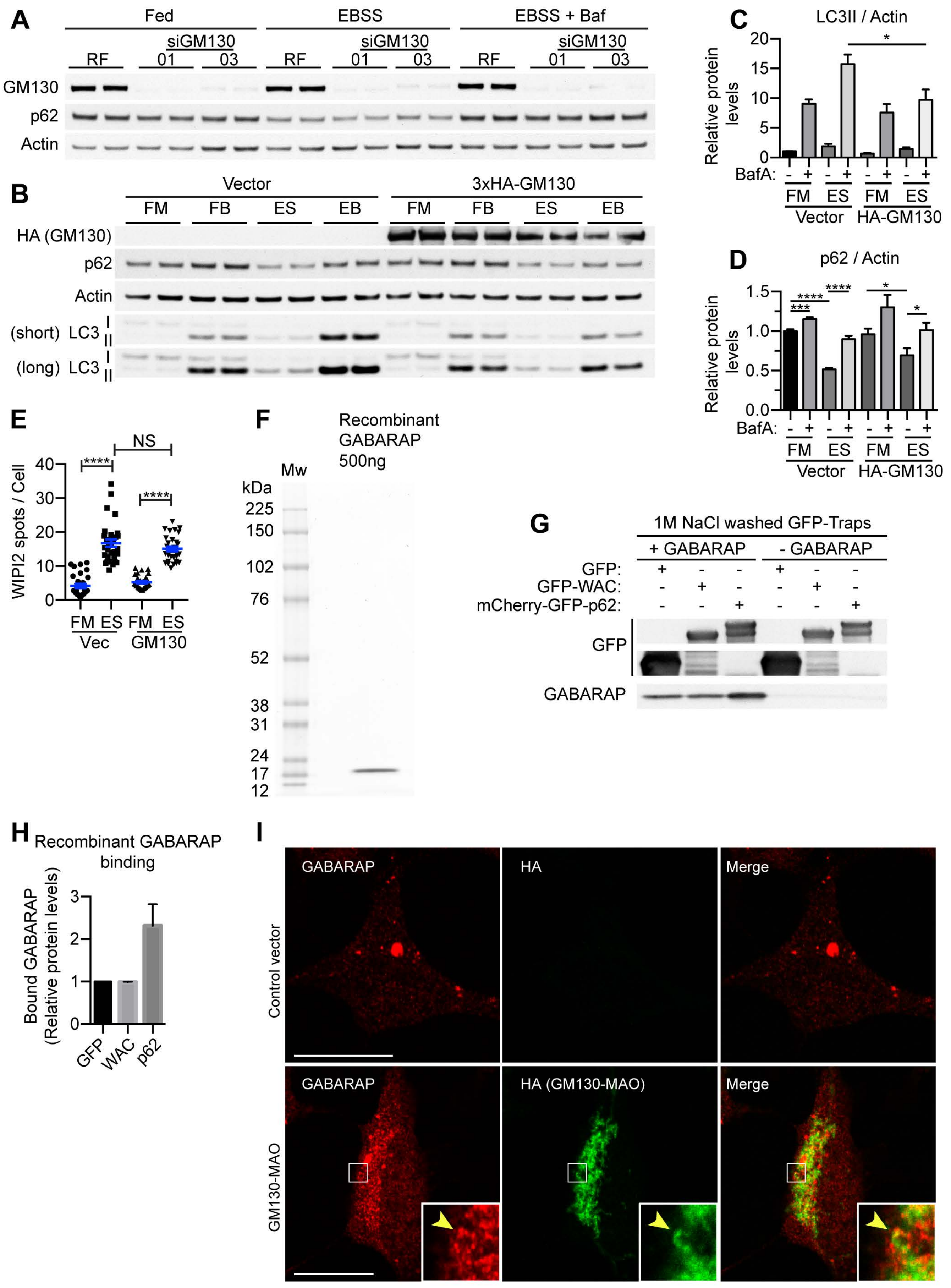

Supplementary Figure S4 Joachim et al.

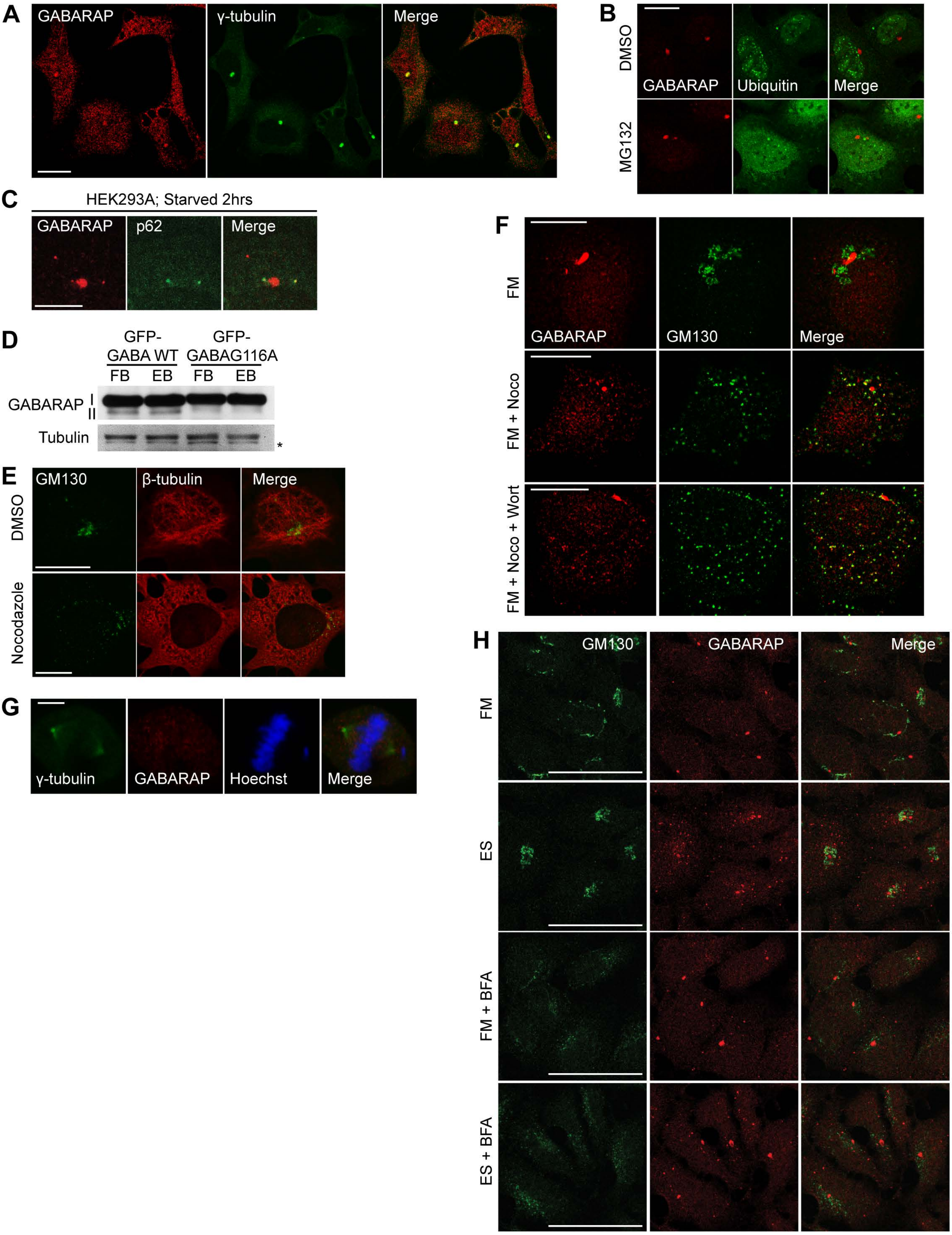

Supplementary Figure S5 Joachim et al.

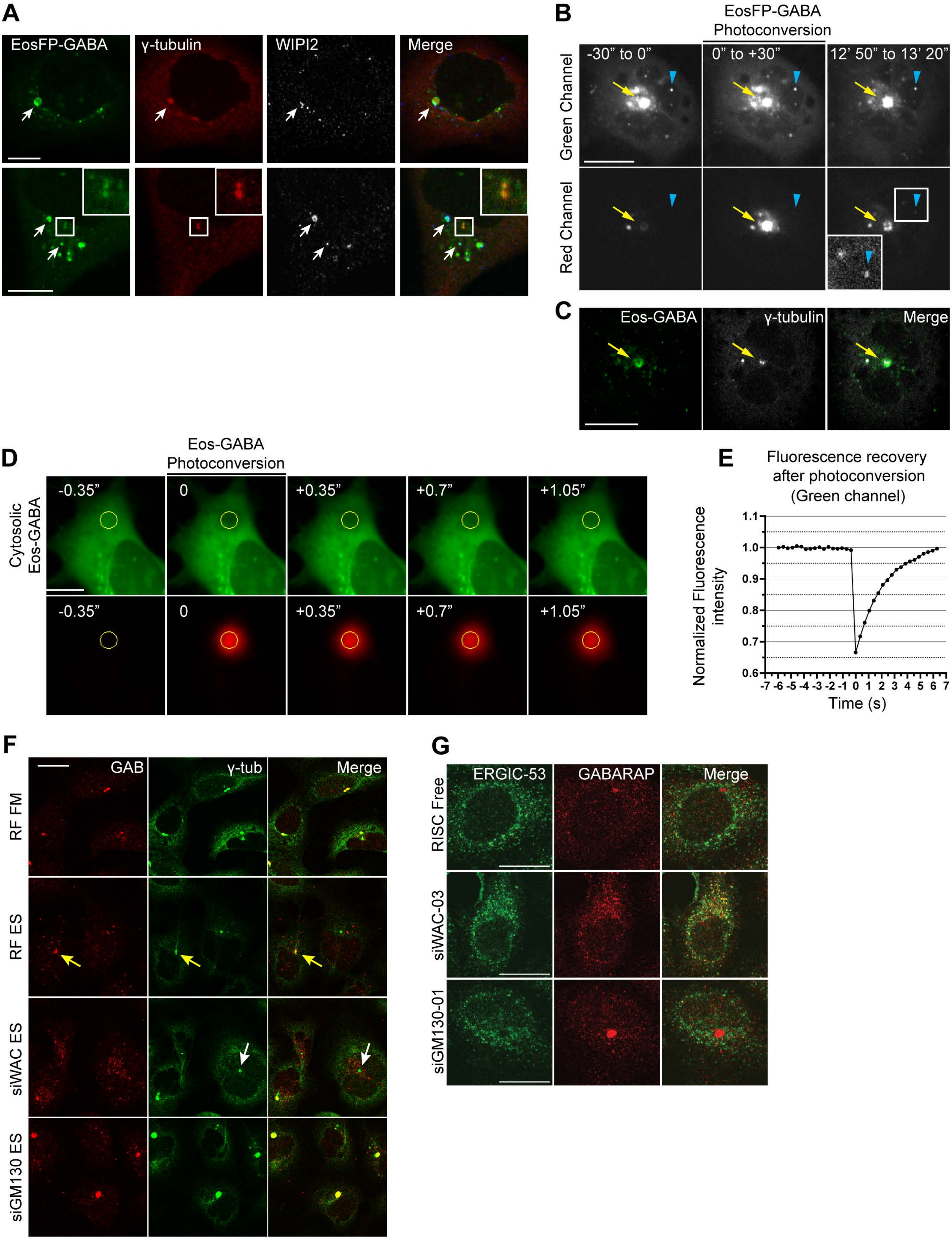

Supplementary Figure S6 Joachim et al.

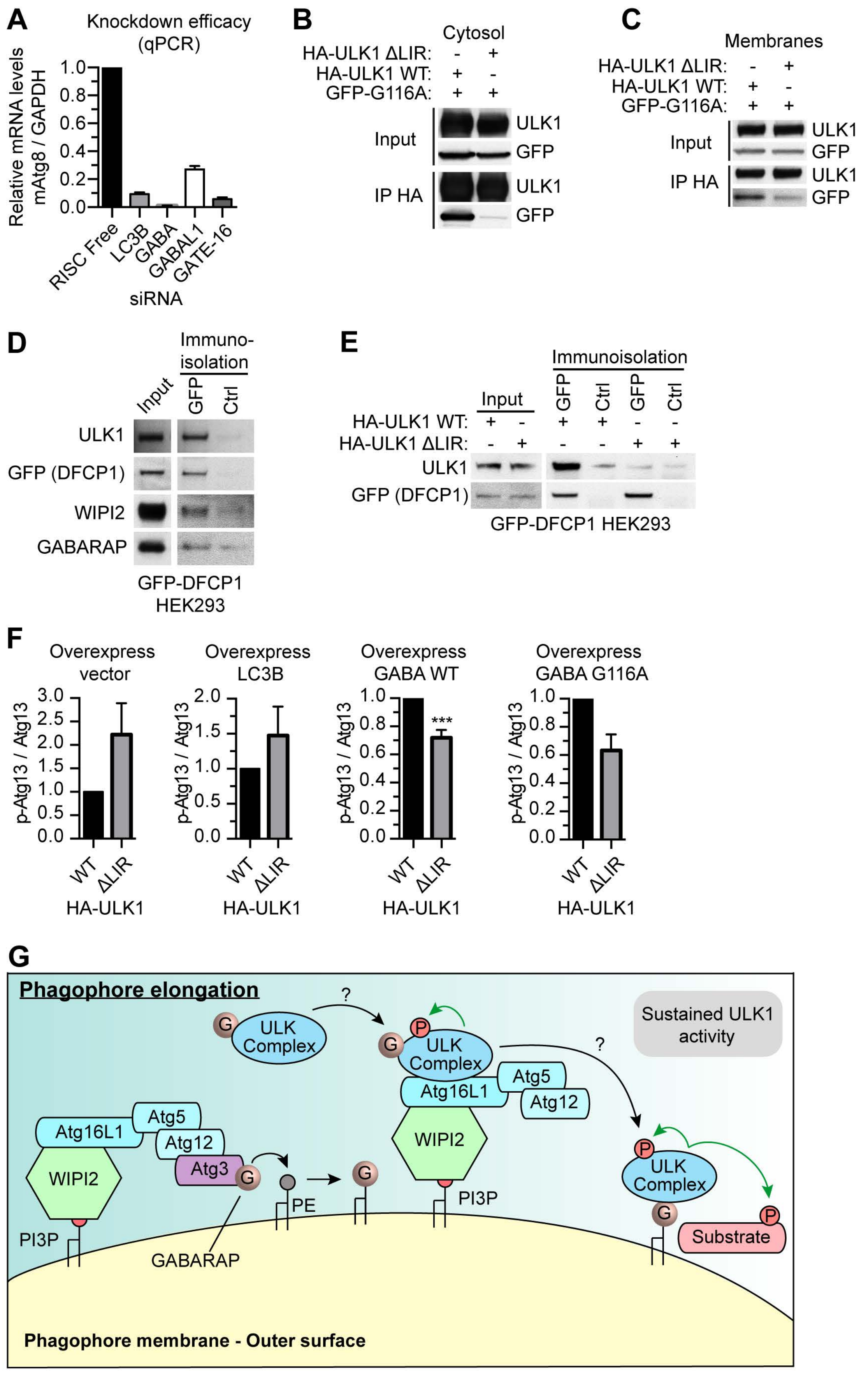

Supplementary Figure S7 Joachim et al.

Supplemental Figure Legends

Figure S1. WAC promotes autophagy in multiple cell lines and independently of RNF40, Related to Figure 1

A) HEK293A cells were treated for 72 hr with RF or WAC siRNA and then incubated in full medium (Fed), EBSS or EBSS + BafA1 for 2 hr before immunoblot analysis. B) Quantification of (A), mean ± SEM from 2 independent experiments. Full medium, FM; EBSS, ES; EBSS + BafA1, EB. C) HEK293A or MEF cells were treated for 72 hr with RF or WAC siRNA before immunoblot analysis. Graph shows quantification from HEK293A cells, statistics were performed using an unpaired Student's t test, *, $p \leq 0.05$. Mean ± SEM from 2 independent experiments. D) HEK293A cells were treated for 72 hr with RF or WAC siRNA and then incubated in full medium (Fed) or EBSS for 2 hr before immunoblot analysis. p-Atg13, anti-Atg13 pSer318; p-S6, anti-S6 pSer240/244. E) HEK293A cells were treated for 72 hr with RF or WAC siRNA and then incubated in full medium or full medium + Torin1 before analysis by confocal microscopy and quantification of WIPI2 puncta. Mean ± SEM from at least 80 cells per condition. F) HeLa cells were treated for 72 hr with RF or WAC siRNA (03 or 04) and then incubated in full medium (Fed) or EBSS (Starved) prior to analysis by either confocal microscopy (F) or immunoblot (G, Fed). H) Quantification of (F), mean ± SEM from at least 120 cells per condition. Full medium, FM; EBSS, ES. I) Vector (Vec) or Myc-WAC was expressed in HEK293A cells that were treated with RF or WAC (siW) siRNA for 72 hr and starved in EBSS for 2 hr prior to WIPI2 staining and confocal microscopy. Statistics were performed using an unpaired Student's t test, *, $p \leq 0.05$. Mean ± SEM from 3 independent experiments, >300 cells were counted per condition. J) HEK293A cells treated with RF, WAC or 2 different RNF40 siRNAs for 72 hr were incubated in full medium (fed), EBSS or EBSS + BafA1 for 2 hr prior to immunoblot analysis. K) HEK293A cells treated with RF, WAC or 2 different RNF40 siRNAs for 72 hr were maintained in full medium before analysis by qRT-PCR. mRNA levels of RNF40 were normalized to GAPDH. Mean ± SEM from duplicates. Excised lanes are indicated by a gap and remaining lanes are from the same gel.

Figure S2. Use of a bacterial artificial chromosome (BAC) to generate a WAC-FLAP HeLa cell line that mimics endogenous WAC and identifies GM130 as a WAC interactor, Related to Figure 2

A) Schematic showing WAC BAC containing the WAC gene (red) fused to a C-terminal FLAP tag. B) Immunoblot of WAC-FLAP HeLa cells showing tagged WAC isoform 1 and 4. C) Epifluorescence microscopy of WAC-FLAP HeLa cells. D) Lysates from WAC-FLAP HeLa cells were used for GFP-Trap pull-downs followed by immunoblot. Blocked agarose beads plus lysate was used as a negative control. E) MaxQuant protein groups table after applying filter criteria (see Supplemental Experimental Procedures). WAC and known interactors are highlighted in blue. GM130 (GOLGA2) is highlighted in orange. PEP = posterior error probability for protein identification. Control = beads and lysate without antibody. As a further quality parameter Crapome frequencies were added in the "Crapome Num of Expt. (found/total)" column, http://www.crapome.org. F) Intensity based absolute quantification (iBAQ) values are plotted for WAC interactors after immunoprecipitation of endogenous WAC from HEK293A cells versus the control sample (beads and lysate). Proteins in table (E) are highlighted as blue or orange dots. G) Intensity based absolute quantification (iBAQ) values are plotted for WAC interactors after immunoprecipitation of WAC-FLAP from HeLa cells versus the control sample (beads and lysate). Proteins in table (E) are highlighted as blue or orange dots. H) Lysates from HEK293A cells co-transfected with 3xHA-GM130 and EGFP, EGFP-WAC (FL) or aa1-610 (ΔCC) were used for GFP-Trap pull-down followed by immunoblot. I) Lysates from HEK293A cells transiently expressing EGFP, EGFP-WAC FL, ΔCC, 1-620, 1-630, Δ611-620, or Δ612-618 were used for GFP-Trap pull-down followed by immunoblot analysis.

Figure S3. Nuclear export of WAC is required to maintain cytoplasmic WAC-GM130 binding, Related to Figure 3

A) siRNA resistant EGFP-WAC FL or ΔCC were expressed in HEK293A cells treated with WAC siRNA for 72 hr before analysis by confocal microscopy using the indicated antibodies. Scale bars, 20 μm. B) HEK293A cells expressing GM130-ΔCterm-HA-MAO were incubated in full medium with MitoTracker Red for 2 hr before staining with anti-HA antibody and analysis by confocal microscopy. Scale bars, 10 μm. C) HEK293A cells expressing EGFP-WAC and GM130-ΔCterm-HA-MAO were incubated in full medium without or with Leptomycin B (LMB) for the indicated time before staining with HA and p62 antibodies and analysis by confocal microscopy. Scale bars, 10 μm.

Figure S4. GM130 overexpression suppresses LC3 lipidation during starvation and recruits GABARAP, Related to Figure 4

A) HEK293A cells treated with RF, GM130-01 or -03 siRNAs for 72 hr were incubated with full medium (fed), EBSS or EBSS + BafA for 2 hrs followed by immunoblot. The quantification of this experiment is shown in Fig. 4F. B) HEK293A cells expressing empty vector or 3xHA-GM130 were incubated in full medium (FM), full medium with BafA (FB), EBSS (ES), or EBSS with BafA (EB) for 2 hr prior to immunoblot. C) Quantification of (B) statistics were performed using an unpaired Student's t test, *, $p \leq 0.05$. Mean ± SEM from 3 independent experiments. D) Quantification of (B) statistics were performed using an unpaired Student's t test, *, $p \leq 0.05$. Mean ± SEM from 5 independent experiments. E) HEK293A cells expressing empty vector or 3xHA-GM130 were incubated in full medium (FM) or EBSS (ES) for 2 hr prior to confocal microscopy. F) Colloidal Coomassie stained SDS-PAGE gel showing purified untagged human GABARAP from *E. coli*. G) Lysates from HEK293A cells expressing the indicated GFP constructs were used for GFP-TRAP followed by salt washes and incubation with purified recombinant GABARAP from (F) and immunoblot. mCherry-GFP-p62 was used as a positive control for GABARAP binding. H) Quantification of (G) from 2 independent experiments. I) Vector or GM130-ΔCterm-HA-MAO were expressed in HEK293A cells followed by 2 hr incubation with EBSS, anti-HA and anti-GABARAP labelling and analysis by confocal microscopy. Arrows indicate co-localization. Scale bars, 20 μm.

Figure S5. Centrosomal GABARAP localization is dynamic and regulated by the cell cycle and microtubules but does not require the secretory pathway, Related to Figure 5

A) HEK293A cells were stained with sheep anti-GABARAP antibody and mouse anti-γ-tubulin before analysis by confocal microscopy. Scale bars, 20 μm. B) HEK293A cells incubated in DMSO or MG132 for 2 hr or C) in EBSS for 2 hr and assessed using the indicated antibodies. Scale bars, 10 μm. D) HEK293A cells expressing EGFP-GABARAP wild-type or the G116A mutant were incubated in full medium with BafA (FB) or EBSS with BafA (EB) for 2 hr prior to electrophoresis on an 8% SDS-PAGE gel and immunoblot. *, non-specific band. E) HEK293A cells were incubated in full medium with DMSO or nocodazole for 5 hr and assessed by confocal microscopy using the indicated antibodies. Scale bars, 20 μm. F) HEK293A cells were incubated in full medium (FM), FM with nocodazole or FM with nocodazole and wortmannin for 5 hr and assessed by confocal

microscopy using the indicated antibodies. Scale bars, 20 μm. G) Asynchronous HEK293A cells were incubated in full medium and metaphase cells were analysed by confocal microscopy. A single mitotic cell is shown. Scale bars, 5 μm. H) HEK293A cells were incubated in full medium (FM) or EBSS (ES) with or without Brefeldin A (BFA) for 2 hr and assessed by confocal microscopy using the indicated antibodies. Scale bars, 50 μm.

Figure S6. EosFP-GABARAP localizes to autophagosomes and the centrosome. Cytoplasmic EosFP-GABARAP translocates much more rapidly than the centrosomal species, Related to Figure 6

A) HEK293A cells expressing EosFP-GABARAP were incubated in EBSS for 2 hr before labelling with γ-tubulin and WIPI2 and analysis by confocal microscopy. Scale Bars, 10 μm. B) Live HEK293A cells expressing EosFP-GABARAP were starved in EBSS and imaged every 5 s using a swept field confocal microscope. Photoconversion (PC) was performed with localized pulses of 405nm light. PC moment is set to 0 s. Yellow arrow indicates photoconverted region. Blue arrow shows defined punctum. To reduce noise, 6 sequential images, equal to 30 s timeframe, were averaged for each time period shown. Scale bar, 20 μm. Inset has been contrast adjusted for clarity. C) Confocal microscopy performed on cell from (B). After time-lapse imaging, cells were fixed and stained for γ-tubulin. Yellow arrow indicates the same structure as in (B). Scale bar, 20 μm. D) HEK293A cells expressing high levels of cytosolic EosFP-GABARAP were starved in EBSS and imaged every 0.35 s using a swept field confocal microscope. Photoconversion (PC) was performed with 405nm light. PC moment is set to 0 s. Scale bar, 10 μm. E) Graph shows quantification of fluorescence intensity from yellow circle in images (D), for green channel only. Intensity 6 s prior to PA moment is set to 1 for normalization. F) HEK293A cells were treated with RF, WAC or GM130 siRNAs for 72 hr, incubated with full medium (FM) or EBSS (ES) for 2 hr and analyzed by confocal microscopy. Scale bars, 20 μm. Yellow arrows show GABARAP on the centrosome, white arrows show GABARAP dissociation from the centrosome. G) HEK293A cells were treated with RF, WAC or GM130 siRNAs for 72 hr, incubated with EBSS for 2 hr and analyzed by confocal microscopy. Scale bars, 20 μm.

Figure S7. GABARAP drives LIR-dependent ULK1 activation independently of lipidation and is present on DFCP1 positive membranes, Related to Figure 7

A) HEK293A cells were treated with RF, LC3B, GABARAP, GABARAPL1 or GATE-16 siRNA for 72 hr prior to analysis by qRT-PCR. Expression of each transcript is normalised to RF. n=3 independent experiments that are matched with 3 experiments from Fig. 7H. B) HEK293A cells expressing the indicated constructs were incubated in EBSS for 2 hr and subjected to subcellular fractionation and the cytosol was used for immunoprecipitation followed by immunoblot. C) HEK293A cells expressing the indicated constructs were incubated in EBSS for 2 hr and subjected to subcellular fractionation and the membranes were used for immunoprecipitation followed by immunoblot. D) HEK293 cells stably expressing GFP-DFCP1 were incubated in EBSS for 2 hr before homogenization and immunoisolation of the GFP-DFCP1 compartment with anti-GFP or anti-FLAG M2 control and immunoblot. E) HEK293 cells stably expressing GFP-DFCP1 and the indicated ULK1 constructs were incubated in EBSS for 2 hr before homogenization and immunoisolation of the GFP-DFCP1 compartment with anti-GFP or anti-FLAG M2 control and immunoblot. F) Quantifications of Fig. 7M, overexpress vector, 4 experiments; overexpress LC3B, 3 experiments; overexpress GABARAP, 5 experiments; overexpress G116A, 2 experiments. Statistics were performed using an unpaired Student's t test, ***, $p \leq 0.001$. G) Model is explained in the discussion. GABARAP promotes ULK1 activity and this requires the LIR motif of ULK1. As GABARAP acts during the membrane expansion/closure stage, this could maintain ULK1 activation past the early initiation stage of autophagy.

Table S1. Overlapping genes and pathways regulated by WAC, RNF20 and RNF40 knockdown, Related to Figure 1

Movie S1. Centrosomal GABARAP contributes to autophagosome formation, Related to Figure 6

Live cell imaging of a HEK293A cell expressing EosFP-GABARAP. Cells were washed into EBSS, imaged and photoconverted at 37°C with 10% CO_2 using a Nikon Eclipse Ti Swept Field Confocal microscope. Arrows mark the structures shown in Fig. 6A. Yellow arrow shows photoconverted centrosomal GABARAP. Blue arrow shows GABARAP punctum acquiring centrosomal GABARAP. Movie spans 25 mins played at 5 fps with 1 frame equal to 30 s. Note: first 5 frames are equal to 5 x 1 s images before photoactivation.

Movie S2. GABARAP positive autophagosomes are highly mobile and make transient interactions with the centrosome, Related to Figure 6

Live cell imaging of a HEK293A cell expressing EosFP-GABARAP. Cells were washed into EBSS, imaged and photoconverted at 37°C with 10% CO_2 using a Nikon Eclipse Ti Swept Field Confocal microscope. Centrosomal EosFP-GABARAP in the center of the cell is targeted for photoconversion. Blue arrow shows GABARAP punctum making contact with centrosomal GABARAP before leaving in a different direction. Movie spans 26 mins played at 10 fps with 1 frame equal to 5 s. Note: first 12 frames span the 60 s before photoactivation.

Movie S3. Depletion of WAC retains GABARAP on the Golgi where it becomes immobile and does not make autophagosomes, Related to Figure 7A

Live cell imaging of HEK293A cells depleted of WAC expressing EosFP-GABARAP. Cells were washed into EBSS, imaged and photoconverted at 37°C with 10% CO_2 using a Nikon Eclipse Ti Swept Field Confocal microscope. Arrows mark the structures shown in Fig. 7E. Golgi-localized GABARAP (yellow arrows) was targeted for photoconversion. Movie spans 25 mins played at 11.5 fps with 1 frame equal to 13.1 s. Maximum intensity projections of z-stacks are shown.

Extended Experimental Procedures

Cell culture and reagents

HEK293A, U2OS, RPE-1, HCT116, MEF, HEK293 GFP-DFCP1 and HeLa cells and their derivatives were grown in full medium: DMEM supplemented with 10% fetal calf serum and 4 mM L-glutamine. To induce autophagy, cells were washed 3 times with Earle's balanced salt solution (EBSS) and incubated in EBSS for two hours, unless otherwise stated. Where indicated, cells were treated with: 100 nM Bafilomycin A1 (Calbiochem), 100 nM Wortmannin (Calbiochem), 10 µg/ml Brefeldin A (Sigma), 100 nM Torin1 (Cayman Chemical), 50 µM Nocodazole (Sigma) or 20 ng/ml Leptomycin B (Sigma) for the specified time. HEK293 Flp-In T-Rex GFP-GABARAP cells were maintained in full medium + 200 µg/ml Hygromycin B + 5 µg/ml Blasticidin and induced for 24 hr with 1 µg/ml tetracycline in full medium to express GFP-GABARAP. HEK293 Flp-In T-Rex GFP-GABARAP cells were a kind gift from Anne Simonsen, (University of Oslo, Norway). The HEK293 GFP-DFCP1 stably expressing cells were a gift from N. Ktistakis (clone 201) (Axe et al., 2008) and maintained in the presence of G418 at µg/ml.

HeLa cell line stably expressing WAC-FLAP was established by transient transfection with a BAC (CTD-2309I17) containing the human WAC gene. The WAC BAC was modified by homologous recombination to contain a C-terminal FLAP tag (Poser et al., 2008). Transfection was carried out with Effectene (Qiagen) according to the method in (Poser et al., 2008). After transfection cells were selected with G418 (800 µg/ml) and cloned by FACs sorting for EGFP expression before being maintained in the presence of G418 at 400 µg/ml.

Lipofectamine 2000 (Life Technologies) was used for transient transfection of HEK293A cells according to the manufacturer's instructions. For rescue of autophagy with GFP-WAC and analysis by immunoblot, transfection was carried out with Lipofectamine 3000 (Life Technologies) according to the manufacturer's instructions. DNA plasmids were used at a concentration of 1 µg/mL of transfection mix. Where indicated pcDNA 3.1 (+) was used as an empty vector control.

For RNAi of HEK293A, MEF and HeLa cells, cells were transfected with the relevant siRNA oligo using Lipofectamine 2000 (Life Technologies). Cells were harvested 72 hr after transfection. For RNAi of GABARAP, cells were transfected with siRNA on day 1 and also day 2 of the procedure. Final concentration of siRNA oligos was 37.5 nM. siRNA oligos used (Dharmacon): D-001220-01 (RISC-Free, control), D-013325-02 (WAC-02 for MEFs), D-013325-03 (WAC-03), D-013325-04 (WAC-04), D-017282-01 (GM130-01), D-017282-03 (GM130-03), D-006913-01 (RNF40-01), D-006913-02 (RNF40-02), D-007027-01 (RNF20-01), D-007027-02 (RNF20-02), D-007027-03 (RNF20-03), D-007027-04 (RNF20-04), D-005049-04 (ULK1), D-012368-02 (GABARAP-02), D-012368-03 (GABARAP-03), D-012846-01 (LC3B), D-014715-02 (GABARAPL1), D-006853-03 (GATE-16). Unless otherwise specified, the siRNAs used for WAC, GM130 and GABARAP were -03, -01 and -02 respectively.

Knockdown and rescue experiments for Immunofluorescence were performed by transfection with siRNA on day 1 and transfection with DNA on day 3, as specified above. Rescue assay was carried out 24 hr after DNA transfection.

Human WAC BAC CTD-2309I17 was purchased from Life Technologies, pSC101-BAD-gbaAtet (for Red/ET recombination) was purchased from gene bridges, R6Kamp-FLAP for tagging the WAC BAC was a gift from Tony Hyman (Max Planck Institute or Molecular Cell Biology and Genetics, Dresden, Germany). Human Myc-WAC (pcDNA 3.1 (+)) and EGFP-WAC (pEGFP C2) truncations and Strep-Tag II-GM130 (Rat) (pcDNA 3.1 (+)) and EosFP-GABARAP (human) (pcDNA 3.1 (+)) were generated by PCR and cloned by ligation or using the In-fusion HD cloning kit (Clontech). Point mutations were generated by using QuikChange Multi Site-Directed Mutagenesis Kit (Agilent Technologies). Deletions were generated by Inverse PCR or PCR SOEing. HA-ULK1 and HA-ULK1 ΔLIR (D356A, F357A, P361A) were used in (Kraft et al., 2012). HA-ULK1 ΔLIR/KI contains the additional K49I kinase-inactivating mutation of human ULK1. Atg13-FLAG was used in (Chan et al., 2009). 3xHA-GM130 (pcDNA 3.1 (+)) (Rat) was a gift from Joachim Seemann (UT Southwestern Medical Center, Dallas, US), HA-GM130 truncations (Human) were a gift from Angelika Barnekow (University

of Münster, Germany), Rat GM130-ΔCterm-HA-MAO was a gift from Sean Munro (MRC laboratory of molecular biology, Cambridge, UK). EGFP-mAtg8 homologues and Myc-GABARAP (human) were a gift from Terje Johansen (UiT, The Arctic University of Norway, Tromsø). HA-VP35 (pcDNA 3.1/myc-His(-)) (Gantke et al., 2013) was a gift from Jesper Svejstrup (Francis Crick Institute, London, UK).

Mouse monoclonal antibodies: anti-ubiquitin (MBL, D058-3) anti-Centrin3 (Abcam, ab54531), anti-γ-tubulin ascites (Sigma, GTU-88, T6557), anti-p62 (BD Biosciences, 610832 & Abnova, H00008878-M01), anti-Myc (CRUK, 9E10), anti-FLAG M2 (Sigma), anti-p230/GOLGA4 (BD Biosciences, 611280), anti-ERGIC-53 (Enzo Life Sciences, ALX-804-602-C100), anti-βCOP (Sigma, M3A5, G2279), anti-GFP (CRUK, 3E1 & Roche, 11814460001), anti-GM130 (for IF) (BD Biosciences, 610822). Rabbit polyclonal antibodies: anti-β-tubulin (Abcam, ab6046), anti-GABARAP (Abgent, AP1821a), anti-HA (Covance, PRB-101P), anti-GFP (santa cruz, sc-8334), anti-RNF40 (Bethyl Laboratories, A300-718A), anti-WAC for WB (McKnight et al., 2012) for IF (Totsukawa et al., 2011), anti-WIPI2 (Polson et al., 2010), anti-Actin (Abcam, ab8227), anti-LC3 (Abcam, ab48394), anti-ULK1 (Santa Cruz, sc-33182), anti-ULK1 pSer757 (Cell Signaling, 6888), anti-Atg13 (Chan et al., 2009), anti-Atg13 pSer318 (Rockland, 600-401-C49), anti-phospho-S6 Ser240/244 (Cell Signaling, 2215). Rabbit monoclonal: anti-GM130 (Abcam, ab52649), anti-S6 (Cell Signaling, 2217). Hamster polyclonal: anti-Atg9 (Young et al., 2006). Sheep: anti-TGN46 (Serotec, AHP500G). Rat: anti-HA (for IF) (Roche, 3F10, 11867423001). Guinea pig polyclonal anti-p62 (for IF) (Progen, GP62-C). Antibodies were used at manufacturer's suggested concentrations. Secondary antibodies for IF, from Life Technologies unless otherwise specified, were anti-rabbit IgG Alexa Fluor 488, 555 and 647, anti-mouse IgG Alexa Fluor 488, 647 and 350, anti-guinea pig Alexa Fluor 555 and FITC (Santa Cruz), anti-rat Alexa Fluor 633 and 488, anti-sheep Alexa Fluor 488 and 647 and anti-hamster Cy3 (Jackson ImmunoResearch). HRP-conjugated secondary antibodies used for WB were from GE Healthcare.

Western Blotting

Cells were lysed in ice-cold TNTE buffer (20 mM Tris, pH 7.4, 150 mM NaCl, 0.5% w/v Triton X-100, 5 mM EDTA) containing EDTA-free Complete Protease Inhibitor cocktail (Roche). Lysates were cleared by centrifugation and resolved on NuPAGE®Bis-Tris 4–12% gels (Life Technologies) followed by transfer onto a PVDF membrane (Millipore). For WAC phosphorylation analysis, 25 µM Phos-Tag (NARD institute, AAL-107) 7.5% SDS-PAGE gels were prepared according to manufacturer's instructions. Following incubation with primary and secondary antibodies the blots were developed by enhanced chemiluminescence (GE Healthcare). Densitometry was performed with ImageJ software. For western blotting of GM130, primary antibody was diluted with SignalBoost Immunoreaction Enhancer Kit (Merck Millipore, 407207) and blots were developed with Luminata Crescendo Western HRP substrate (Merck Millipore).

Microarray studies

HEK293A cells were treated with RISC free, WAC, RNF40 or RNF20 siRNA pools (WAC-03 and 04; RNF40-01 and 02; RNF20-01, 02, 03 and 04) in triplicate and maintained in full medium, as described above. RNA was extracted using the RNeasy kit (Qiagen). RNA labelling was performed using the Ambion Total Prep Kit (Life Technologies).

Gene expression data was analyzed using Bioconductor 2.2 (http://bioconductor.org) running on R2.7.1 (http://www.R-project.org) Normalized probe set expression measures were calculated using log2 transformation and quantile normalization using the Lumi package (Du et al., 2008). All groups contained three independent samples except for RNF40, which had two independent samples after removal of one replicate for quality control.

To determine significant differences of expression in the three groups: siWAC, siRNF20 and siRNF40 relative to RISC free, a moderated Student's t-test was computed on a gene-by-gene basis using the empirical Bayes statistics in the Limma package (Ritchie et al., 2015). The resultant p-values were adjusted for multiple testing using the False Discovery Rate (FDR) Benjamini and Hochberg method, where any probe sets that exhibited a FDR

of less than 0.05 were called differentially expressed. No fold change thresholds were applied.

Differential genes from three comparisons were intersected to identify common genes (Fig. 1G and Table S1). The common genes (319) were further subdivided into two groups: those whose FC difference relative to RISC free were down regulated in all comparisons (183) and those whose FC difference relative to RISC free were up regulated in all comparisons (118). 18 genes were differentially regulated between knockdown of the 3 proteins and subtracted from our analysis.

Differentially expressed genes (301) were analyzed for enrichment of pathways, biological processes and transcription factor targets using gene sets from Metacore Pathway analysis tool (Thomson Reuters) using a hypergeometric distribution to determine enriched gene set using all genes on the Illumina array as the background. Pathways or processes that showed a FDR of less than 0.05 were called as enriched.

qRT-PCR

HEK293A cells were treated with the indicated siRNA and maintained in full medium. Total RNA was isolated using the RNeasy kit (Qiagen) and cDNA synthesis was performed with SuperScript II reverse transcriptase (Life Technologies). qRT-PCR was performed using the Fast SYBR Green Master Mix (Applied Biosystems, 4385612) and PCR products were detected by the 7500 FAST Real-Time PCR System (Applied Biosystems). The following primers were used to generate amplicons: forward (*5'-AAC AAC GGC AGG CTT GTG AAG ATG-3'*) and reverse (*5'-ATC GGA GAA GGG CTT CCA CAG TTT-3'*) primers for RNF40, forward (*5'-GAC CAC TTT GTC AAG CTC ATT TC-3'*) and reverse (*5'-CTC TCT TCC TCT TGT GCT CTT G-3'*) primers for GAPDH, forward (*5'- GCG AGA AGA TCC GAA AGA AA -3'*) and reverse (*5'- GAT CAG AAG GCA CCA GGT ATT T -3'*) primers for GABARAP, forward (*5'- TGG GCC AAC TGT ATG AGG A -3'*) and reverse (*5'- CTA CCC CCA AGT CCA GGT G -3'*) primers for GABARAPL1, forward (*5'- CCG TCG TTG TTG TTG TGC T -3'*) and reverse (*5'- CTC CAC GCA TCT GTG TTC C -3'*) primers for GATE-16, forward (*5'- GAG GAT CTT TAG GCC TGA G -3'*) and reverse (*5'- TTC TCA CAC AGC CCG TTT AC -3'*) primers for LC3B. The CT

values corresponding to target mRNA was normalized to that of GAPDH mRNA.

Protein complex purification and mass spectrometry

HEK293A or HeLa WAC-FLAP cells, maintained in full medium, were washed in PBS and lysed in TNTE buffer (20 mM Tris-HCl pH 7.4, 150 mM NaCl, 5 mM EDTA, 0.5 % Triton X-100, 1x Complete protease inhibitor (Roche), 1x PhosSTOP (Roche)) and the lysate clarified by centrifugation (16,100 x g, 15 min). Lysates from HeLa WAC-FLAP cells were incubated with GFP-TRAP® beads at $4^{o}C$ for 2 hr. Lysates from HEK293A cells were incubated with Rabbit anti-WAC bound to protein G sepharose (Sigma) at $4^{o}C$ for 2 hr. Pelleted beads were washed 3 times with TNTE buffer and eluted with 2x Laemmli sample buffer at $100^{o}C$ for 10 min.

Eluted proteins were separated by SDS-PAGE and 8 bands covering the entire lane were excised for each sample. In-gel trypsin digestion was performed using a Perkin Elmer Janus liquid handling system. Lyophilized peptide samples were dissolved in 15 µl of 0.1 % TFA and subjected to LC-MS analysis using a LTQ-Orbitrap instrument for data acquisition. Raw spectra were processed using the MaxQuant/Andromeda bioinformatics suite (Cox and Mann, 2008) and further analyzed in Perseus. Data was searched against a UniProt fasta database containing human sequences and intensity based absolute quantification (iBAQ) (Schwanhausser et al., 2011) was used for label free quantification. WAC, WAC-FLAP and control IPs (beads and lysate) were analyzed to screen for novel WAC interactors and filtered as follows. Only proteins that received iBAQ values in both experiments and not in the control IPs were considered candidates. The original dataset contained 1536 protein identifications and using the filter criteria the list was reduced to 20 candidates that were enriched in the WAC & WAC-FLAP pull-downs vs control conditions.

Immunoprecipitation

Cells were lysed using TNTE buffer (20 mM Tris-HCl pH 7.4, 150 mM NaCl, 5 mM EDTA, 0.5 % Triton X-100, 1x Complete protease inhibitor (Roche), 1x PhosSTOP (Roche)) and the clarified lysates used for

immunoprecipitation with either rabbit anti-WAC, rabbit anti-GABARAP or rat anti-HA for 2 hr at 4°C. Where indicated, species matched anti-GFP is used as a control IP. Antibodies were coupled to protein G sepharose (Sigma). Pelleted beads were washed 3 times with TNTE buffer and eluted with 2x Laemmli sample buffer at 100°C for 10 min before resolving by SDS-PAGE (4-12 % Bis-Tris NuPAGE gels, Life Technologies) and western blotting. GFP-tagged proteins were immunoprecipitated using GFP-TRAP® beads and HA-tagged proteins with anti-HA affinity matrix 3F10 (Roche), using the same buffer and protocol. During western blotting of IP experiments, TrueBlot® (Rockland) was used to reduce background from IgG. Where indicated immunoprecipitates where washed 5 x 1M NaCl TNTE buffer (20 mM Tris-HCl pH 7.4, 1M NaCl, 5 mM EDTA, 0.5 % Triton X-100, 1x Complete protease inhibitor (Roche)) and then washed 2 x TNTE buffer before addition of cell lysate. For dephosphorylation of GFP-WAC, washed GFP-TRAPs of GFP-WAC were incubated in 100 µL volume with 1 µL lambda phosphatase (NEB, P0753) with the accompanying manufacturer's buffer and 1mM MnCl2 at 30°C for 30 mins. As a control, samples were incubated with TNTE buffer instead. Lambda phosphatase was then removed by 2 x TNTE buffer washes and where indicated HEK293A cell lysates were incubated with GFP-TRAPs before washing and immunoblotting as above.

Immunoisolation of GFP-DFCP1-positive membranes

HEK293 cells stably expressing GFP-DFCP1 were treated with EBSS for 2 hr. Cells were then washed in ice cold PBS and harvested by centrifugation at 200 x g at 4°C. Pellets were resuspended using a cold isotonic buffer (20mM HEPES, pH 7.4; 250mM sucrose; 1mM EDTA) supplemented with EDTA-free Complete protease inhibitor cocktail (Roche). The resuspended pellet was then passed through a 27G needle for homogenization before clarification by centrifugation at 3000 x g at 4°C. Supernatants were used for incubation overnight at 4°C with mouse anti-FLAG M2 or mouse anti-GFP antibody protein A Dynabeads®. The GFP-DFCP1-positive membranes on the beads were then washed 3 times (Isotonic buffer supplemented with 75mM NaCl) and eluted with 2x laemmli sample buffer before resolving by SDS-PAGE and western blotting.

Where indicated, GFP-DFCP1 cells were transfected with the appropriated plasmid 24h prior isolation of GFP-DFCP1-positive membrane.

Immunoprecipitation from GFP-DFCP1-positive membranes was performed by solubilizing immunoisolated membrane using cold TNTE buffer (20 mM Tris-HCl pH 7.4, 150 mM NaCl, 5 mM EDTA, 0.5 % Triton X-100, 1x Complete protease inhibitor (Roche), 1x PhosSTOP (Roche)). The lysates then were used for immunoprecipitation with the indicated antibody protein A Dynabeads® for 2h at 4°C. Subsequently, beads were washed 3 times with TNTE buffer and eluted with laemmli sample buffer before resolving by SDS-PAGE and western blotting.

Subcellular fractionation

Cells were washed and pelleted in ice cold HEPES buffer (20 mM HEPES-KOH pH 7.5, 10 mM KCl, 2.5 mM MgOAc, 1mM EDTA) and then resuspended in HEPES buffer with sucrose (20 mM HEPES-KOH pH 7.5, 250 mM sucrose, 10 mM KCl, 2.5 mM MgOAc, 1mM EDTA, 1 mM DTT, 1X PhosSTOP (Roche), 1X Complete EDTA-free protease inhibitor cocktail (Roche)) and incubated on ice for 20 minutes. Cells were then homogenised by passing through a 27G needle and homogenization was monitored by Trypan Blue staining. Nuclei were removed by centrifugation at 5,000 rpm for 5 minutes at 4°C in an Eppendorf micro centrifuge, this was repeated and the supernatant was subjected to centrifugation at 112,500 x g for 1 hr at 4°C to obtain the membrane pellet and the cytosol supernatant.

1% Triton X-100 and 150 mM NaCl were added to the cytosol fractions which were then subjected to immunoprecipitation at 4°C with GFP-TRAP beads (Chromotek) or anti-HA affinity matrix clone 3F10 (Roche) followed by SDS-PAGE and western blotting. Immunoprecipitates were washed 3 x with ice cold TNTE buffer before analysis (20 mM Tris-HCl pH 7.4, 150 mM NaCl, 5 mM EDTA, 0.5 % Triton X-100, 1x Complete protease inhibitor (Roche), 1x PhosSTOP (Roche)).

For membrane pellets, an equivalent volume (to cytosol) of HEPES buffer with sucrose (with 1% Triton X-100 and 150 mM NaCl) was added, and the pellets were incubated at 37°C for 30 minutes to solubilise membranes that would be insoluble in Triton X-100 at 4°C. Any insoluble material was

cleared by centrifugation at 16,100 x g and the supernatants were subjected to immunoprecipitation at 37°C followed by SDS-PAGE and western blotting. Immunoprecipitates were washed 3 x with TNTE buffer warmed to 37°C before analysis.

Protein expression, purification, *in vitro* binding and competition

Human GST-WAC fusion proteins were cloned into pGEX-4T-2 and expressed in *E. coli* BL21-CodonPlus(DE3)-RIL (Agilent) cells in LB medium. Human GST-GABARAP pGEX-5X-1 was a gift from Zvulun Elazar (Weizmann Institute of Science, Israel). Expression was induced by addition of 1 mM IPTG at OD_{600} = 0.6 and cells were incubated at 37°C for 4 hr. Harvested cells were lysed using sonication on ice in a lysis buffer (PBSA + 1 % Triton X-100, supplemented with 1 x Complete protease inhibitor (Roche)) and the clarified supernatant was subsequently applied to Glutathione Sepharose 4B beads (GE Healthcare). After several washes with (PBSA + 1 % Triton X-100 + 500 mM NaCl supplemented with 1 x Complete protease inhibitor (Roche)), fusion protein-bound beads were used directly in GST pulldown assays. Purified untagged human GABARAP was a gift from Stephane Mouilleron, Francis Crick Institute, London, UK.

To produce recombinant untagged WAC aa320-647, the protein was expressed as above and bound to Glutathione Sepharose 4B beads. The immobilized protein was incubated with Thrombin protease (GE Healthcare) for 16 hr at room temperature in PBS to cleave off the GST tag and 2X Complete EDTA-free protease inhibitor cocktail (Roche) was added to the supernatant followed by analysis by SDS-PAGE and colloidal Coomassie staining.

Strep-Tag II-GM130 (Rat) was cloned into pcDNA 3.1(+) and co-expressed in HEK293A cells with HA-VP35 to boost protein expression (Gantke et al., 2013). Cells were lysed in TNTE buffer (20 mM Tris-HCl pH 7.4, 150 mM NaCl, 5 mM EDTA, 0.5 % Triton X-100, 1x Complete protease inhibitor (Roche), 1x PhosSTOP (Roche)). After clarification of lysates, lysates were incubated with magnetic Strep-Tactin beads (Qiagen) for 1 hr before 5x washes in stringent conditions (20 mM Tris-HCl pH 7.4, 1 M NaCl, 5 mM EDTA, 1 % Triton X-100, 1x Complete protease inhibitor (Roche), 1x

PhosSTOP (Roche)). Beads were incubated with elution buffer (TNTE + 10mM Biotin) for 15 mins on ice to elute Strep-Tag II-GM130. Purity of eluted protein was assessed by SDS-PAGE and colloidal coomassie staining.

For *in vitro* binding, soluble Strep-Tag II-GM130 was incubated with immobilized GST (20 µg), GST-GABARAP (20 µg), GST-WAC or GST-WAC ΔCC on glutathione beads for 2 hr at 4°C in TNTE buffer (GST-WAC) or 1.5 hr at 4°C in TNTE buffer supplemented with 10% (v/v) glycerol and 0.1% (w/v) BSA (GST and GST-GABARAP). Beads were then washed 3 x with TNTE before SDS-PAGE and western blotting. For competition, soluble Strep-Tag II-GM130 was incubated with recombinant WAC aa320-647 for 2 hr at 4°C in TNTE buffer supplemented with 10% (v/v) glycerol and 0.1% (w/v) BSA and this mixture was then incubated with GST or GST-GABARAP beads as above.

Confocal and epifluorescence microscopy

Cells were grown on coverslips, fixed with 3 % paraformaldehyde in PBS for 20 min before permeabilization with either 0.1 % saponin in PBS for 20 min (WAC & βCOP), 0.2 % Triton X-100 in PBS for 3 min (ERGIC-53) or room temperature methanol for 5 min (WIPI2 & GABARAP & other antibodies). Coverslips were then blocked in 5 % BSA in PBS (Roche) after methanol permeabilization, with 0.2 % gelatin in PBS after Triton X-100 permeabilization or with 0.1 % saponin + 1 % BSA + 0.2 % gelatin in PBS for 20 min. Coverslips were incubated with primary antibody in 1 % BSA in PBS or 0.2 % gelatin in PBS for 1 hr at room temperature. For WAC and βCOP staining, coverslips were incubated with primary antibody + 0.1 % saponin, 1 % BSA and 0.2 % gelatin in PBS overnight at 4°C. Coverslips were washed and incubated with secondary antibody in the same buffer as primary for 1 hr, before final washing with PBS and water. LC3 and WIPI2 puncta formation and centrosomal GABARAP intensity was quantified by Imaris image analysis software.

Live cell imaging

Live cell imaging was performed on HEK293A cells transiently expressing EosFP-GABARAP. Alternatively, cells were treated with WAC

siRNA for 48 hr prior to transfection with EosFP-GABARAP and imaging 24 hr later. Cells were washed 3 times into EBSS and maintained at 37°C with 10% CO_2 during imaging with a Nikon Eclipse Ti microscope. Photoconversion was performed with pulses of 405 nm light. Images were processed, and data analyzed, using the Fiji distribution of ImageJ. Where indicated, cells were pretreated with 50 μM Nocodazole (Sigma) in full medium for 2 hr before incubation with EBSS + 50 μM Nocodazole followed by imaging.

After time-lapse microscopy, cells were immediately fixed in 3% paraformaldehyde and processed for confocal microscopy as detailed above. Photoconverted cells were located using gridded MatTek dishes. During 4-colour imaging (Fig. 6C), 405 nm illumination was carried out only after acquisition of the other fluorophore signals, in order to prevent artefactual photoconversion.

Statistical analysis

Statistics were performed using GraphPad Prism 6 software, as detailed in the figure legends.

Primers used in this study

Primer	Description	Sequence (5'-3')
WAC SDM Primer 1	Silent mutations To make WAC (Q9BTA9-1) resistant to WAC-03 siRNA	GAA AAG AAT CTA CAT CAG GTG ATA AAC CCG TAT CAC ATT C
WAC SDM Primer 2	Silent mutations To make WAC (Q9BTA9-1) resistant to WAC-03 siRNA	CTA CAT CAG GTG ATA AGC CGG TAT CGC ATT CTT GCA CAA CTC CTT C
WAC SDM I626S L629S	Mutations of hydrophobic 'a' and 'd' positions in WAC CC domain to remove RNF40 binding	TTG CGA GAG CAA AGG AGC CTA TTT TCG AGA CAA CAA ATT AAG
GABARAP SDM primer	To make human GABARAP G116A mutation	GAC GAA AGT GTC TAC GCT CTG TGA AGC TGC TCG

References

Axe, E.L., Walker, S.A., Manifava, M., Chandra, P., Roderick, H.L., Habermann, A., Griffiths, G., and Ktistakis, N.T. (2008). Autophagosome formation from membrane compartments enriched in phosphatidylinositol 3-phosphate and dynamically connected to the endoplasmic reticulum. J Cell Biol *182*, 685-701.

Chan, E.Y., Longatti, A., McKnight, N.C., and Tooze, S.A. (2009). Kinase-inactivated ULK proteins inhibit autophagy via their conserved C-terminal domain using an Atg13-independent mechanism. Mol Cell Biol *29*, 157-171.

Cox, J., and Mann, M. (2008). MaxQuant enables high peptide identification rates, individualized p.p.b.-range mass accuracies and proteome-wide protein quantification. Nat Biotechnol *26*, 1367-1372.

Du, P., Kibbe, W.A., and Lin, S.M. (2008). lumi: a pipeline for processing Illumina microarray. Bioinformatics *24*, 1547-1548.

Gantke, T., Boussouf, S., Janzen, J., Morrice, N.A., Howell, S., Muhlberger, E., and Ley, S.C. (2013). Ebola virus VP35 induces high-level production of recombinant TPL-2-ABIN-2-NF-kappaB1 p105 complex in co-transfected HEK-293 cells. Biochem J *452*, 359-365.

Kraft, C., Kijanska, M., Kalie, E., Siergiejuk, E., Lee, S.S., Semplicio, G., Stoffel, I., Brezovich, A., Verma, M., Hansmann, I.*, et al.* (2012). Binding of the Atg1/ULK1 kinase to the ubiquitin-like protein Atg8 regulates autophagy. EMBO J *31*, 3691-3703.

McKnight, N.C., Jefferies, H.B., Alemu, E.A., Saunders, R.E., Howell, M., Johansen, T., and Tooze, S.A. (2012). Genome-wide siRNA screen reveals amino acid starvation-induced autophagy requires SCOC and WAC. EMBO J *31*, 1931-1946.

Polson, H.E.J., de Lartigue, J., Rigden, D.J., Reedijk, M., Urbe, S., Clague, M.J., and Tooze, S.A. (2010). Mammalian Atg18 (WIPI2) localizes to omegasome-anchored phagophores and positively regulates LC3 lipidation. Autophagy *6*, 506-522.

Poser, I., Sarov, M., Hutchins, J.R., Heriche, J.K., Toyoda, Y., Pozniakovsky, A., Weigl, D., Nitzsche, A., Hegemann, B., Bird, A.W.*, et al.* (2008). BAC TransgeneOmics: a high-throughput method for exploration of protein function in mammals. Nat Methods *5*, 409-415.

Ritchie, M.E., Phipson, B., Wu, D., Hu, Y., Law, C.W., Shi, W., and Smyth, G.K. (2015). limma powers differential expression analyses for RNA-sequencing and microarray studies. Nucleic Acids Res *43*, e47.

Schwanhausser, B., Busse, D., Li, N., Dittmar, G., Schuchhardt, J., Wolf, J., Chen, W., and Selbach, M. (2011). Global quantification of mammalian gene expression control. Nature *473*, 337-342.

Totsukawa, G., Kaneko, Y., Uchiyama, K., Toh, H., Tamura, K., and Kondo, H. (2011). VCIP135 deubiquitinase and its binding protein, WAC, in p97ATPase-mediated membrane fusion. Embo J *30*, 3581-3593.

Young, A.R.J., Chan, E.Y.W., Hu, X.W., Köchl, R., Crawshaw, S.G., High, S., Hailey, D.W., Lippincott-Schwartz, J., and Tooze, S.A. (2006). Starvation and ULK1-dependent cycling of Mammalian Atg9 between the TGN and endosomes. J Cell Sci *119*, 3888-3900.
